# Terminal repeats impact collagen triple-helix stability through hydrogen bonding†

**DOI:** 10.1039/d2sc03666e

**Published:** 2022-10-20

**Authors:** Yingying Qi, Daoning Zhou, Julian L. Kessler, Rongmao Qiu, S. Michael Yu, Gang Li, Zhao Qin, Yang Li

**Affiliations:** Guangdong Provincial Key Laboratory of Biomedical Imaging and Guangdong Provincial Engineering Research Center of Molecular Imaging, The Fifth Affiliated Hospital, Sun Yat-sen University Zhuhai 519000 China liyang266@mail.sysu.edu.cn; Cardiac Surgery and Structural Heart Disease Unit of Cardiovascular Center, The Fifth Affiliated Hospital, Sun Yat-sen University Zhuhai 519000 China gangli73@163.com; Department of Radiology, The Fifth Affiliated Hospital, Sun Yat-sen University Zhuhai 519000 China; Department of Biomedical Engineering, University of Utah Salt Lake City Utah 84112 USA; Department of Civil & Environmental Engineering, College of Engineering & Computer Science, Syracuse University Syracuse New York 13244 USA zqin02@syr.edu

## Abstract

Nearly 30% of human proteins have tandem repeating sequences. Structural understanding of the terminal repeats is well-established for many repeat proteins with the common α-helix and β-sheet foldings. By contrast, the sequence–structure interplay of the terminal repeats of the collagen triple-helix remains to be fully explored. As the most abundant human repeat protein and the most prevalent structural component of the extracellular matrix, collagen features a hallmark triple-helix formed by three supercoiled polypeptide chains of long repeating sequences of the Gly–X–Y triplets. Here, with CD characterization of 28 collagen-mimetic peptides (CMPs) featuring various terminal motifs, as well as DSC measurements, crystal structure analysis, and computational simulations, we show that CMPs only differing in terminal repeat may have distinct end structures and stabilities. We reveal that the cross-chain hydrogen bonding mediated by the terminal repeat is key to maintaining the triple-helix's end structure, and that disruption of it with a single amide to carboxylate substitution can lead to destabilization as drastic as 19 °C. We further demonstrate that the terminal repeat also impacts how strong the CMP strands form hybrid triple-helices with unfolded natural collagen chains in tissue. Our findings provide a spatial profile of hydrogen bonding within the CMP triple-helix, marking a critical guideline for future crystallographic or NMR studies of collagen, and algorithms for predicting triple-helix stability, as well as peptide-based collagen assemblies and materials. This study will also inspire new understanding of the sequence–structure relationship of many other complex structural proteins with repeating sequences.

## Abstract 2

Collagen mimetic peptides (CMPs) only differing in terminal repeat have distinct stabilities and end structures due to a spatial hydrogen bonding profile that is useful for future crystallography, algorithm prediction, and materials of collagen.

## Graphical abstract

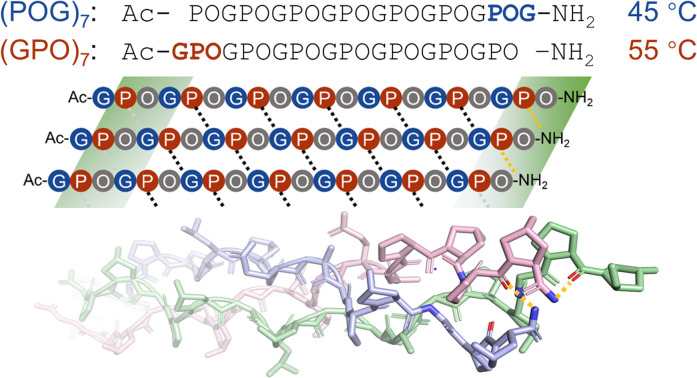

## Introduction

From single amino acids to domains of over 100 residues, tandem repeating sequences are present in almost 30% of human proteins.^1^ Many repeat proteins play essential roles in both basic molecular recognition and pathological aggregation.^2,3^ From the ankyrin repeats and leucine zippers to the β-propellers, elucidation of the sequence–structure relationship of these modular foldings is enabled by designed oligomers of individual repeats.^4–7^ The external repeats at the N- and C-ends of these proteins, often called the terminal capping repeats, can have general folding similar to the internal repeats, and are often carefully studied and engineered for the proteins' overall solubility and stability.^8^ Furthermore, for individual repeats or modules, such as the common α-helix and β-sheet folding, there is well-established structural understanding of their terminal residues.^9–13^ Studies of these local capping motifs have promoted understanding of the terminal and boundary structures of the repeat proteins, and inspired novel designs of engineered nanostructures and self-assembling biomolecules.^14–16^ By contrast, there has been limited exploration of terminal capping for repeat proteins not constructed with α-helices or β-sheets, such as the collagen triple-helix.

**Fig. 1:**
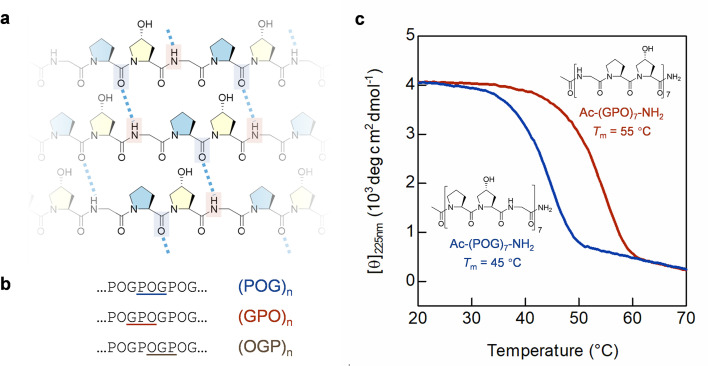
The repetitive sequence and structure of collagen are modeled by the collagen mimetic peptides (CMPs). (a) The molecular structure of the collagen triple-helix: since Pro and Hyp both lack the N-hydrogen atom, interchain H-bonds (dotted lines) can only form between the amide of Gly (red box) and the carbonyl of Pro (blue box). (b) The three forms of repeating triplet of a typical CMP sequence: (POG)_*n*_, (GPO)_*n*_, and (OGP)_*n*_. (c) Under the same testing condition, CD thermal unfolding curves show that CMP 1 [Ac–(POG)_7_–NH_2_] is 10 °C less stable than CMP 2 [Ac–(GPO)_7_–NH_2_], even with the almost identical sequences.

The sequence and folding of collagen are defined by repetition. As the most abundant mammalian protein, the fundamental structure of collagen, the triple-helix, is formed by three interwinding polypeptide chains, each consisting of a long repetitive sequence of Gly–X–Y triplets, where X and Y are often proline (Pro, P) and hydroxyproline (Hyp, O), respectively.^17^ Interchain hydrogen-bonding (H-bonding) between the amide of Gly and the carbonyl of Pro stabilizes the triple-helix (Fig. 1a).^18^ For decades, collagen mimetic peptides (CMP), a series of short peptides with 6–10 repeating triplets (*i.e.*, often made up by G, P, O), have been employed as models for understanding the structures and functions of the massive, insoluble natural collagens.^18–21^

Despite collagen's unique structure and important functions in almost every human tissue type,^17^ unlike the well-studied coiled-coil,^22^ the sequence–structure relationship for the terminal repeats of a collagen triple-helix remains unknown. The repeating triplet of a canonical CMP sequence can take three forms: POG, GPO, and OGP (Fig. 1b). Of these, only (POG)*_n_* and (GPO)_*n*_ are traditionally used in collagen research.^18,21,23,24^ Interestingly, in the UniProt database, the recognized triple-helix regions of most types of human collagen chains are both initiated and terminated as GXY, rather than XYG (Table S1†). Nonetheless, the two CMP formulae are assumed interchangeable, meaning that CMP triple-helices with equal repeats of the POG- and GPO-triplets are considered identical in structural stability. Inconsistencies in reported thermal denaturation temperature of CMPs [*e.g.*, (POG)_8_: 50.5 °C *vs.* (GPO)_8_: 44.5 °C],^25,26^ though sometimes significant [*e.g.*, (POG)_7_: 43 °C *vs.* (GPO)_7_: 55 °C],^24,27^ are often attributed to terminal functional groups and charges,^27^ peptide concentrations, as well as methods and errors from different measurements, including heating rates.^18,28^ So, are these collagen repeats indeed structurally equivalent, or can they make terminal cappings with different characteristics?

Here we investigate whether and how the CMP triple-helices with different terminal repeats differ in structure and stability. With CD characterization of 28 CMPs with variable terminal motifs (Table S2†), as well as crystal structure analysis and computational simulations, we reveal that the interchain H-bonding mediated by the terminal repeat is key to the structural disorder of the helices' ends, and that disruption of it by a single change in the functional group can cause destabilization as drastic as 11–19 °C in denaturation temperature. Our results indicate a fresh spatial profile of H-bonding within the collagen triple-helix, which will not only contribute to future designs of collagen model peptides, assemblies, and materials,^21,29–31^ but also inspire new understandings of the sequence–structure relationship of many other complex repeat proteins.^1^

## Results

### GPO *vs.* POG

In this study, we first validated that the peptide length and instrument heating rate can affect the CMPs' thermal stability (Fig. S1 and S2†). We monitored the stability by circular dichroism (CD), where a CMP triple-helix dissociated into single chains under gradual heating, and the steepest point of this two-state transition curve is defined as the melting temperature (*T*_m_, see Methods). To avoid measurement bias or errors, we carefully prepared CMP 1 [Ac–(POG)_7_–NH_2_] and 2 [Ac–(GPO)_7_–NH_2_] and examined their triple-helix stability under the same condition. Despite their identical chain length and amino acid composition, the CD melting curves showed that the *T*_m_ value of CMP 2 is 10 °C higher than that of CMP 1 (Fig. 1c). More strikingly, the *T*_m_ value of every Ac–(POG)_*n*_–NH_2_ sequence (*n* = 5–9) is at least 7 °C lower than its GPO counterpart in the series (Fig. S2†).

### The terminal Gly

**Fig. 2:**
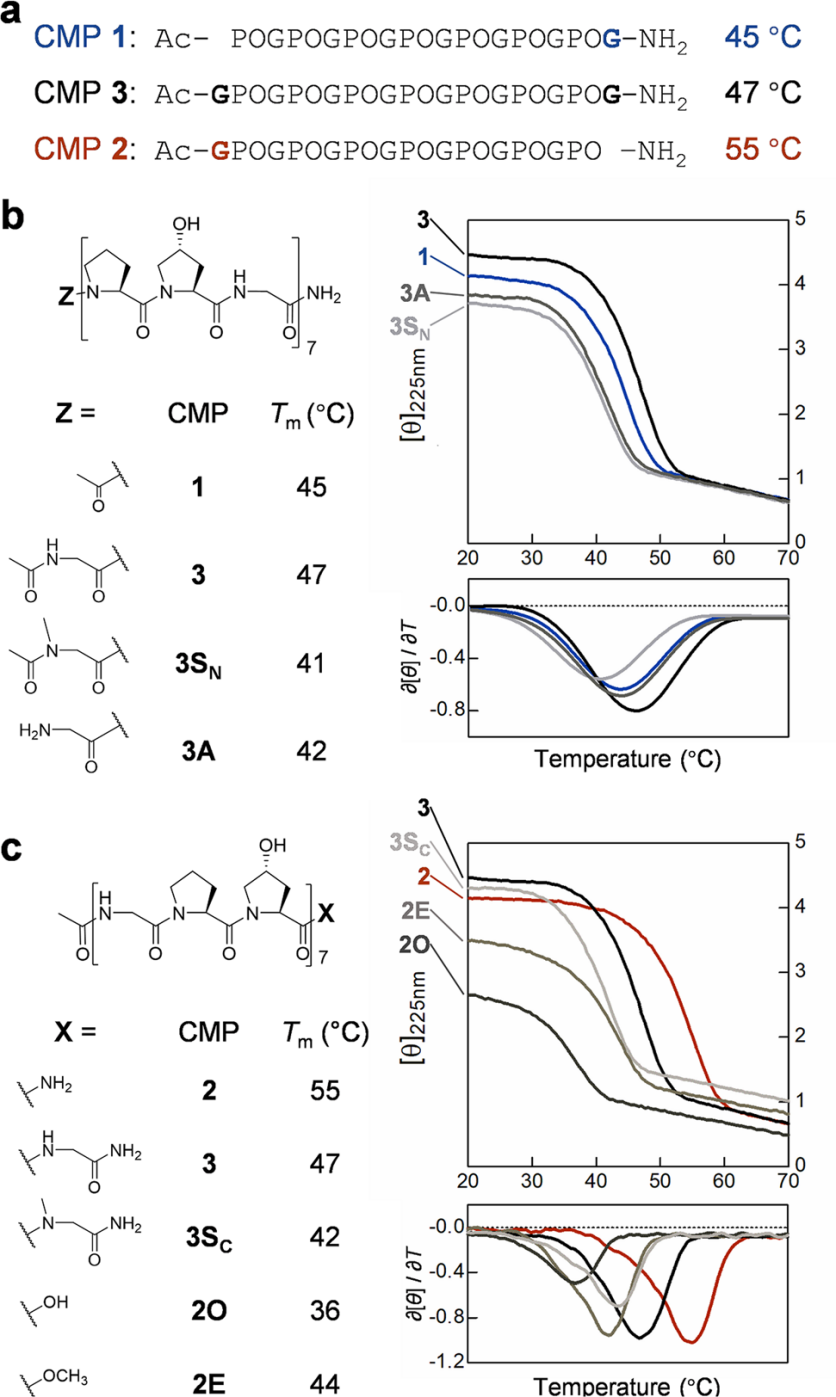
The C-terminal Gly weakens the triple-helix stability of CMP. (a) The sequences and *T*_m_ values of CMP 1, 2, and 3. (b) The structures, CD thermal unfolding curves (right, top) and their first derivatives (right, bottom), as well as *T*_m_ values of CMP 1, 3, 3S_N_, and 3A (featuring various N-terminal moieties). (c) The structures, CD thermal unfolding curves (right, top) and their first derivatives (right, bottom), as well as *T*_m_ values of CMP 2, 3, 3S_C_, 2O, and 2E (featuring various C-terminal moieties). Unit of CD [*θ*]_225nm_: 10^3^ deg cm^2^ dmol^−1^.

The sequence difference between CMP 1 and 2 only lies at two ends: CMP 1 has an extra C-terminal Gly while CMP 2 has an extra N-terminal one (Fig. 2a). To clarify the effect of each terminal Gly on the triple-helix stability, we made CMP 3, featuring Gly at both termini (Fig. 2a). The *T*_m_ value of CMP 3 (47 °C) was only 2 °C higher than CMP 1 (Fig. 2b), suggesting that the extra N-terminal Gly makes almost no contribution to stability. To test whether the extra Gly adds H-bonds, we designed two CMPs that are deficient in H-bond donation at the N-termini: CMP 3S_N_ features an *N*-acetylated sarcosine (Sar) residue which lacks the amide hydrogen, and CMP 3A has a terminal amine which creates interchain charge-repulsion at physiological pH (Fig. 2b). The *T*_m_ values of CMP 3S_N_ and 3A were 41–42 °C, which were not far from CMP 1 and 3 (Fig. 2b). Considering that CMP 3S_N_ and 3A also involve other destabilizing factors at the N-terminal (steric and charge repulsions), these results suggested that the N-terminal Gly contributes very weakly to interchain H-bonding and the triple-helix stability.

At the C-terminus, even with one more residue in sequence, the *T*_m_ value of CMP 3 was 8 °C lower than CMP 2 (Fig. 2a and c), indicating that the additional C-terminal Gly strongly *destabilizes* the triple-helix in CMP 1 and 3. Next, we made CMP 3S_C_, 2O, and 2E, all with little or no capability to form the C-terminal most interchain H-bond: CMP 3S_C_ features *N*-methylated Sar and CMP 2E is capped with a hydrogen-deficient ester, while CMP 2O ends with a negatively-charged carboxyl group at physiological pH (Fig. 2c). The *T*_m_ values of CMP 3S_C_, 2O, and 2E were all drastically lower than CMP 2 (Δ*T*_m_: 11–19 °C). Amazingly, with the substitution of just one functional group at the C-end (*i.e.*, CONH_2_ → COOCH_3_), the triple-helix stability decreased by 10 °C (CMP 2*vs.*2E). These results suggested the C-terminal Hyp-amide highly likely contributes to new H-bonding that stabilizes CMP 2. Furthermore, our data indicated that completely abolishing the C-terminal H-bonding and inducing local sterics with Sar destabilizes the triple-helix by 13 °C (Fig. 2c, CMP 2*vs.*3S_C_), while attaching C-terminal Gly destabilizes the helix by 8 °C (CMP 2*vs.*3). These results suggested that the C-terminal Hyp–HN–Gly in CMP 3 and 1 probably only forms a particularly weak interchain H-bond.

### Crystal structures

**Fig. 3:**
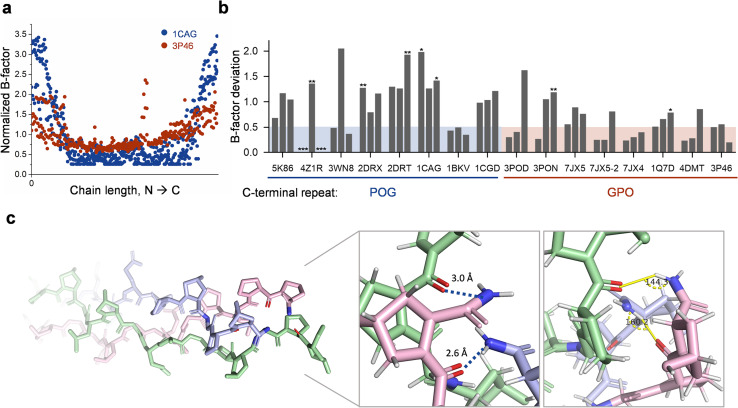
Analysis of crystal structures of CMP triple-helices in PDB suggests lower flexibility and possible H-bonding in GPO-based C-termini. (a) B-factors of all non-hydrogen atoms along each CMP triple-helix, normalized by the mean B-factor of the given structure for PDB entry 1CAG [(POG)_4_POA(POG)_5_] and 3P46 [(GPO)_2_GLOGEA(GPO)_2_]. (b) The B-factor deviations of the C-terminal amino acid triplet (POG *vs.* GPO) from the mean B-factor of all atoms in a given triple-helix. Each * indicates one un-resolved and missing C-terminal amino acid residue in the crystal structure. (c) Crystal structures of the GPO-featuring C-terminal of 3P46, showcasing the optimal bond distances and angles between the C-terminal most Hyp–CONH_2_ and Pro–C=O for the characteristic collagen interchain H-bonding.

Next, we surveyed existing crystal structures of CMP triple-helices in the Protein Data Bank (PDB, see Table S3†) to search for evidence of structural differences between CMPs with POG- and GPO-terminal repeats.^23,24,32–41^ We analyzed the B-factor of each CMP structure as it often correlates with the flexibility and internal motion in protein crystallography.^42^ We plotted normalized B-factors of all non-hydrogen atoms along each CMP triple-helix: while all structures have elevated structural flexibility at the termini, a general trend of higher terminal B-factor was noted for the POG-sequences (Fig. 3a, S3 and S4†). We calculated the N- and C-ending amino acid triplet's B-factor deviation from the mean B-factor of all atoms in a given triple-helix of all crystal structures (C-terminal: Fig. 3b, N-terminal: Fig. S5,† see Methods). The deviation values showed that the POG-CMPs have higher flexibility than the GPO ones at the C-termini. We also noted that the crystal structures of the POG-sequences are more likely to have unresolved or missing terminal residues than the GPO ones (Fig. 3b, asterisks, Table S3†), further implying that the POG-ended C-termini may be more disordered. Finally, we noted that the distances and angles between the C-terminal Hyp–NH_2_ and Pro–C=O are suitable for creating interchain H-bonds in multiple GPO crystal structures ending with Hyp-amide (Fig. 3c and Table S3†).

### Molecular dynamics (MD) simulations

**Fig. 4:**
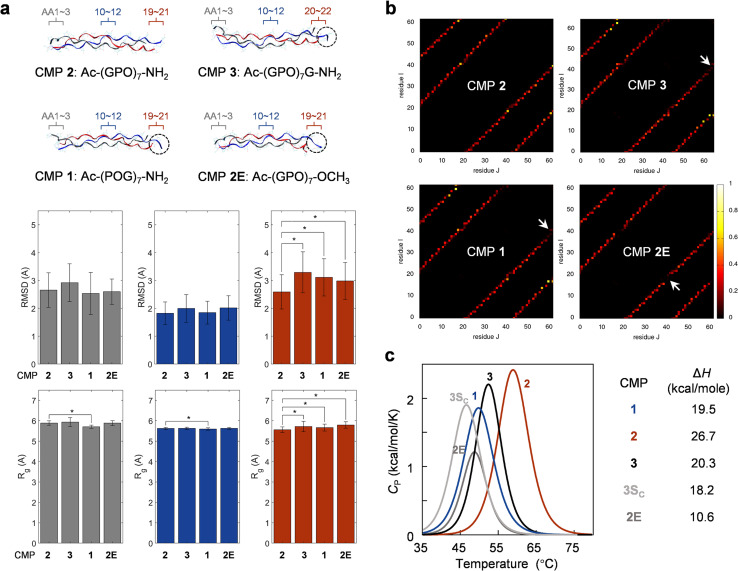
Structural analysis of CMPs by fully atomistic molecular dynamics simulations and differential scanning calorimetry (DSC). (a) The sequences and the relaxed final molecular structures of CMP 2, 3, 1, and 2E with the loose C-terminal structures circled. Computed root-mean-square deviation (RMSD, top row) and the radius of gyration (*R*_g_, bottom row) of all atoms within the N-terminal (residue 1–3 for each of the three chains, gray), central (residue 10–12 for each of the three chains, blue) and C-terminal triplets (residue 20–22 for each of the three chains in CMP 3, residue 19–21 in all other CMPs, red). The asterisk: *P* < 0.001 (two-sample *t*-test, details in Methods). (b) Heat plots for the time-average count of the number of H-bonds between any two residues within the four CMPs, as monitored during 20 ns equilibrium simulations. Arrows point to the residues missing interchain H-bonds. (c) The DSC thermal denaturation curves and enthalpy changes (Δ*H*) of CMP 1, 2, 3, 3S_C_, and 2E indicate greater interchain H-bonding in CMP 2.

To further understand the CMP difference in terminal flexibility and thermal stability, we used fully atomistic MD simulations to build CMP 1, 2, 3, and 2E and fully relaxed them (see details in Methods).^43,44^ We computed the root-mean-square deviation (RMSD) and radius of gyration (*R*_g_) of amino acid triplets at representative locations, namely the acetylated N-terminus, the triplet in the center, and the C-terminus of interest (Fig. 4a, S6 and S7†). The RMSD value measures the mean deviation of each atom within the region from its initial conformation, and it is used to quantify random migration because of thermal fluctuation. *R*_g_ measures the mean size of the atoms within the region. The similar RMSD and *R*_g_ values for the four CMPs at the N-terminus and center suggest that they have very similar dynamics and size during simulation (Fig. 4a); this is expected as the four CMPs share the same or similar chemical structures at these two locations. However, the RMSD of CMP 2 at the C-terminus is significantly lower and *R*_g_ is significantly smaller than the other three CMPs, suggesting the C-terminus of CMP 2 (*i.e.*, Hyp–CONH_2_) moves less during the thermal fluctuation and keeps a more compact size (Fig. 4a). This result correlates nicely with our observation of the relatively lower B-factors for the CMPs with the C-terminal GPO repeat (Fig. 4a).

We also compared the distribution of the H-bonds as the time-average number of H-bonds between any pair of the residues within these CMPs (Fig. 4b). It was shown that CMP 2 has H-bonds homogenously distributed along each of the three chains with strong H-bonds near the C-termini (yellow spots), while the other three sequences have missing H-bonds during the relaxation at their C-termini (arrows). For example, CMP 3 misses the interchain H-bonding between chain 2 and 3 (at residue 44 and 66), while CMP 1 misses H-bonding between chain 2 and 3 (at residue 42 and 63), and CMP 2E misses H-bonding between chain 1 and 2 (at residue 21 and 42). The pattern of the missing H-bonds corresponds to the partially loose structure at the C-termini of these three CMP molecules, as shown by the relaxed molecular structure: two of the three CMP chains are tightly bonded while the third one is not (Fig. 4a, dotted circles). Together, our simulations supported that except for CMP 2, these triple-helices (with either HypGly–CONH_2_ or Hyp–COOCH_3_ as end-moiety) have weakened H-bonds and loose structures at the C-termini.

### Differential scanning calorimetry (DSC)

To directly interrogate whether CMP 2 has greater interchain H-bonding, we obtained the thermal denaturation curves of CMP 1, 2, 3, 3S_C_, and 2E using DSC, and measured the enthalpy change (Δ*H*) for each peptide (Fig. 4c and S8,† see Methods).^45,46^ CMP 2 showed the highest Δ*H* value, which was 6.4 kcal mol^−1^ higher than CMP 3, and 7.2 kcal mol^−1^ higher than CMP 1. Also, the Δ*H* value of CMP 3 was close to CMP 3S_C_, which lacks the C-terminal H-bonding due to *N*-methylation. All of these data are in line with our CD *T*_m_ measurements and support that the C-terminal Hyp–CONH_2_ of CMP 2 is engaged in interchain H-bonds which are weakened with the appendant Gly in CMP 3. Meanwhile, the Δ*H* value of CMP 1 was almost the same as CMP 3, also supporting that the extra N-terminal Gly in CMP 3 barely contributes to stability.

### Terminal Pro and Hyp residues

**Fig. 5:**
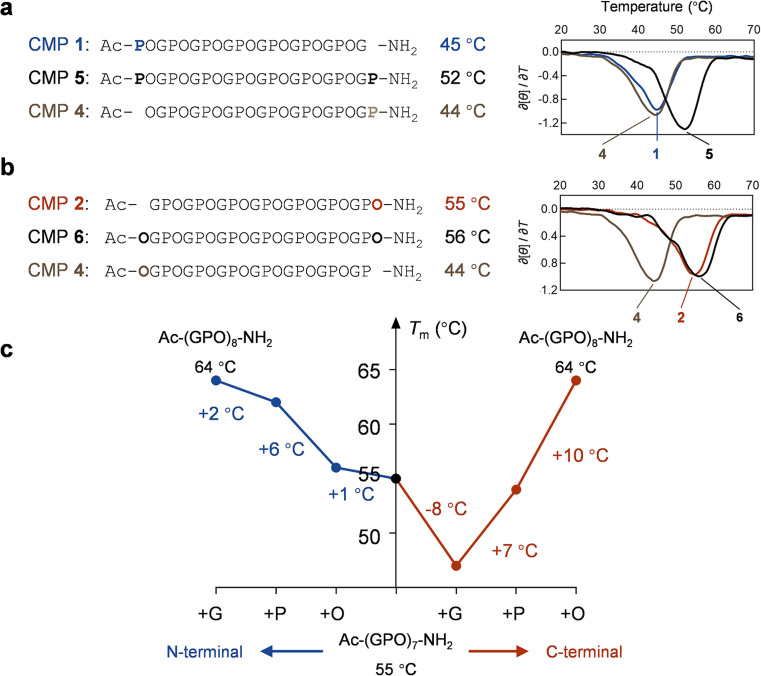
Effect of terminal Pro and Hyp on CMP stability. (a and b) The sequences, *T*_m_ values, and the first derivatives of CD thermal unfolding curves of CMP 1, 2, 4, 5, and 6: adding Pro to either the N- or C-terminus, or adding Hyp to the C-terminus can greatly stabilize the triple-helix. (c) The *T*_m_ changes towards Ac–(GPO)_8_–NH_2_ with stepwise attachments of O, P, and G to the N- or C-terminus of CMP 2.

Using the approach described in Fig. 2, we studied the structural effects of Pro and Hyp on each end (Fig. 5). For Pro, the *T*_m_ comparisons indicated that an extra Pro at either N- or C-terminus can stabilize the triple-helix by 7–8 °C (Fig. 5a). For Hyp, while adding Hyp to the N-terminus had little contribution to stability (Δ*T*_m_ = 1 °C), incorporating a C-terminal Hyp can raise the *T*_m_ by 12 °C (Fig. 5b). After studying the effect of the terminal residue on the thermal stability of CMPs of the same length, we measured the CMP stability change during incremental sequence extension from (GPO)_7_ to (GPO)_8_ for both N- and C-directions (Fig. 5c). By sequentially adding O, P, and G residues from the N-terminal, we found that the greatest *T*_m_ increase occurred with Pro (Fig. 5c, left). At the C-terminal, adding Pro compensated the *T*_m_ fall caused by Gly, while the biggest jump in *T*_m_ came with Hyp (Fig. 5c, right). We conducted additional measurements for (POG)_7_ → (POG)_8_ and (OGP)_7_ → (OGP)_8_ and obtained data in line with Fig. 5c (Fig. S9 and S10†).

### A hydrogen-bonding map

**Fig. 6:**
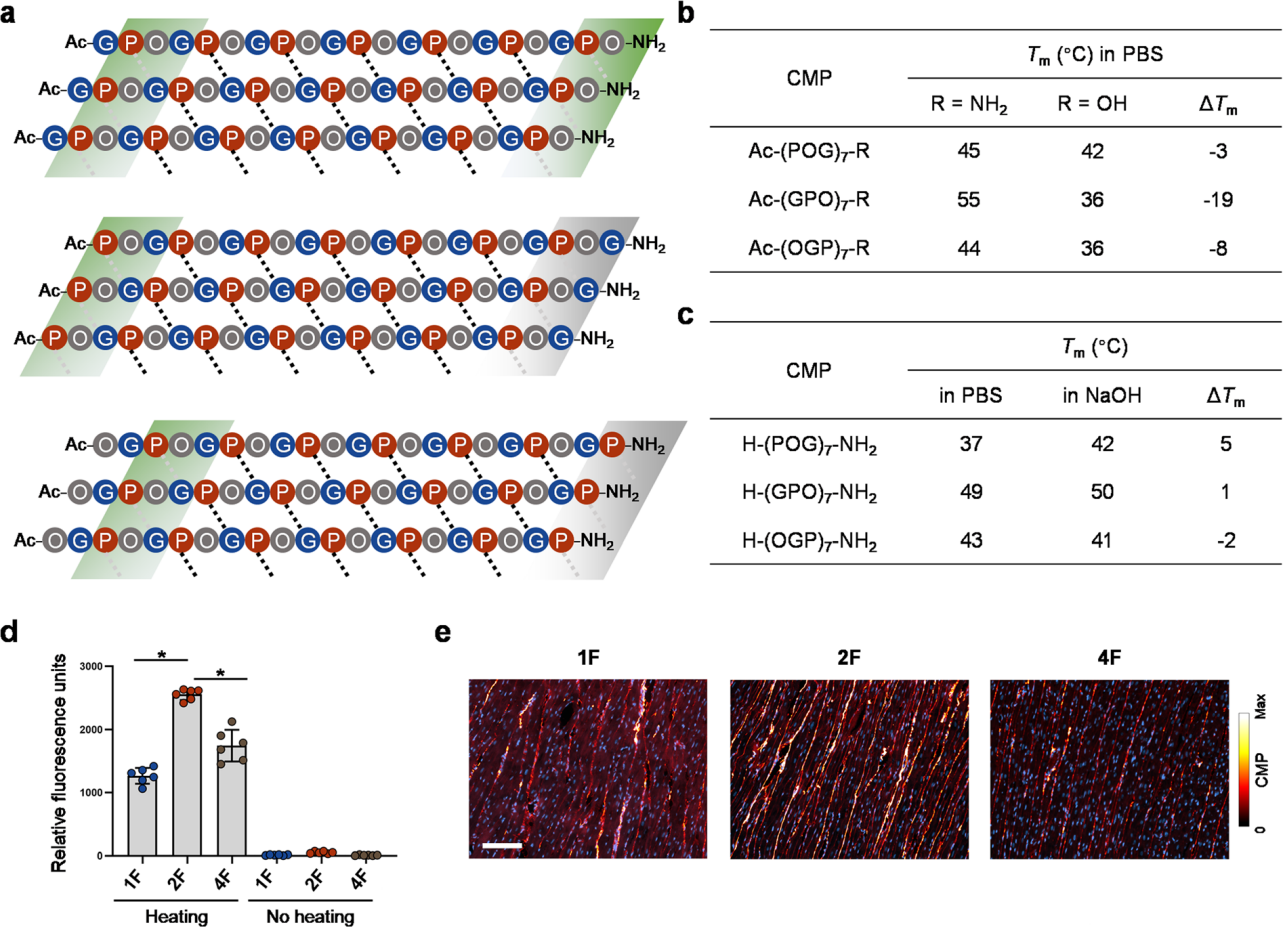
CMPs featuring varying terminal repeats differ in H-bonding pattern and collagen-hybridizing propensity. (a) A schematic map of plausible interchain Pro–C=O⋯HN–Gly H-bonding contacts for CMP 2, 1, and 4 triple-helices (black dotted lines: internal contacts; gray dotted lines: terminal contacts that may or may not form stable H-bonds). According to our data, the contacts within the green blocks can establish stable H-bonds to certain extent, whereas the ones in the gray blocks can only form weak or no H-bonds due to the lack of Gly as the H-bond donor (CMP 4) or interference from the terminal flexible Gly (CMP 1). (b) The impact of the charged C-terminal carboxylate on the stability of CMPs with different terminal repeats: GPO > OGP > POG. (c) The effect of the charged N-terminal amine on the stability of CMPs with different terminal repeats: POG > GPO > OGP (PBS, pH 7.4; NaOH, pH 11.5). (d and e) Fluorescently-labeled single-strand CMP 2F [CF–Ahx–(GPO)_7_–NH_2_, CF: carboxyfluorescein, Ahx: aminohexanoic acid] binds to denatured collagen more strongly than CMP 1F [CF–Ahx–(POG)_7_–NH_2_] and 4F [CF–Ahx–(OGP)_7_–NH_2_] on gelatin-coated plates (d) and thermally antigen-retrieved, paraffin-embedded sections of rat heart tissue (e). By contrast, triple-helical CMP 1F, 2F, and 4F showed no affinity to gelatin-coated plates (no heating group, d). (d) Asterisk: significant difference in means (*P* < 0.01, one-way ANOVA with *post hoc* Tukey HSD test). (e) Blue: DAPI; scale bar: 100 μm.

Based on the simulation, DSC, and all *T*_m_ data (Fig. 2–5 and Table S2†), a schematic map of possible interchain Pro–C=O⋯HN–Gly H-bond patterns can be sketched for the three CMP models with different repeating units (Fig. 6a). For these *N*-acetylated peptides, the main difference lies in the C-terminal regions. For Ac–(OGP)_7_–NH_2_ (CMP 4), the last Pro⋯Gly H-bonds cannot form due to lack of the Gly H-bond donor; for Ac–(POG)_7_–NH_2_ (CMP 1), although the C-terminal Pro–C=O could bond with the ending HN–Gly, the flexible Gly apparently interferes this interaction (Fig. 2–4). In contrast, for Ac–(GPO)_7_–NH_2_ (CMP 2), “extra” C-terminal-most H-bonds can possibly form between Pro's carbonyl and Hyp's ending NH_2_ group (Fig. 3c and 4c), resulting in the peptide's higher triple-helix stability.

This H-bonding map can help explain the inconsistent effects of the terminal charges on the three CMP sequences. For example, substituting a neutral C-terminal amide with a negatively-charged carboxyl group in (GPO)_7_ may abolish the extra C-terminal H-bonding in CMP 2 (green block, Fig. 6a), thus dramatically lowering the *T*_m_ value by 19 °C, far exceeding Δ*T*_m_ values of the other two counterparts (Fig. 6b). At the unacetylated N-terminal, it can be expected that positive charge repulsion destabilizes the POG sequence the most (Fig. 6c) since only when Pro is the N-terminal most residue, the end charge repulsion can directly weaken the interchain H-bonding (Fig. 6a, note the locations of the three N-terminal green blocks).

### CMP-collagen hybridization

We previously reported that CMP single-strands can bind to and form hybridized triple-helices with unfolded natural collagen chains in pathological tissues and denatured collagen materials (*i.e.*, gelatin).^47–50^ The collagen hybridization is strongly driven by the triple-helix folding propensity of the CMPs. To test whether CMPs only different in terminal repeat can bind to denatured collagen with the same affinity, we prepared carboxyfluorescein-labeled CMP 1, 2, and 4 (designated as 1F, 2F, and 4F) and compare their binding to unfolded collagen on gelatin-coated assay plates (Fig. 6d) and paraffin-embedded sections of rat hearts (Fig. 6e). To enable CMP-collagen hybridization, the F-CMPs were dissociated to single-strands by heating at 85 °C before binding, and the heart sections had undergone heated-mediated antigen-retrieval to completely denature their collagen content (see Methods).^48^ We found that, on both the gelatin coating and the heart sections, CMP 2F showed the highest affinity to denatured collagen, followed by CMP 4F and 1F (Fig. 6d and e). In the heart sections co-stained with CMP 2F and an anti-collagen I antibody, the positive CMP and antibody signals strongly overlapped (Fig. S11†), validating the peptide's high specificity to collagen. These results demonstrated that the GPO-featuring CMP 2F has the strongest triple-helical folding propensity among the three forms during CMP-collagen hybridization.

## Discussion

Our main finding is that the interchain H-bonding determines the structure of CMP's different terminal repeats. Previous crystallographic studies of CMP triple-helices revealed that the terminal amino acids often lack interchain H-bonding and splay away from the core helical axis, giving them higher mobilities and B-factors.^33,51–53^ Comparative NMR analysis of Ac–(POG)_10_–NH_2_ also revealed stretches of disorder as wide as six amino acids at the C-terminus.^51^ Interestingly, these reports were predominantly based on the POG-repeating sequences. In addition to supporting these prior findings (Table S3†), our study discovered that the GPO-repeating sequences can form an extra set of stabilizing inter-helix H-bonds at the C-terminal. As evidence, the *T*_m_ gap between Ac–(POG)_7_–NH_2_ and Ac–(GPO)_7_–NH_2_ is 10 °C (Fig. 1), which is essentially equal to the *T*_m_ increase gained by adding a triplet unit [Fig. S2,† Ac–(POG)_7_–NH_2_ → Ac–(POG)_8_–NH_2_: 45 → 56 °C; Ac–(GPO)_7_–NH_2_ → Ac–(GPO)_8_–NH_2_: 55 → 64 °C]. Second, it was reported that substituting one Gly to aza-Gly, a synthetic residue that can form one additional cross-chain H-bond, also increases the *T*_m_ value of (POG)_7_ by 11 °C.^54^ Third, the energy of an inter-helix Pro⋯Gly H-bond was estimated as 2.0 kcal mol^−1^,^55^ while unfolding Δ*H* value of Ac–(GPO)_7_–NH_2_ was 7 kcal mol^−1^ greater than Ac–(POG)_7_–NH_2_ (Fig. 4c), comparable to three H-bonds. These reported results are well in line with our data, supporting the creation of extra H-bonds by the C-terminal Hyp–CONH_2_.

All our data suggest the flexible Gly as the cause of POG's inability to form stable H-bonds at the C-terminus (Fig. 2–6). Because Pro and Hyp both lack the N-hydrogen atom, Gly is the sole interchain H-bond donor in the whole triple-helix (Fig. 1a).^18^ Unlike salt bridges that can spontaneously form by electrostatic attraction from any direction, H-bond formation requires the participating functional groups to be within proper distances and angles. In the central triplets, the Hyp and Pro flanking a Gly residue ensure the peptide's polyproline-II-helix conformation, thereby offering the proper angle for Gly to form the interchain H-bond. However, it can be envisioned that at the C-terminus, with reduced conformational restrictions, Gly exhibits a high degree of disorder and lacks a defined backbone structure (see MD simulation in Fig. S12†), which can lead to H-bond disruption. This may also explain why adding Pro to the C-terminal Gly recovers the *T*_m_ value by 7–8 °C (Fig. 5, S9 and S10†). Based on ^1^H–^15^N NMR experiments, a concurrent study on the similar topic also suggested the Gly flexibility at the N- and C-termini.^56^ Meanwhile, although the interchain H-bond is formed by the carbonyl of Pro and the amine of Gly (Fig. 1a, red and blue boxes), these two functional groups are covalently connected by the Hyp in-between (*i.e.*, ⋯O=C–Hyp–NH⋯) at the Y position. Thereby the conformation of Hyp, which induces specific backbone folding, can directly affect the bond angles of all interchain H-bonds within the collagen triple-helix. This provides a structural insight for post-translational hydroxylation of Pro that almost exclusively occurs at the Y position of natural collagen chains.^17^

Given the several variables we examined in this work, including the sequence and length (Fig. S2†), the terminal residue (Fig. 2 and 5) and the charge (Fig. 6b and c), as well as the CD heating rate (Fig. S1†), it is possible to explain the various *T*_m_ values of similar CMPs from our study and earlier publications (See Table S4† for example).^57^ More importantly, based on our findings, conflicting results from previous reports can now be reconciled with the terminal repeat argument [*e.g.*, (POG)_7_: 43 °C *vs.* (GPO)_7_: 55 °C].^24,27^ The N-termini of most existing POG-based crystal structures are disordered (Table S3†), probably because those N-terminal Pro residues are not acetylated, resulting in charge repulsion disrupting the H-bonding (Fig. 6a). Meanwhile, it was recently reported that the positive charges of ammonium groups destabilize the triple-helix [*e.g.*, H–(POG)_7_–NH_2_, pH 7.4 *vs.* 10.6, Δ*T*_m_ = 6 °C] to a greater extent than the negative charges of carboxylate groups [*e.g.*, Ac–(POG)_7_–NH_2_*vs.* Ac–(POG)_7_–OH, Δ*T*_m_ = 3 °C at pH 7.4].^27^ This discrepancy is probably because the charge repulsion at the N-terminal Pro weakens the H-bonding more than the fraying Gly at the C-terminus. Our findings also suggest that CMPs featuring Ac–(G)POG⋯GPO–NH_2_ as the ending motifs are more likely to have reduced terminal flexibility and may be more suitable for future crystallographic or NMR studies.

Conventionally, Gly was preferred as the C-terminal residue in many collagen peptide studies probably because of the affordability of Gly-preloaded resins and the reduced risks of epimerization owning to its lack of chirality. For decades, (POG)_*n*_ and (GPO)_*n*_ have been considered interchangeable.^28,35,58,59^ Our study disproves this assumption and points out the need to note the terminal repeats when comparing CMPs from different works. The role of the common terminal functional groups in the triple-helix stability of CMP was recently highlighted^27^ and incorporated into an algorithm for predicting the stability of collagen triple-helices.^21^ Our findings show that the reported effects of the terminal functional groups only apply to the POG end motif,^27^ but not the terminal OGP- and GPO-repeats (Fig. 6b and c). Our study emphasizes the need and provides the reference to account for the difference in terminal repeats in such algorithms to avoid unexpected biases.^21^

Our work showed that the terminal repeats affect not only the assembly of CMP homo-trimers but also how strongly the peptide strands form hybrid triple-helices with natural collagen chains (Fig. 6d and e). For applications, this study will provide helpful guidance in designing potent collagen targeting probes^47,48^ and fabricating synthetic collagen materials.^29,30^ Meanwhile, similar investigations of terminal repeats have been rare for fibrous structural proteins, such as keratin, silk fibroin, elastin, fibrin, and myosin, many of which are insoluble and lack in crystallography based structural elucidation. Our findings and methods may inspire new investigations into the folding of these repeat proteins, particularly for the sequence–structure relationship at their termini.

## Data availability

The data that support the findings of this study are available within the article and its ESI,† or from the corresponding author on reasonable request.

## Author contributions

Y. Q., G. L. and Y. L. conceived and designed the experiments. Y. Q. and D. Z. performed the experiments and carried out the data acquisition. Z. Q. conducted molecular dynamics simulations. All authors analyzed and interpreted the data. Y. Q., D. Z., G. L., Z. Q. and Y. L. prepared the manuscript with feedback from the other authors. All authors have given approval to the final version of the manuscript.

## Conflicts of interest

The authors declare no competing interests.

## Supplementary Material

SC-013-D2SC03666E-s001
