# Supplementary material for: Terminal repeats impact collagen triple-helix stability through hydrogen bonding
Source: Chem Sci. 2022 Oct 20;13(42):12567–76. doi: 10.1039/d2sc03666e (PMC9629113; doi:10.1039/d2sc03666e)
Supplement: SC-013-D2SC03666E-s001 [file SC-013-D2SC03666E-s001.pdf]

Electronic Supplementary Material (ESI) for Chemical Science.
This journal is © The Royal Society of Chemistry 2022

Terminal Repeats Impact Collagen Triple-Helix Stability through Hydrogen Bonding

Yingying Qi[†,‡,§], Daoning Zhou[†], Julian L. Kessler[^], Rongmao Qiu[†], S. Michael Yu[^], Gang Li[‡,*], Zhao Qin[‖,*], and Yang Li[†,*]

[†]Guangdong Provincial Key Laboratory of Biomedical Imaging and Guangdong Provincial Engineering Research Center of Molecular Imaging, The Fifth Affiliated Hospital, Sun Yat-sen University, Zhuhai 519000, China
[‡]Cardiac Surgery and Structural Heart Disease Unit of Cardiovascular Center, The Fifth Affiliated Hospital, Sun Yat-sen University, Zhuhai 519000, China
[§]Department of Radiology, The Fifth Affiliated Hospital, Sun Yat-sen University, Zhuhai 519000, China
[^]Department of Biomedical Engineering, University of Utah, Salt Lake City, Utah 84112, USA
[‖]Department of Civil & Environmental Engineering, College of Engineering & Computer Science, Syracuse University, Syracuse, New York 13244, USA

*Correspondence to:
Gang Li: gangli73@163.com; Zhao Qin: zqin02@syr.edu; Yang Li: liyang266@mail.sysu.edu.cn

Table of Contents

	Page
**Section 1: Materials and Methods**	2
Materials	2
Solid-phase peptide synthesis	3
Cleavage protocols	3
Purification and mass spectrometry	4
Circular dichroism spectroscopy	4
B-factor analysis	5
Fully atomistic molecular dynamics of collagen sequences	5
Post analysis of simulation results	6
Differential scanning calorimetry	6
Gelatin binding assay	7
Immunofluorescence staining	7
**Section 2: Supplementary Tables and Figures**	8
Table S1. Terminal sequences of human collagen triple-helix regions.	8
Table S2. Sequences of all CMPs and their T_m values.	9
Table S3. Crystal structure analysis of CMP triple-helices in PDB.	10
Table S4. Comparison between various T_m values of similar CMPs.	11
Figure S1. T_m values of Ac-(GPO)$_7$-NH$_2$ under different CD heating rates.	12
Figure S2. The T_m values of (POG)$_n$ and (GPO)$_n$ (n = 5~9).	13
Figure S3-4. Normalized B-factors of CMP peptide chains in PDB.	14-15
Figure S5. The B-factor deviation at N-terminus of CMP triple-helices in PDB.	16
Figure S6-7. Molecular dynamics simulation of CMP **1**, **2**, **3**, and **2E**.	17-18
Figure S8. Experimental curves of the DSC.	19
Figure S9-10. T_m value changes with stepwise residue incorporations at N- and C-termini.	20-21
Figure S11. CMP and anti-collagen I antibody staining of rat heart tissue.	22
Figure S12. Φ, ψ angles of representitive residues in simulated structures of CMP **2** and **3**.	23
**Section 3: MALDI and CD characterization of all CMPs**	24-52
**References**	52

Section 1: Materials and Methods

Materials

Commercially available materials and reagents were used without further purification. The solvents, resin, Fmoc amino acids, and reagents used in the synthesis and purification of the peptides were purchased from the following suppliers.

General reagent	Supplier	Catalog number
N-Methylpyrrolidinone (NMP)	Aladdin	M100588-2.5L
N,*N*-Dimethylformamide (DMF)	Aladdin	D112002-500ml
Dimethyl sulfoxide (DMSO)	Aladdin	D106264-50g
Methylene Chloride (DCM)	Aladdin	D116146-4L
Acetonitrile	Macklin	A800362-4L
Ethyl ether	Guangzhou chemical reagent factory	20201205 10
TentaGel RAM resin	Peptides International	RTS-9995-PI
Fmoc-Pro-Wang resin	Aladdin	P118261-5g
Fmoc-Gly-Wang resin	Aladdin	G116094-5g
Fmoc-Hyp-Wang resin	GL Biochem	41506
Fmoc-Gly-OH	EMD Millipore	852001
Fmoc-Pro-OH	EMD Millipore	852017
Fmoc-Hyp(tBu)-OH	EMD Millipore	852036
Fmoc-Sar-OH	Macklin	F809960-25g
Methyl iodide	Anaiji	W6107051000-A01
Piperidine	Detian Fine Chemicals	20191105
O-(7-Azabenzotriazol-1-yl)-*N*,*N*,*N*',*N*'-tetramethyluronium hexafluorophosphate (HATU)	Aladdin	H109327-100g
1-Hydroxy-7-azabenzotriazole (HOAt)	Macklin	H811122-100g
(7-Azabenzotriazol-1-yloxy) trispyrrolidinophosphonium hexafluorophosphate (PyAOP)	GL Biochem	GLS190801-00809
N-Ethyldiisopropylamine (DIPEA)	EMD Millipore	S1807494945
Acetic acid	Aladdin	A116174-500ml
Trifluoroacetic acid (TFA)	Macklin	T818782-500ml
Triisopropylsilane (TIS)	Macklin	T819181-100ml
5(6)-Carboxyfluorescein	Macklin	C804899-1g
Anhydrous potassium carbonate	Aladdin	P111561-100g

Solid-phase peptide synthesis

Peptides were prepared using TentaGel R RAM resin (substitution level: 0.19 mmol/g) to give C-terminal amidation *via* solid-phase synthesis by manual or automated peptide synthesizer (Gyros Protein Technologies, PurePep Chorus). For manual synthesis, each peptide was typically synthesized on 50 mg of resin (containing 10 μmol of reaction sites) swollen in 0.4 mL of DMF for over 1 hour before deprotection and the first coupling step. For coupling, the resin was agitated with a solution of Fmoc amino acids (50 μmol), HATU (50 μmol, 19 mg), HOAt (50 μmol, 6.8 mg), and DIPEA (75 μmol, 13 μL) in 0.4 mL DMF over a three-hour period. The Fmoc protection group was removed by incubating the resin in DMF for 20 minutes with 20% v/v piperidine. The resin was drained and cleaned after each reaction. Following each reaction, the resin was drained and washed with DMF (3×10 mL). The N-terminal amine of each sequence was acetylated by combining the resin (5 μmol) with a solution of acetic acid (50 μmol, 3 μL), HOAt (50 μmol, 6.8 mg), HATU (50 μmol, 19 mg), and DIPEA (75 μmol, 13 μL) in 0.5 mL of DMF at room temperature for 3 hours after Fmoc-deprotection of the final amino residue. The standard Kaiser and chloranil resin tests were used to monitor all coupling and deprotection reactions. The chemical reagents and the ratios used for the automated systhesis were the same as the manual synthesis. Each amino acid was double coupled under heating at 50 °C for 5 min and the Fmoc protection group was removed with two rounds of 90 s treatment of 20% piperidine.

Fmoc-Gly-Wang resin (substitution level: 0.40 mmol/g) pre-coupled with Gly was used to produce the C-terminal carboxyl peptide Ac-$(POG)_7$-OH. Ac-$(GPO)_7$-OH and Ac-$(OGP)_7$-OH were purchased from GL Biochem (Shanghai). The peptide containing the C-terminal methyl ester was prepared by reacting the C-terminal carboxyl peptide Ac-$(GPO)_7$-OH (2.5 μmol, 5 mg) with potassium carbonate (7.8 μmol, 1.1 mg) and methyl iodide (5.2 μmol, 0.32 μL) in 1 mL DMF. The reaction mixture was stirred for 24 hours at room temperature. After the reaction, insoluble potassium carbonate was removed by filtration and the peptide product was purified by HPLC.

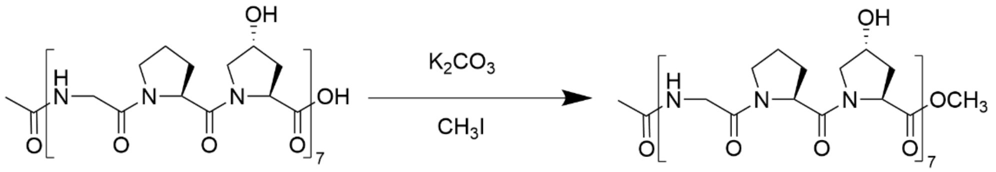

Cleavage protocols

Before cleavage of the polypeptide, the resin was washed and drained with DMF (3×10 mL) and DCM (5×10 mL). Typically, RAM resin (5 μmol) was treated with 1 mL mixture of TFA/TIS/water (95:2.5:2.5) with stirring for over 3 hours. After that, the resin was rinsed with 0.5 mL of TFA. The TFA cleavage solution was collected and evaporated under a stream of nitrogen down to roughly 0.5 mL after the resin was filtered. Then the crude peptide was precipitated in 5 mL of cold diethyl ether. The crude product was rinsed twice with cold diethyl ether after

centrifugation. Wang resin was treated the same as RAM resin, except that the cleavage fluid was a mixture of TFA and DCM (50:50).

Purification and mass spectrometry

Using a mixture of water (A) and acetonitrile (B) containing 0.1% TFA, all peptides were purified using reverse-phase high performance liquid chromatography (HPLC) on a semi-preparative column (Agilent ZORBAX StableBond 300 C18) at a linear gradient: 5 % to 45 % acetonitrile for 30 min. For semi-preparative HPLC, flow rates of 4 mL/min were employed. The pure fractions were collected and lyophilized.

**Time (min)**	0	5	25	25.5	30
**B%**	5%	5%	45%	5%	5%

Mass spectrometry was used to verify all purified peptides. A Shimadzu 8020 or a Bruker Reflex III matrix aided laser analytical ionization-time of flight (MALDI/TOF) mass spectrometer were used to collect mass spectra. The lyophilized products were dissolved in Milli-Q water as stock solutions. The UV absorbance of the CMP solutions was measured at 214 nm for unlabeled peptides (extinction coefficient: 2200 $M^{-1}cm^{-1}$ per peptide bond) and 492 nm for the carboxyfluorescein-tagged CMPs (extinction coefficient: 70000 $M^{-1}cm^{-1}$ per tag) on a IMPLEN NP80 Nano drop using a quartz cell with a 1 cm cell path length.[1]

Circular dichroism (CD) spectroscopy

CMP stock solutions were diluted to 150 µM in 1×PBS and heated at 80 °C for 10 min before incubation at 4 °C for over 48 hours to allow the peptide strands to fully assemble into the triple-helix. Peptides containing an N-terminal amino group were also diluted in NaOH solutions (3.5 mM, pH 11.5) to a final peptide concentration of 150 µM. On a JASCO J-1500 CD spectrophotometer, CD measurements of the peptide solutions were taken in quartz cells with a path length of 0.1 cm.

The CD spectra were scanned with the parameters (scanning speed: 20 nm/min; data pitch: 0.1 nm; bandwidth: 5 nm; digital integration time: 16 s). The thermal denaturation curves were obtained by monitoring the ellipticity at 225 nm with a heating rate of 0.5 °C/min from 4 to 80 °C. Using the JASCO Spectra Manager software (Version 2.10.05), a melting curve's derivative was constructed, and the temperature at the lowest of the derivative curve was defined as the melting temperature (T_m). For all collagen mimetic peptides, the difference between the two recorded T_m values was less than 1 °C, and each T_m value presented in this work was averaged from two groups of parallel experiments. The mean residue ellipticity (MRE, [θ]) was determined using the equation $[\theta]=(\theta\times m)/(c\times l\times n)$ [θ: measured ellipticity (mdeg); n: the number of amino acid residues in the peptide; m: the molecular weight (g/mol); c: the concentration (mg/mL); l: the path length of the cuvette (mm)].

B-factors calculations

To allow comparison among different PDB structures, the normlized B-factors (Figure S4-5) of each CMP triple-helix structure we found in PDB (Figure S2) were calculated by dividing the B-factor of each non-hydrogen atom by the mean B-factor value of all non-hydrogen, non-water atoms within the whole triple-helix. To further reflect the deviation of the B-factors at the N- and C-termini of each peptide chain, we assessed the standard deviation (designated as σ) of the normalized atomic B-factors of the N-terminal most (Figure S3) and the C-terminal most amino acid triplet (Figure 3b) using the equation:

$$\sigma = \sqrt{\frac{1}{N}\sum_{i=1}^{N}(B_i - 1)^2}$$

where N is the number of totally non-hydrogen peptide atoms in the terminal unit of interest, B_i is the value of the normalized B-factor of a given non-hydrogen atom within the terminal unit, and 1 represents the nomalized mean B-factor value of the whole triple-helix.

Fully atomistic molecular dynamics (MD) of collagen sequences

We started by using the fully equilibrated tropocollagen structure of $(GPO)_{10}$ as the template, we used psfgen, as a tool of NAMD package[2] to remove certain amount of residues from the N- and C-terminus, and added acetylated N-terminus (Ac-) and amidated C-terminus ($-NH_2$) and methylated C-terminus ($-OCH_3$) to each of the collagen chains to obtain the initial molecular structure corresponding to the sequence Ac-$(GPO)_7$-NH_2, Ac-$(GPO)_7$G-NH_2, Ac-$(POG)_7$-NH_2, Ac-$(GPO)_7$-OCH_3 as in this study. Classical fully atomistic molecular dynamics (MD) simulations were used to fully equilibrate the collagen in the explicit water box with dimension of 10.5×5×5 nm^3 composed of 100 mM NaCl and TIP3P water molecules[3]. Simulation was carried with a CHARMM27 all atom energy force field[3]. The net charge of the system was zero by adjusting the ratio between cation and anion and each ion was initially distributed randomly in water with at least 5 Å from the collagen structure. The simulation time step was 2 fs with rigid bonds model for all the covalent bonds between hydrogen atoms and other heavy atoms. We use particle mesh Ewald (PME) function with a grid width <1 Å to calculate the electronic interaction as an efficient method to accurately include all the long-distance electrostatics interactions. For the initial run, each of all the backbone atoms was constrained in space by elastic springs with the stiffness of 5 kcal/mol/$Å^2$ in all the 3 directions, while the side chain atoms and solvent molecules were free to move. We ran 10,000 steps of energy minimization by using a conjugate gradient and line search algorithm followed by 2 ns dynamics run for initial equilibrium. The dynamics simulation was performed in the NPT (constant pressure of 1 atm and constant temperature of 310 K) ensemble controlled by the Langevin dynamics to reach a constant pressure and temperature (with 5 ps^{-1} damping coefficient for temperature, 100 fs oscillation period and 50 fs damping time scale for pressure control) with the shrinkable volume during the run. After the initial run, we restarted the simulation, released all the constraints on the backbone

atoms and continue using NPT ensemble for 20 ns in the purpose of getting fully equilibrated collagen structures. The simulations were performed by using a NAMD package (v2.12)[2] in the local Linux workstation with multiple graphics processing units (NVIDIA RTX 3060) support through Compute Unified Device Architecture (CUDA v11.4).

Post analysis of simulation results

Visual Molecular Dynamics (VMD) was used for post-processing of the MD simulation results. It was used to visualize and analyze molecular structures by going through each frame of the simulation trajectory (2 ps per frame, 10,000 frames in total) that include the double-precision coordinate of each atom. We developed our TCL scripts in VMD to calculate the number of hydrogen bonds between any two residues I and J (given by H_{IJ}, as illustrated by each pixel in Fig. 4a). By going through all the atom pairs eligible to be donor/acceptor, a hydrogen bond was added to H_{IJ} if the donor-acceptor distance is smaller than 3.5 Å and the donor-hydrogen-acceptor angle is smaller than 35º. We computed the time-average value of this matrix $\overline{H_{IJ}}$ and plot it by heat maps (Fig. 4a by using MATLAB. We removed the overall rigid-body movement of the collagen by aligning all the frames against the geometry of the collagen after the initial run. To understand the effect of collagen sequence on the dynamics of the molecule at different regions, we focused on three regions including 9 residues at the N-terminal (residue 1–3 for each of the three chains), 9 residues at middle (residue 10–12 for each of the three chains) and 9 residues at C-terminal (residue 20–22 for each of the three chains in CMP 3, residue 19–21 in other sequences). We computed the root-mean-square deviation (RMSD) and the radius of gyration (R_g) for each of these three regions in each frame (every 2 ps during the simulation, raw data as plotted in Fig. S6 and Fig. S7). We computed the time-average and standard deviation value of RMSD and R_g during the 20 ns relaxation process, as shown by the bar plots in Fig. 4b. We ran two-sample t-test for RMSD and R_g between Ac-(GPO)$_7$-NH_2 and other sequences. Since t-test on large sample size almost always lead to an extremely small p-value once the mean was slightly different, we chose to compute the mean value of RMSD and R_g for every 200 ps, and use N=100 per group to perform the t-test and compute the *p*-value, as given by the stars in Fig.4b.

Differential scanning calorimetry (DSC)

All DSC studies were carried out using a MicroCal VP-DS Micro Calorimeter with the parameters (heating range: 20-70 °C, scan rate 2 °C/min, feedback set to "none" for enhanced signal-to-noise ratio).[4,5] All CMP samples were heated at 85 °C for 10 min and incubated at 4 °C for at least 48 hours before measurement to allow full triple-helix assembly. Each peptide solution (250 μM in PBS) were centrifuged at 12,000 rpm for 10 minutes at 4°C before testing, and the supernatant was added to the sample cell. Measurements were repeated three times on three identical samples and averaged for each peptide. For data analysis, the DSC curve in PBS buffer was used as the baseline and subtracted from each peptide curve. The heat capacity (*C*p) baseline was also subtracted. The melting

temperature of the system was defined as the temperature at which the maximum measured *C*p was observed. The enthalpy change (ΔH) for this process was measured as the calorimetric area under each DSC unfolding curve. The non-two state fitting method was used to fit the data.

Gelatin binding assays

Carboxyfluorescein-labeled CMP **1F** [CF-Ahx-$(POG)_7$-NH_2], CMP **2F** [CF-Ahx-$(GPO)_7$-NH_2] and CMP **4F** [CF-Ahx-$(OGP)_7$-NH_2] were prepared by reacting the N-terminal amines of the sequences on-resin with 6 molar equivalents of 5(6)-carboxyfluorescein activated by 6 molar equivalents of PyAOP with 12 molar equivalents of DIEA in NMP for over 48 hours before the resins were treated with 20% piperidine (in DMF) for 30 min. The CF-CMPs were cleaved from the resin, purified by HPLC with protection from ambient light, and verified by MALDI-MS (calculated mass: 2381.0 $[M+Na]^+$ for the three CMPs; CMP **2F**, observed: 2380.0 $[M+Na]^+$; CMP **1F**, observed: 2379.9 $[M+Na]^+$; CMP **4F**, observed: 2381.0 $[M+Na]^+$).

The wells of a 96-well plate (Nunc, 468667-1) were coated with 50 µL of gelatin solution (100 µg/mL, Sigma-Aldrich, V900863-100G) in 1×PBS and incubated at 4 °C overnight. The wells were rinsed with 250 µL of 25 °C 1×PBS six times. PBS solution (100 µL) containing 15 µM of CMP **1F**, **2F** or **4F** was heated at 80 °C for 10 min and cooled in ice-water bath for 15 s before being immediately added to each well to allow binding with the coated gelatin for 4 hours at 4 °C. After washing 6 times with 1×PBS, the fluorescence intensity of each well was measured on a Synergy HTX multi-mode reader (Ex: 485 nm, Em: 528 nm, Gain: 80).

Immunofluorescence staining

Paraffin-embedded sections of hearts from normal SD rats (purchased from UptBio) were deparaffinized, washed with PBS and blocked with 5% goat serum according to standard procedures.[7] Antigen-retrieval process were performed by heating the slides at 110 °C in sodium citrate buffer in a pressure cooker for 10 min. PBS solution (100 µL) containing 15 µM of CMP **1F**, **2F** or **4F** was heated at 85 °C for 10 min and cooled in ice-water bath for 15 s before being immediately added to the tissue slides. The slides were incubated with the CMP solutions at 4 °C overnight.[8] After staining with DAPI followed by three rounds of PBS wash, the slides were mounted for imaging (EVOS M7000 imaging system, Thermo Fisher). In another experiment, 85 °C-heated CMP **2F** solution (15 µM in PBS) was immediatedly cooled on ice for 15 s before an anti-collagen I antibody (Abcam ab24821; 1:300) was diluted into it. The slides were co-stained by this mixture of CMP **2F** and the collagen I antibody as described above. The primary antibody was detected with a AF647-labeled secondary antibody (Abcam ab150079; 1:200), washed thoroughly with PBS, and mounted for imaging.

Section 2: Supplementary Tables and Figures

Table S1. Terminal (boundary) sequences of the recognized triple-helix regions in different types of human collagen α-chains in UniProt. All terminal sequences featuring non-GXY repeats were shaded in grey.
*Multiple terminal repeats were shown for chains with multiple triple-helix regions.

Human collagen type & chain		UniProt ID	Sequence of the triple-helix domain	
			N-terminal most 9 amino acids	C-terminal most 9 amino acids
I	α1	P02452	GPMGPSGPR	GPPGPPGPP
II	α1	P02458	GPMGPMGPR	GPPGPPGPP
III	α1	P02461	GLAGYPGPA	GPPGAPGPC
IV	α1	P02462	GERGFPGIP	PPGPDGLPG
	α2	P08572	GEPGEPGLV	PGLPGMPGR
	α3	Q01955	GAKGEKGEK	GKRGDSGSP
	α4	P53420	GPPGPPGPQ	GDPGPKGFG
V	α1	P20908	GPAGPMGLT	PPGPPGPPG
	α3	P25940	GPPGAPGPQ	GPPGPPGAP
VI	α1	P12109	GPPGLRGDP	GHQGPPGPD
	α2	P12110	PSGPKGYRG	GPPGDPGLT
	α3	P12111	QRGDRGPIG	KGNRGDSID
VII	α1	Q02388	GQKGEPGEM	PRGEKGEAA
VIII	α1	P27658	GEQGPRGEP	GPPGPPGPP
	α2	P25067	GEPGPPGKP	GPPGAPGAF
IX	α1	P20849	GATGLPGRP	GPPGLPGFC
	α2	Q14055*	GKDGDRGSP / GAVGMMGPP / GMKGPPGLQ / GPPGERGPP	GPVGLPGFC / GLPGRPGQA / GRQGVEGRDGKPGRPGTI
	α3	Q14050*	GQDGAPGEP / GSIGRPGPA / GLPGPPGPP	IGAQGTPGI / GPQGVPGTS/ GKPGVPGKE
X	α1	Q03692	GEQGTPGPP	GPPGPPGQA
XI	α1	P12107	GPPGPMGLT	GSPGPPGEV
	α2	P13942	GPPGPMGYT	GHPGPPGEV
XII	α1	Q99715*	GPPGPPGPP / GPPGPPGPA	GPPGPPGYC / GEKGDRGDI
XIII	α1	Q5TAT6*	GPPGPTGRP / GPPGPPGPP / GPPGLPGQI	GEKGQCGEY / GSKGEPGKG / GDQGAPGLD
XIV	α1	Q05707*	VALGPAGPP / GPPGEPGRP	GERGERGDL / APHPDQPEF
XV	α1	P39059*	GVKGEKGDR / GPPGEKGSW / GLKGLPGNP	GAQGPPGPP / GPPGPPGPP / GPRGPPGHI
XVII	α1	Q9UMD9	GSPGPKGDM	GEKGDKGDQ
XVIII	α1	P39060*	GPPGPPGPP / GEKGDRGDA	GPPGPPGPPGPP / ERGEPGGGG
XIX	α1	Q14993*	GPPGPKGEK / GRPGPPGKD / QASVPGLKS	GPPGPPGPP / GLPGSPGAP / GPPGPPGIP
XXVII	α1	Q8IZC6	GPPGPKGDC	GRPGPPGPP

Table S2. The sequences and T_m values of all CMPs in this study (O: hydroxyproline, Ac: acetyl, NH_2: amide, OH: carboxyl, OCH_3: methyl ester, Sar: sarcosine).

Acronym in main text	Sequence	T_m (°C) in PBS
	Ac-$(POG)_5$-NH_2	--
	Ac-$(POG)_6$-NH_2	30
CMP **1**	Ac-$(POG)_7$-NH_2	45
	H-$(POG)_7$-NH_2	37
	Ac-$(POG)_7$-OH	42
	Ac-$(POG)_8$-NH_2	56
	Ac-$(POG)_9$-NH_2	65
	Ac-$(GPO)_5$-NH_2	23
	Ac-$(GPO)_6$-NH_2	42
CMP **2**	Ac-$(GPO)_7$-NH_2	55
	H-$(GPO)_7$-NH_2	49
CMP **2O**	Ac-$(GPO)_7$-OH	36
CMP **2E**	Ac-$(GPO)_7$-OCH_3	44
	Ac-$(GPO)_8$-NH_2	64
	Ac-$(GPO)_9$-NH_2	72
CMP **3**	Ac-G$(POG)_7$-NH_2 = Ac-$(GPO)_7$G-NH_2	47
CMP **3A**	H-G$(POG)_7$-NH_2 = H-$(GPO)_7$G-NH_2	42
CMP **3S$_N$**	Ac-Sar$(POG)_7$-NH_2	41
CMP **3S$_C$**	Ac-$(GPO)_7$Sar-NH_2	42
CMP **4**	Ac-$(OGP)_7$-NH_2	44
	H-$(OGP)_7$-NH_2	43
	Ac-$(OGP)_7$-OH	36
	Ac-$(OGP)_8$-NH_2	56
CMP **5**	Ac-P$(OGP)_7$-NH_2 = Ac-$(POG)_7$P-NH_2	52
CMP **6**	Ac-O$(GPO)_7$-NH_2 = Ac-$(OGP)_7$O-NH_2	56
	Ac-GP$(OGP)_7$-NH_2 = Ac-$(GPO)_7$GP-NH_2	54
	Ac-PO$(GPO)_7$-NH_2 = Ac-$(POG)_7$PO-NH_2	62
	Ac-OG$(POG)_7$-NH_2 = Ac-$(OGP)_7$OG-NH_2	48

Table S3. Analysis crystal structure of reported CMP triple-helices in PDB. Note that more unresolved or missing terminal residues are found in the POG-repeating sequences.

PDB ID	N-end group	Sequence	C-end group	Unresolved terminal residues		Distance & angle for the possible C-end most (Pro) C=O…NH(Gly/H) H-bonds
				**N-end**	**C-end**	
5K86	H	$(POG)_3PRX_{za}(POG)_4$	NH_2	-	-	2.8 (172.9°)<br>2.8 (159.7°)
4Z1R	H	$(POG)_4POA(POG)_5$	NH_2	B chain: Pro & Hyp;<br>C chain: Pro & Hyp	A chain: Pro, Hyp&Gly;<br>B chain: Hyp & Gly;<br>C chain: Pro, Hyp&Gly	2.9 (156.3°)
3WN8	H	$(POG)_3PRG(POG)_4$	OH	-	B chain: Gly	2.8 (153.5°)<br>2.8 (173.9°)
2DRX	H	$(POG)_4LOGLOG(POG)_4$	OH	B chain: Pro	A chain: Hyp & Gly;	3.2 (160.0°)<br>3.0 (142.6°)
2DRT	H	$(POG)_4LOG(POG)_5$	OH	A chain: Pro;<br>B chain: Pro	C chain: Hyp & Gly	2.6 (161.3°)<br>2.6 (149.5°)
1CAG	H	$(POG)_4POA(POG)_5$	OH	-	A chain: Gly;<br>C chain: Gly	2.5 (122.7°)
1BKV	H	$(POG)_3ITGARGLAG(POG)_4$	OH	A chain: Pro	-	2.9 (153.8°)
1CGD	H	$(POG)_4POA(POG)_5$	OH	-	-	3.1 (174.3)<br>2.6 (160.9°)
3POD	Ac	$(GPO)_3GKL(GPO)_4$	NH_2	-	-	2.7 (159.5°)<br>2.8 (160.6°)<br>2.9 (165.8°)
3PON	Ac	$(GPO)_4GKL(GPO)_4$	NH_2	-	C chain: Pro & Hyp	2.9 (162.3°)<br>3.0 (161.3°)
7JX4	Ac	$(GPO)_3$G-NLys-O$(GPO)_3$	NH_2	-	-	2.8 (95.1°)<br>2.9 (86.1°)<br>2.9 (162.8°)
7JX5	Ac	$(GPO)_3$G-NPhe-O$(GPO)_3$	NH_2	-	-	-
1Q7D	Ac	$(GPO)_2GFOGER(GPO)_3$	NH_2	-	C chain: Hyp	2.7 (160.3)<br>2.7 (146.2)
4DMT	Ac	$(GPO)_3GPRGQOGVMGFO(GPO)_3$	NH_2	A chain: Ac	-	2.9 (163.1°)<br>2.9 (153.7°)<br>2.8 (165.3)
3P46	Ac	$(GPO)_2GLOGEA(GPO)_2$	NH_2	C chain: Ac	-	3.0 (144.3°)<br>2.6 (160.2°)

Table S4. Comparison between various T_m values of similar CMPs from this study and previous reports.

**CMP**	**Source**	**T_m (°C)**	**N-end**	**Sequence**	**C-end**	**Heating rate (°C/min)**	**Notes**
$(POG)_8$	This work	56	Ac-	$(POG)_8$	$-NH_2$	0.5	The decreased T_m value compared to our work is most likely due to the slower heating rate.
	Ref. 25	50.5	Ac-	$(POG)_8$	$-NH_2$	0.1	
	T_m estimation considering each factor	50	0	0	0	-6	
			estimated ΔT_m (°C) comparing to our work				
$(POG)_{10}$	This work	65	Ac-	$(POG)_9$	$-NH_2$	0.5	The decreased T_m value compared to our work is likely due to the slower heating rate and charge repulsions between the uncapped C- and N-termini.
	Ref.56 *Biopolymers*. 2011;96(1):4-13.	57	NH_2-	$(POG)_{10}$	-COOH	0.1	
	T_m estimation considering each factor	56	-8	+8	-3	-6	
			estimated ΔT_m (°C) comparing to our work				
$(GPO)_8$	This work	64	Ac-	$(GPO)_8$	$-NH_2$	0.5	Besides the factor of the slower CD heating rates, the decrease in T_m value comparing to our study is probably due to the influence of C-terminal GlyGly which should disrupt the terminal H-bonding, according to our study.
	Ref. 19	47.3	Ac-	$(GPO)_8$GG	$-NH_2$	0.1	
	Ref. 26	44.5	Ac-	$(GPO)_8$GG	$-NH_2$	< 0.1	
	T_m estimation considering each factor	< 50	0	< -8	0	-6	
			estimated ΔT_m (°C) comparing to our work				

a

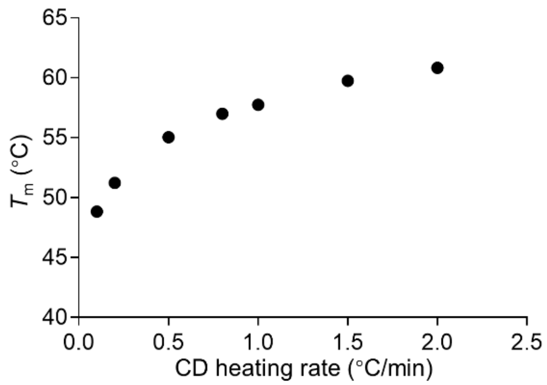

b

CD heating rate (°C/min)	T_m (°C)
0.1	49
0.2	51
0.5	55
0.8	57
1	58
1.5	60
2	61

Figure S1. The T_m values of Ac-$(GPO)_7$-NH_2 measured under various heating rates (peptide concentration: 150 μM; buffer: PBS; CD wavelength: 225 nm; data interval: 0.1 °C; bandwidth: 5 nm; digital integration time: 16 s, temperature range: 4 to 80 °C).

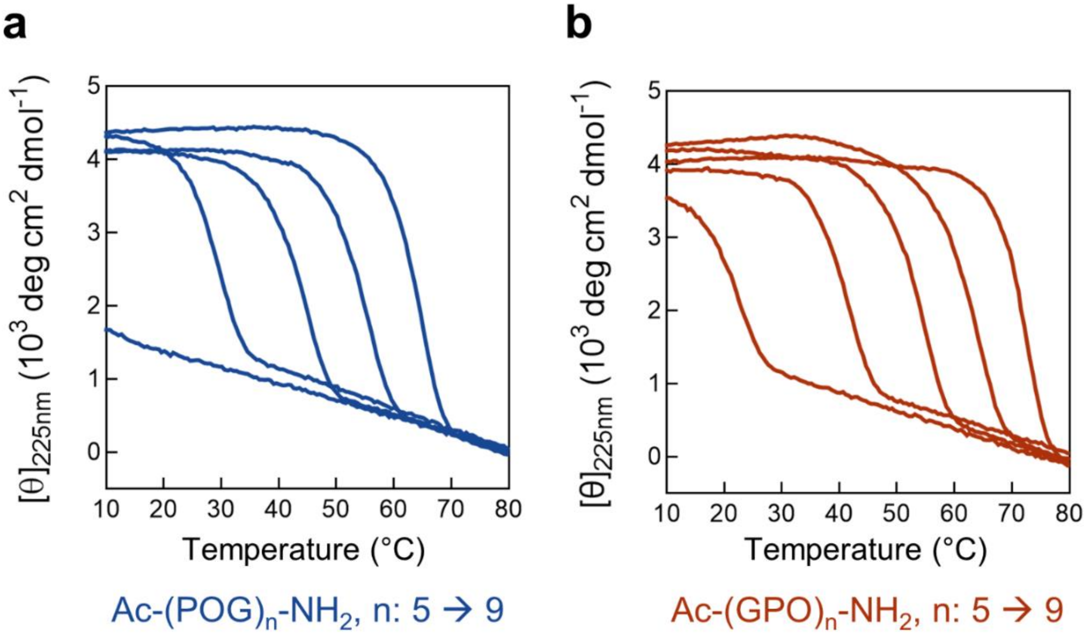

c

n =	T_m (°C) Ac-(POG)$_n$-NH$_2$	T_m (°C) Ac-(GPO)$_n$-NH$_2$	ΔT_m
5	--	23	--
6	30	42	12
7	45	55	10
8	56	64	8
9	65	72	7

Figure S2. The T_m values of GPO-repeating CMPs are consistently higher than the POG-ones of equal numbers of repeats. **a,** Thermal denaturation curves of Ac-(POG)$_n$-NH$_2$ with n varying from 5 to 9. Ac-(POG)$_5$-NH$_2$ does not form a triple-helix. **b,** Thermal denaturation curves of Ac-(GPO)$_n$-NH$_2$ with n varying from 5 to 9. **c,** T_m values of the two CMP series with incremental numbers of repeats and the gradually decreasing ΔT_m value.

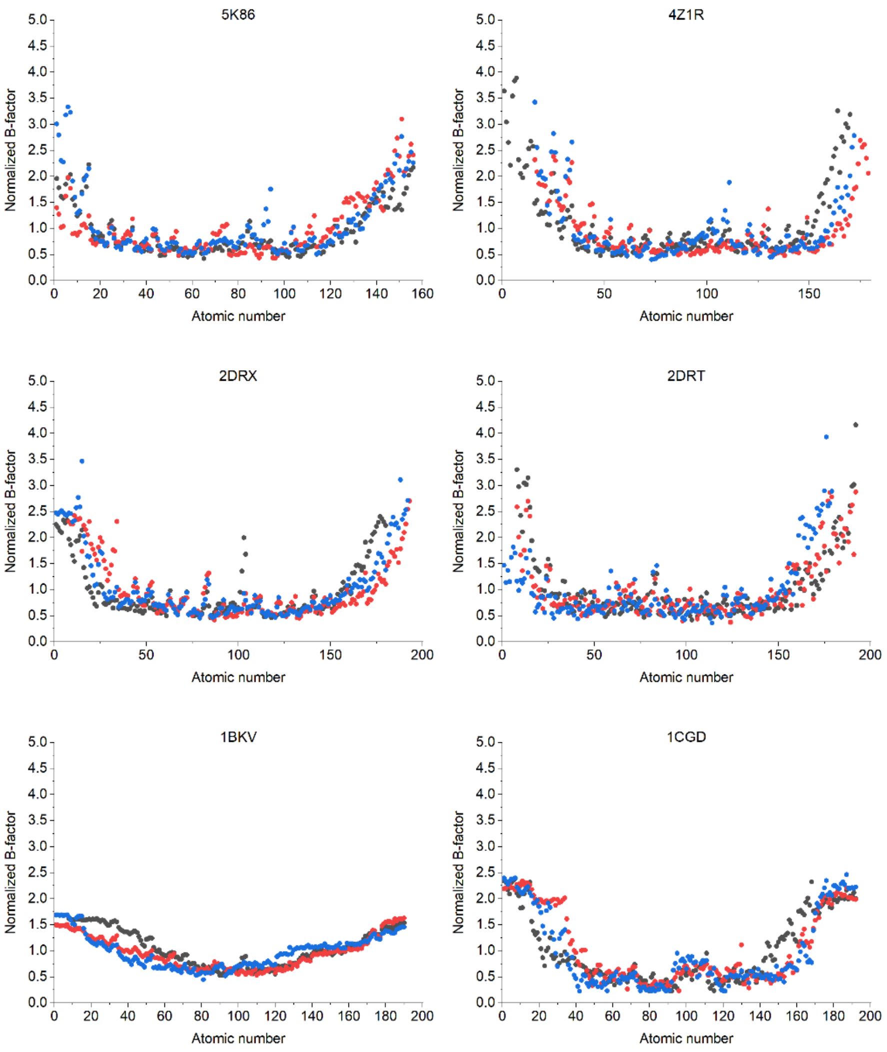

Figure S3. Normalized B-factors for all non-hydrogen atoms of CMPs featuring the POG repeats.

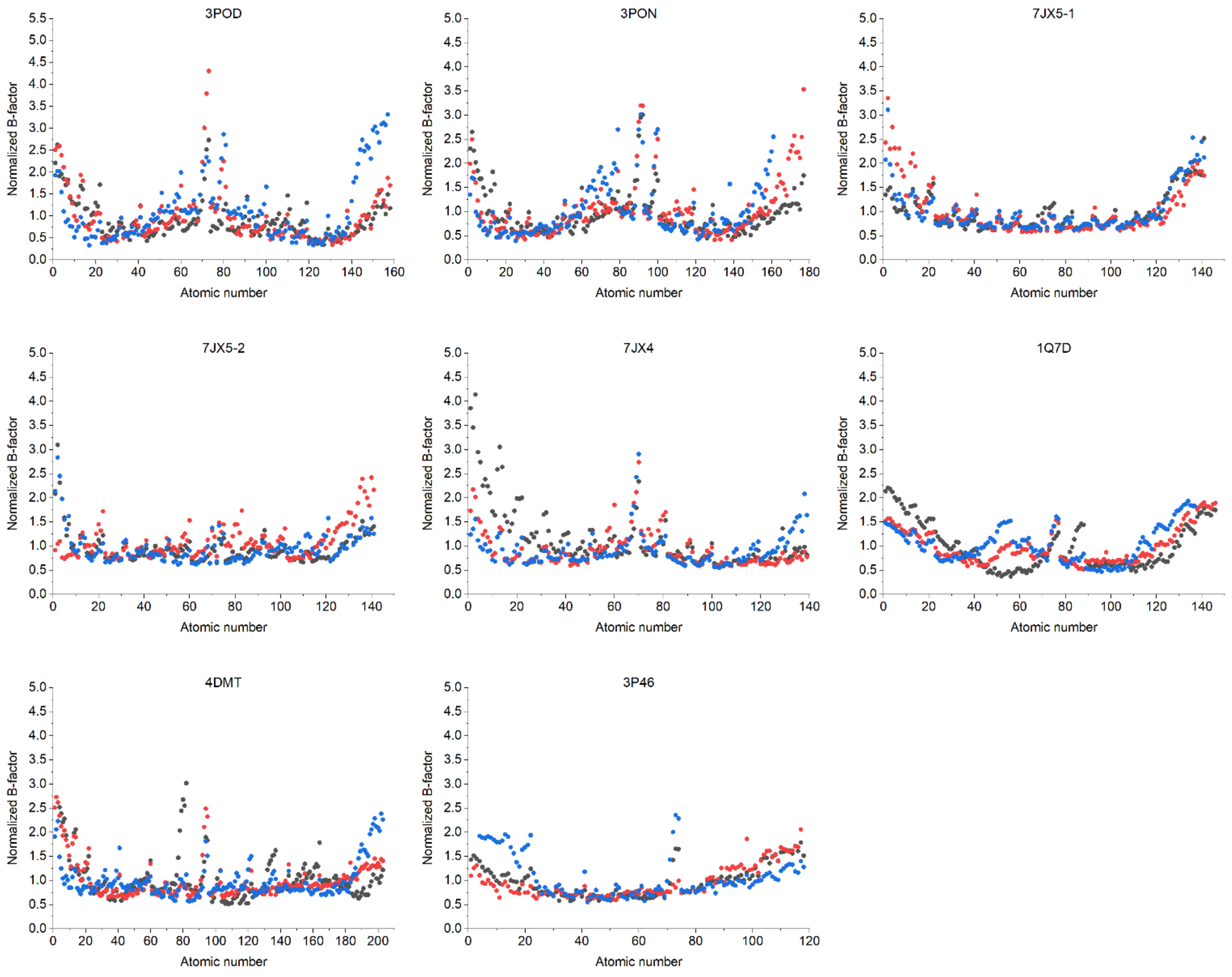

Figure S4. Normalized B-factors for all non-hydrogen atoms of CMPs featuring GPO repeats.

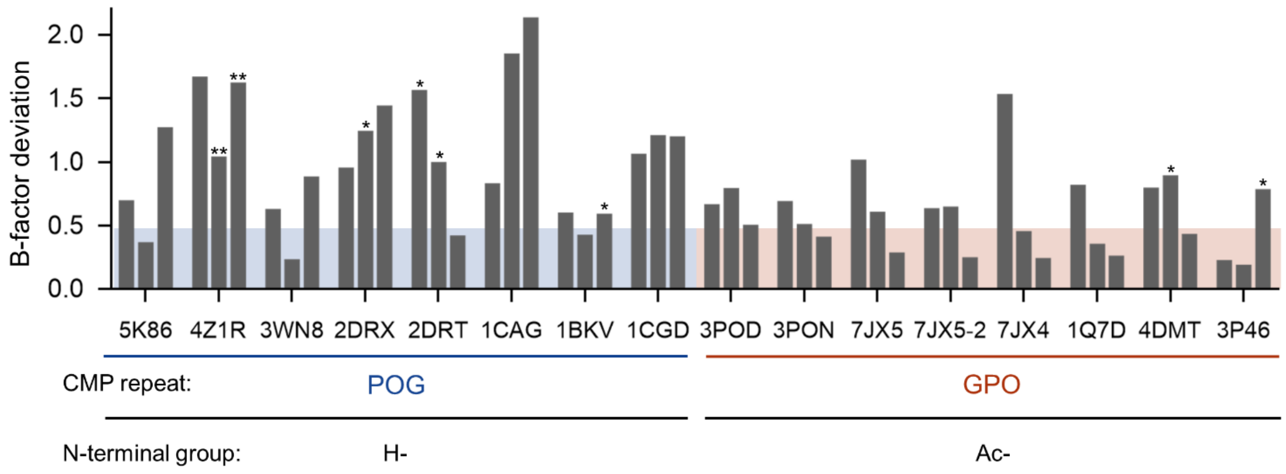

Figure S5. The deviation of atomic B-factors of the N-terminal amino acid triplet (POG *v.s.* GPO) from the mean B-factor of all non-hydrogen atoms in a given triple-helix. Unlike the GPO CMPs, none of the N-terminal POG featuring CMPs we found in PDB were N-acetylated. Each * indicates one unresolved and missing N-terminal amino acid residue in the crystal structure.

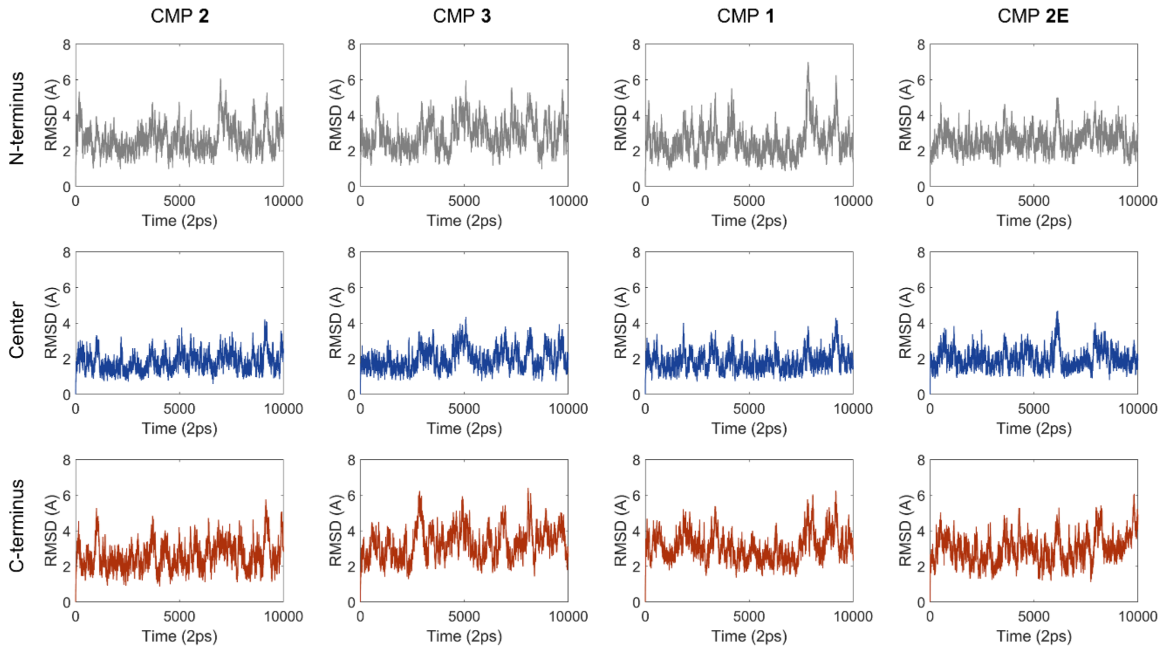

Figure S6. The root-mean-square deviation (RMSD) values of four CMP triple-helices at the N- and C-termini, and the sequence center.

CMP **2**: Ac-(GPO)$_7$-NH$_2$

CMP **3**: Ac-(GPO)$_7$G-NH$_2$

CMP **1**: Ac-(POG)$_7$-NH$_2$

CMP **2E**: Ac-(GPO)$_7$-OCH$_3$

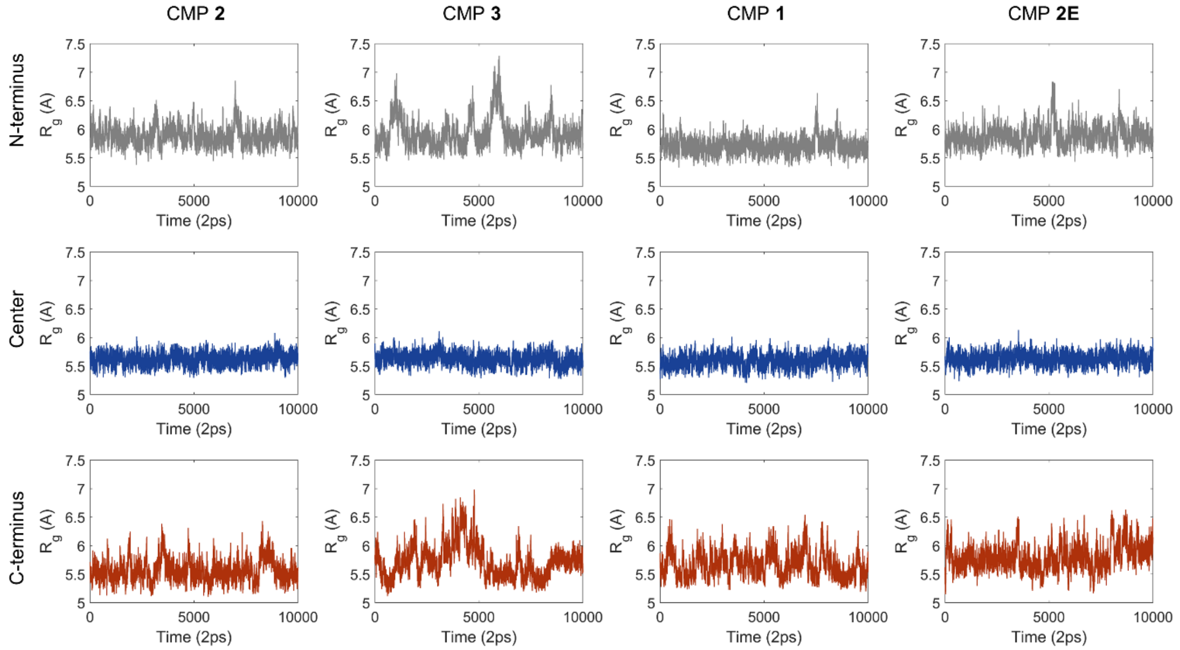

Figure S7. The radius of gyration (R_g) values of four CMP triple-helices at the N- and C-termini, and the sequence center.

CMP **2**: Ac-(GPO)$_7$-NH_2

CMP **3**: Ac-(GPO)$_7$G-NH_2

CMP **1**: Ac-(POG)$_7$-NH_2

CMP **2E**: Ac-(GPO)$_7$-OCH_3

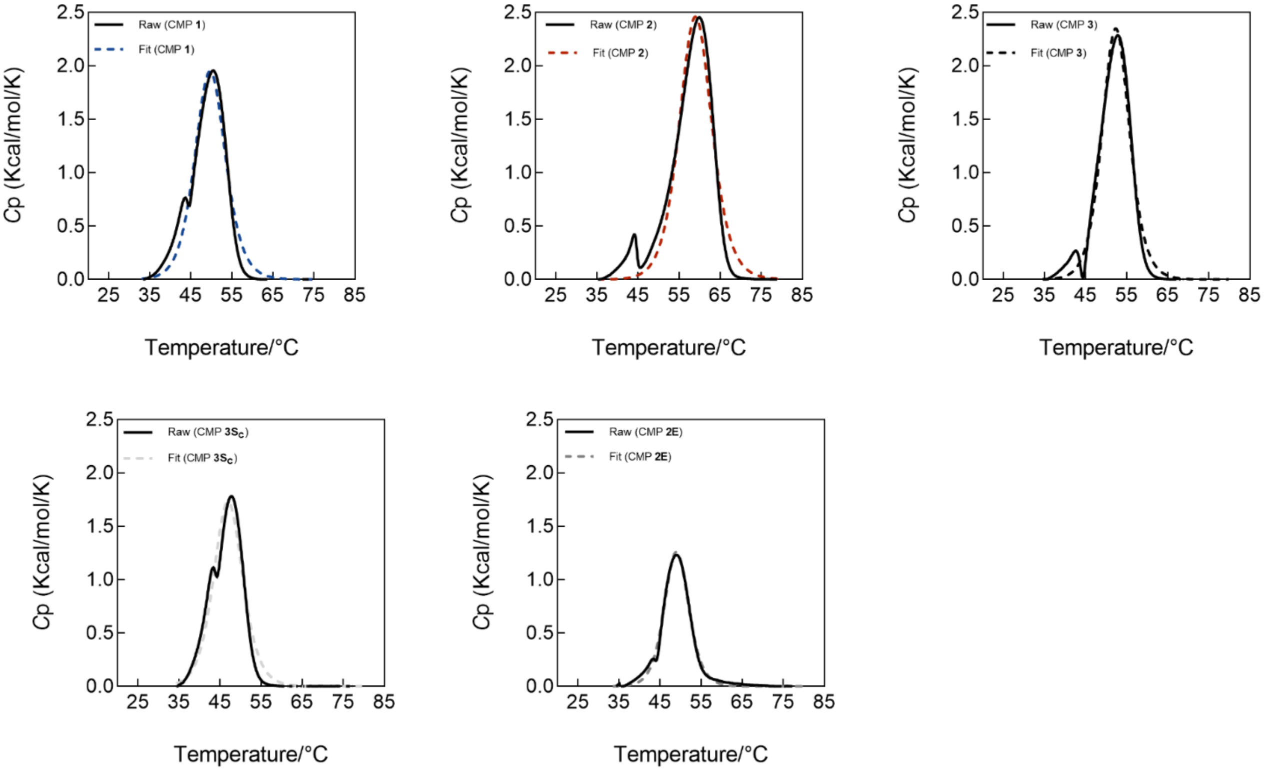

Figure S8. The experimental and fitting curves of the DSC thermograms.

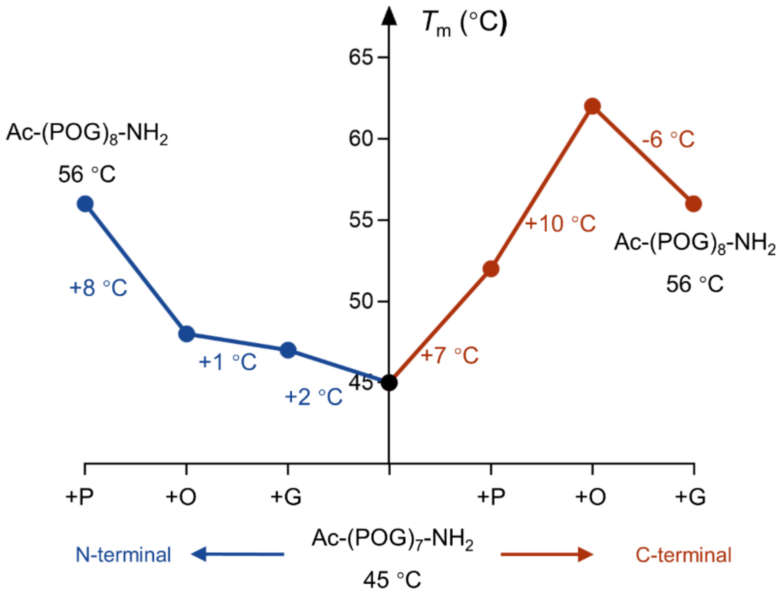

Figure S9. T_m value changes for stepwise incorporation of Gly, Hyp, and Pro to the N- or C-terminus of Ac-(POG)$_7$-NH$_2$.

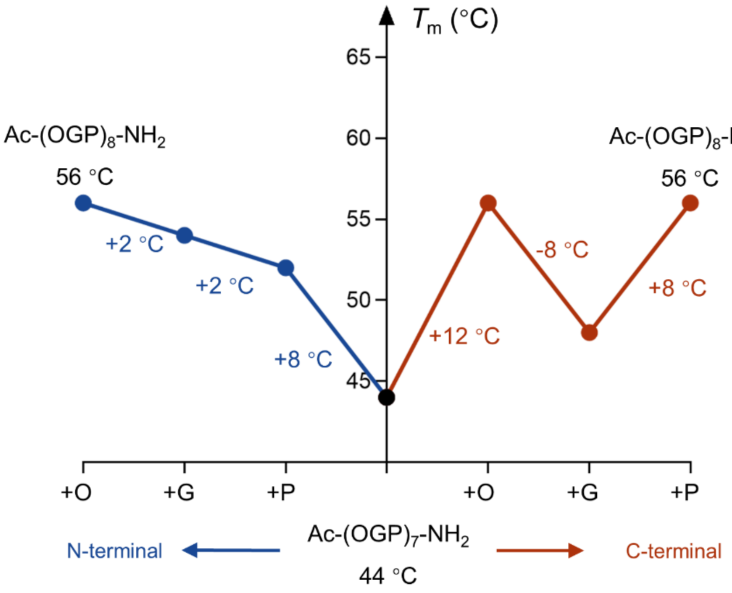

Figure S10. T_m value changes for stepwise incorporation of Pro, Gly, and Hyp to the N- or C-terminus of Ac-(OGP)$_7$-NH$_2$.

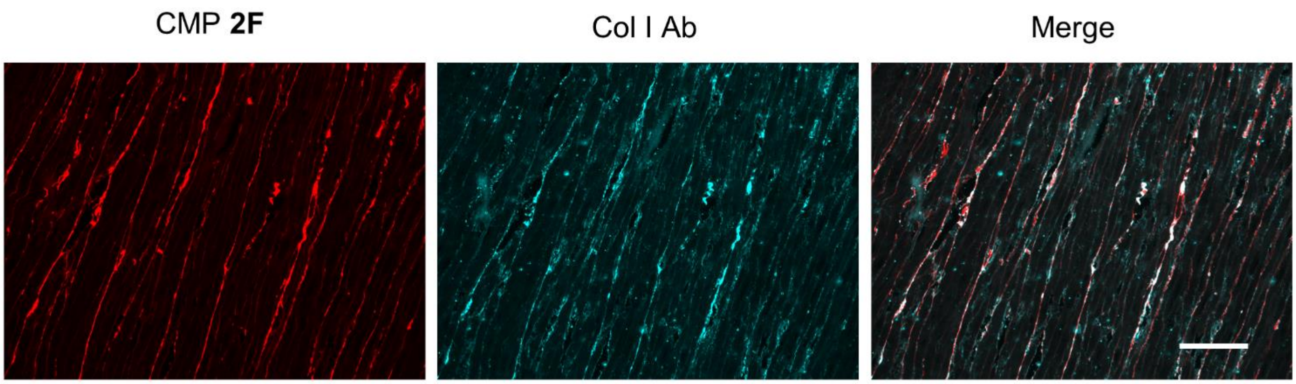

Figure S11. Micrographs of a paraffin-embedded section of normal rat heart tissue stained with CMP **2F** and an anti-collagen I antibody (after heat-mediated antigen retrieval to purposefully denature the collagen content) showing strongly overlapping CMP and antibody signals. Scale bar: 100 μm.

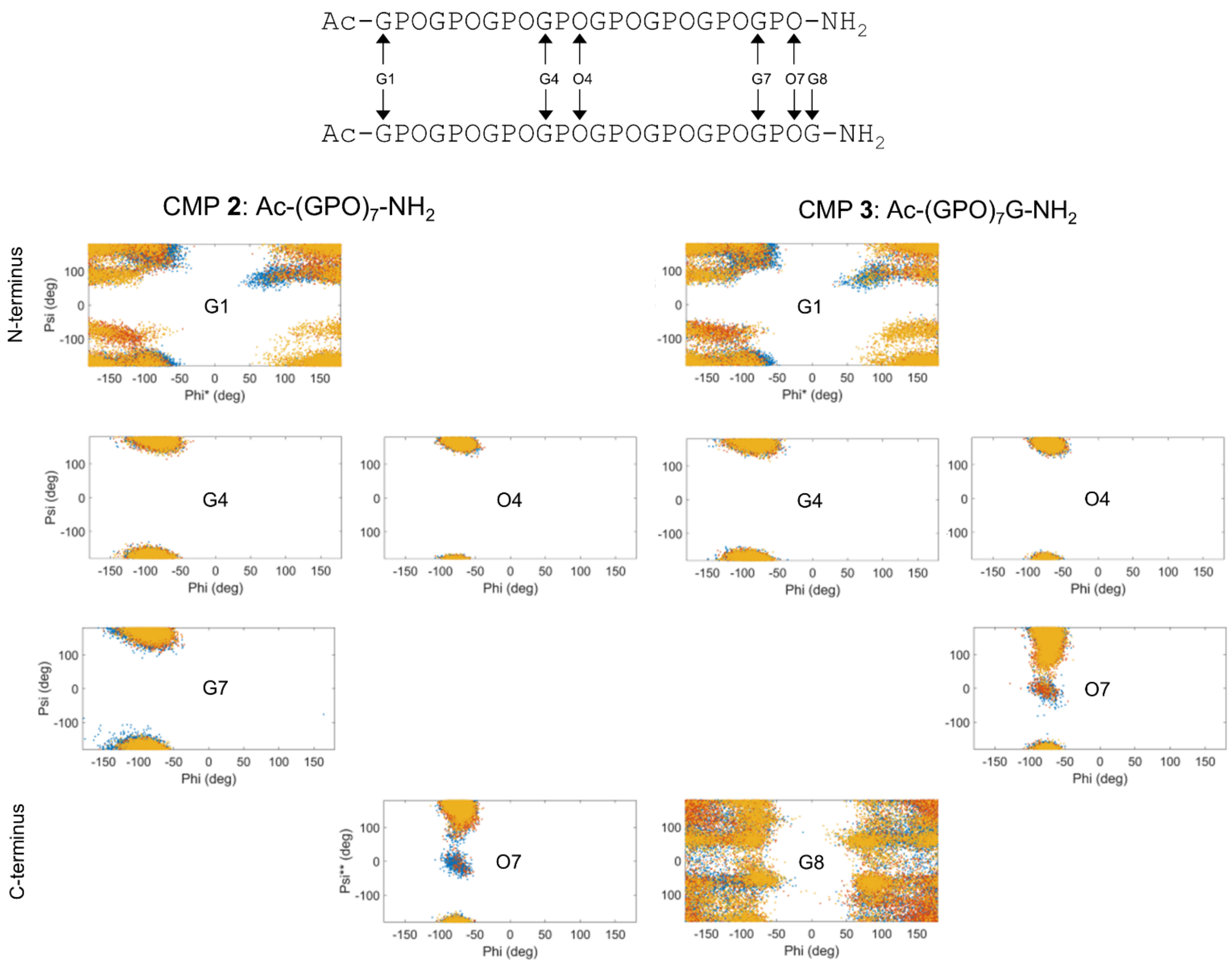

Figure S12. Distribution of φ and ψ angles of representitive animo acid residues in the MD simulated structural relaxation process for CMP **2** and **3**, showing that Gly was more flexible than Hyp at the C-terminus. Each color refers to the residues in one single CMP chain (orange: chain 1; red: chain 2; blue: chain 3).

*The phi (φ) angle for the N-terminal residue G1 is defined by Cy-N-Cα-C, where Cy is the carbon atom of the acetyl group attached to residue G1; ** The psi (ψ) angle for the C-terminal reisude O7 (CMP **2**) or G8 (CMP **3**) is defined by N-Cα-C-Nt, where Nt is the nitrogen atom of the amide group connecting to the last residue.

Supplementary Section 3: MALDI and CD of all peptides

CMP **1** Ac-(POG)$_7$-NH_2

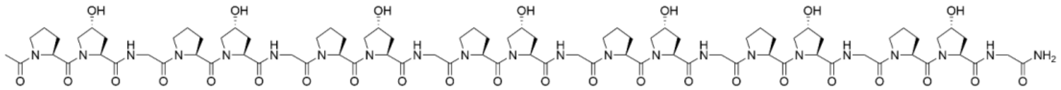

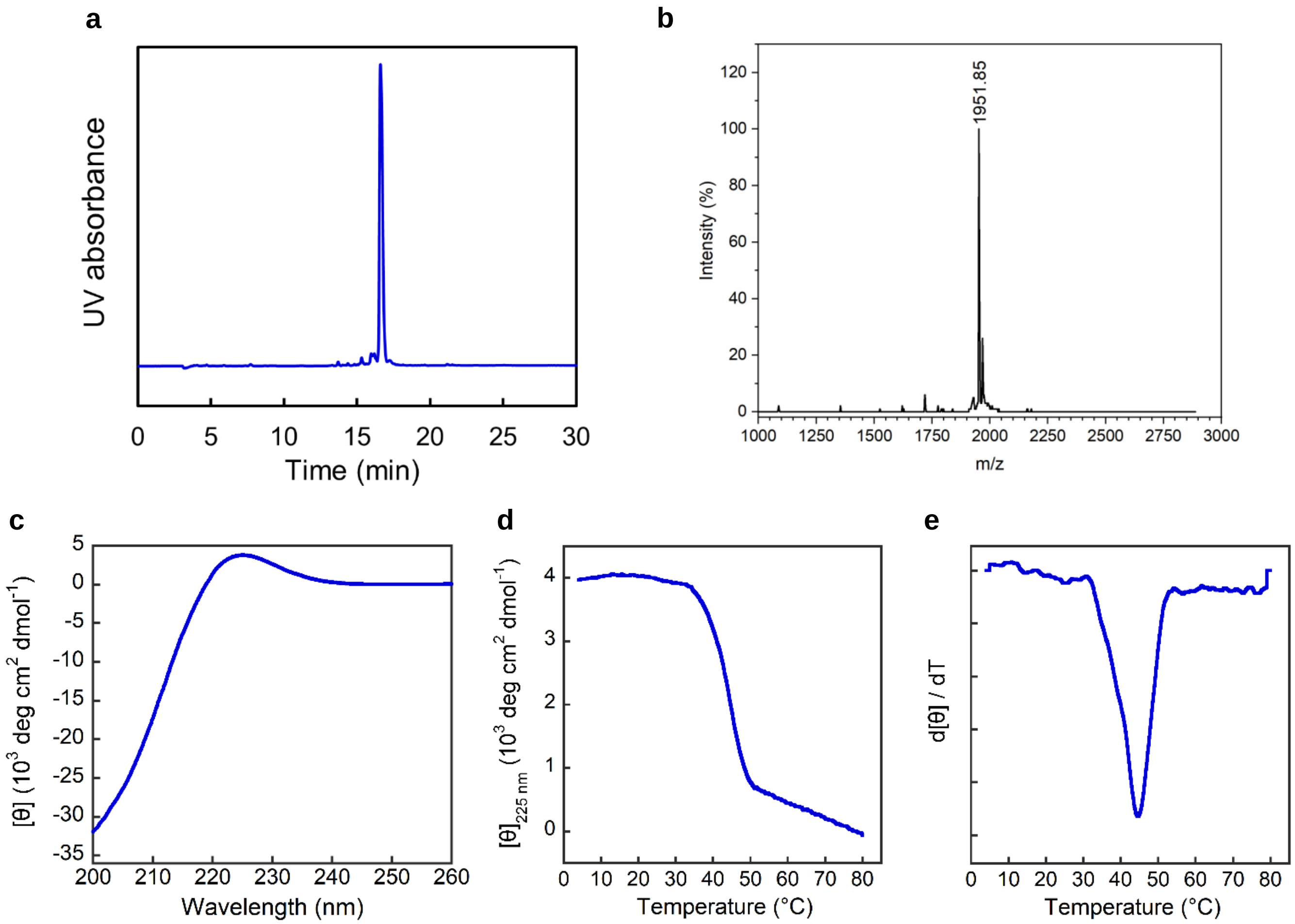

a, The HPLC chromatogram of purified peptide, t_R = 16.6 min. The purity is 96%.

b, MALDI-MS, calculated: 1951.9 $[M+Na]^+$, observed: 1951.9 $[M+Na]^+$, 1968.5 $[M+K]^+$.

c, The CD spectrum in PBS buffer at 4 °C.

d, The CD thermal melting curve in PBS buffer.

e, The first derivative of the melting curve, T_m = 45 °C.

CMP **2** Ac-(GPO)$_7$-NH$_2$

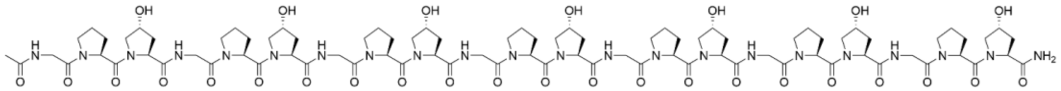

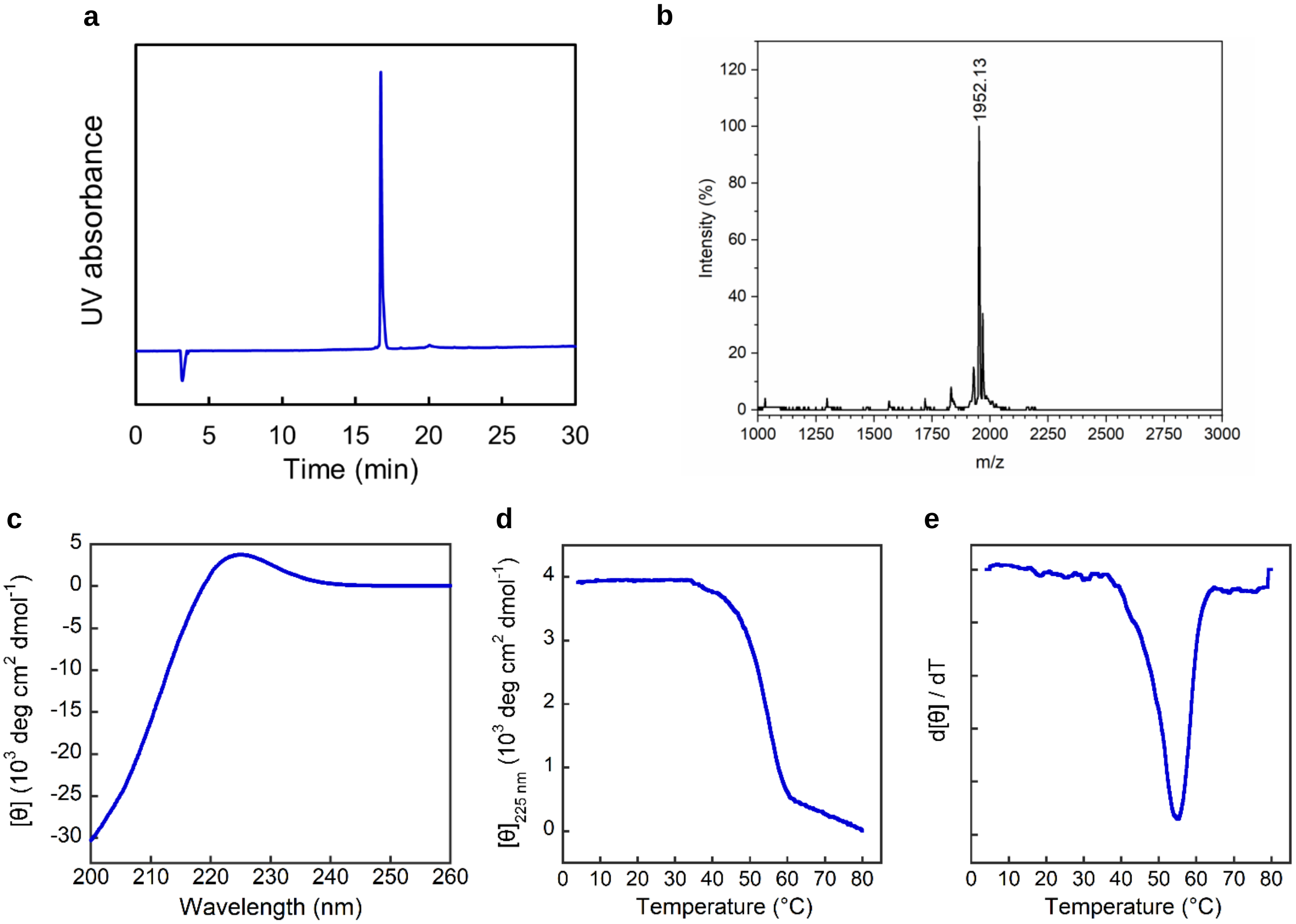

a, The HPLC chromatogram of purified peptide, t_R = 16.7 min. The purity is 99%.

b, MALDI-MS, calculated: 1951.9 $[M+Na]^+$, observed: 1952.1 $[M+Na]^+$, 1968.2 $[M+K]^+$.

c, The CD spectrum in PBS buffer at 4 °C.

d, The CD thermal melting curve in PBS buffer.

e, The first derivative of the melting curve, T_m = 55 °C.

CMP **2O** Ac-(GPO)$_7$-OH

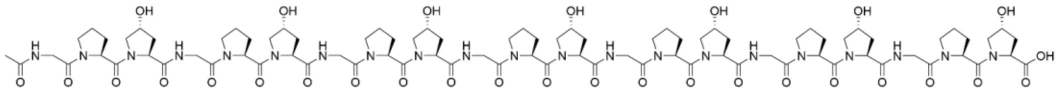

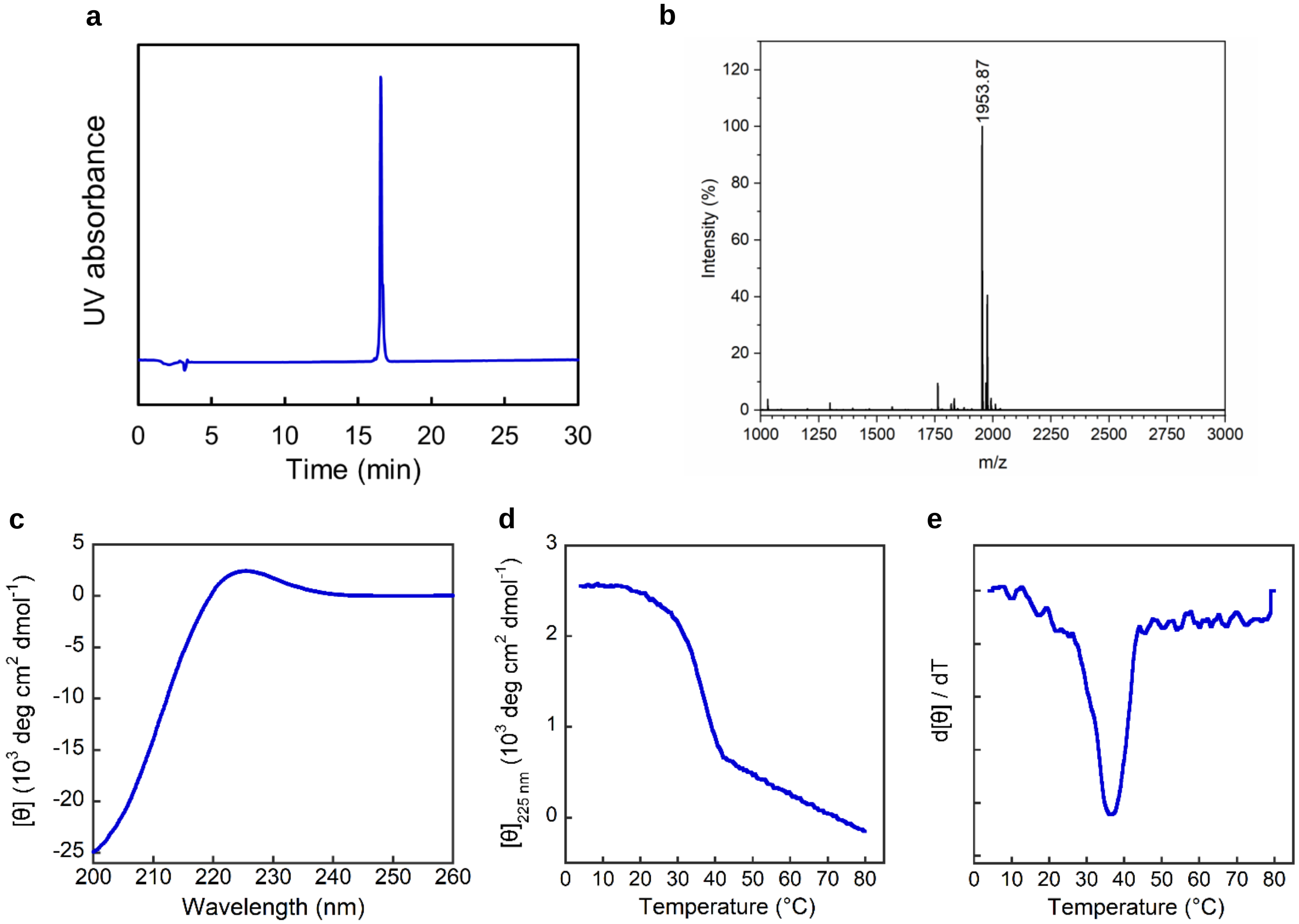

a, The HPLC chromatogram of purified peptide, t_R = 16.5 min. The purity is 99%.

b, MALDI-MS, calculated: 1952.9 $[M+Na]^+$, observed: 1953.9 $[M+Na]^+$.

b, The CD spectrum in PBS buffer at 4 °C.

c, The CD thermal melting curve in PBS buffer.

d, The first derivative of the melting curve, T_m = 36 °C.

CMP **2E** Ac-$(GPO)_7$-OCH_3

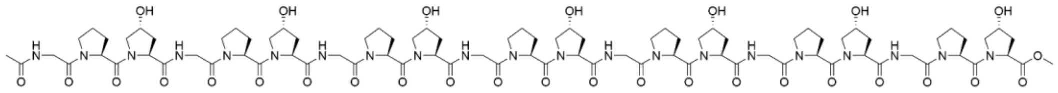

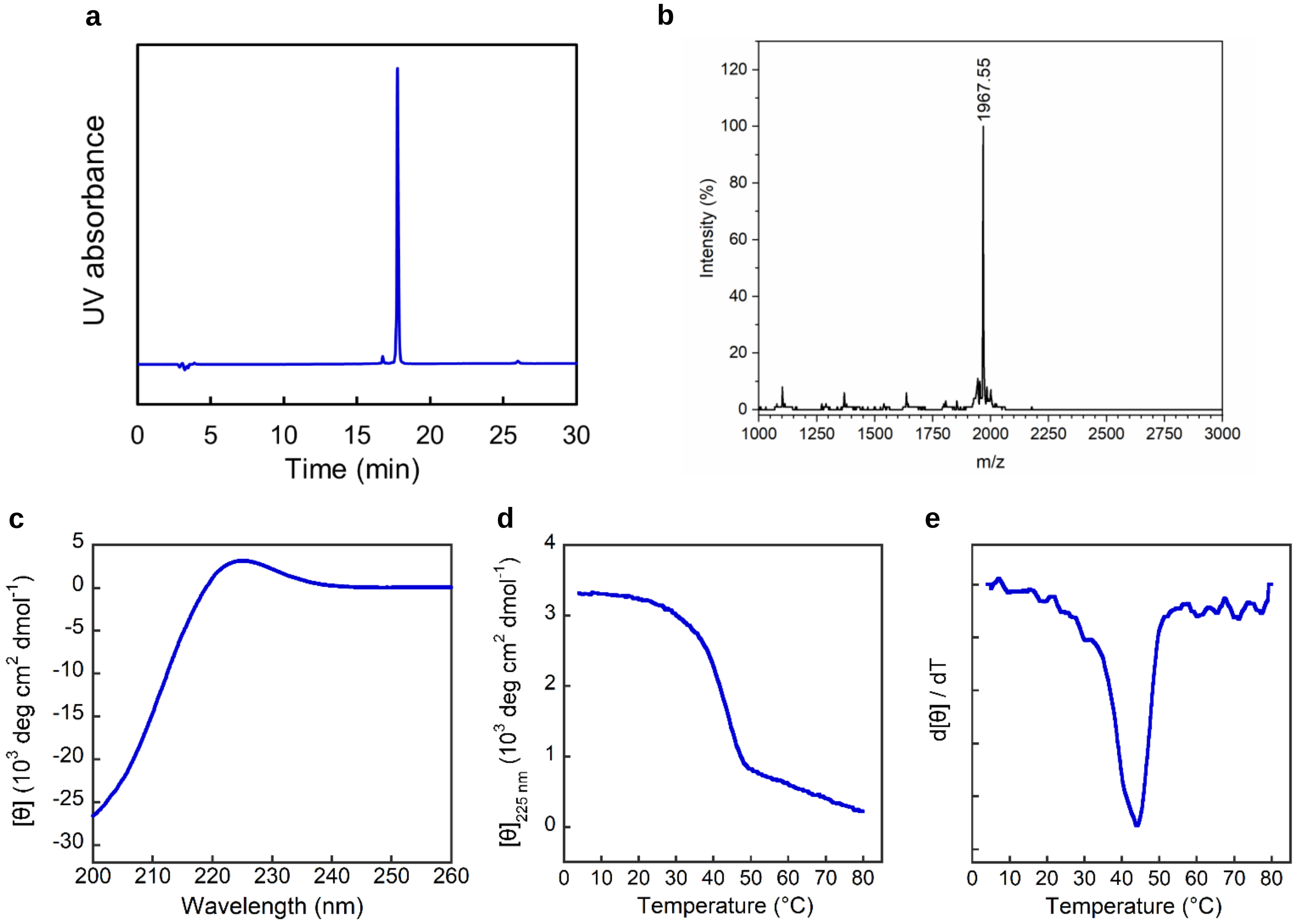

a, The HPLC chromatogram of purified peptide, t_R = 17.8 min. The purity is 98%.

b, MALDI-MS, calculated: 1966.9 $[M+Na]^+$, observed: 1967.6 $[M+Na]^+$.

c, The CD spectrum in PBS buffer at 4 °C.

d, The CD thermal melting curve in PBS buffer.

e, The first derivative of the melting curve, T_m = 44 °C.

CMP **3** Ac-G(POG)$_7$-NH$_2$ = Ac-(GPO)$_7$G-NH$_2$

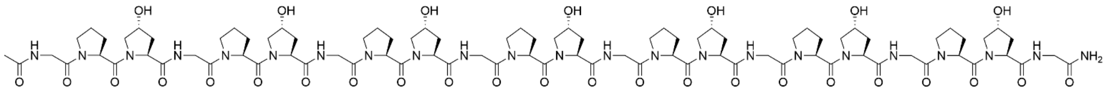

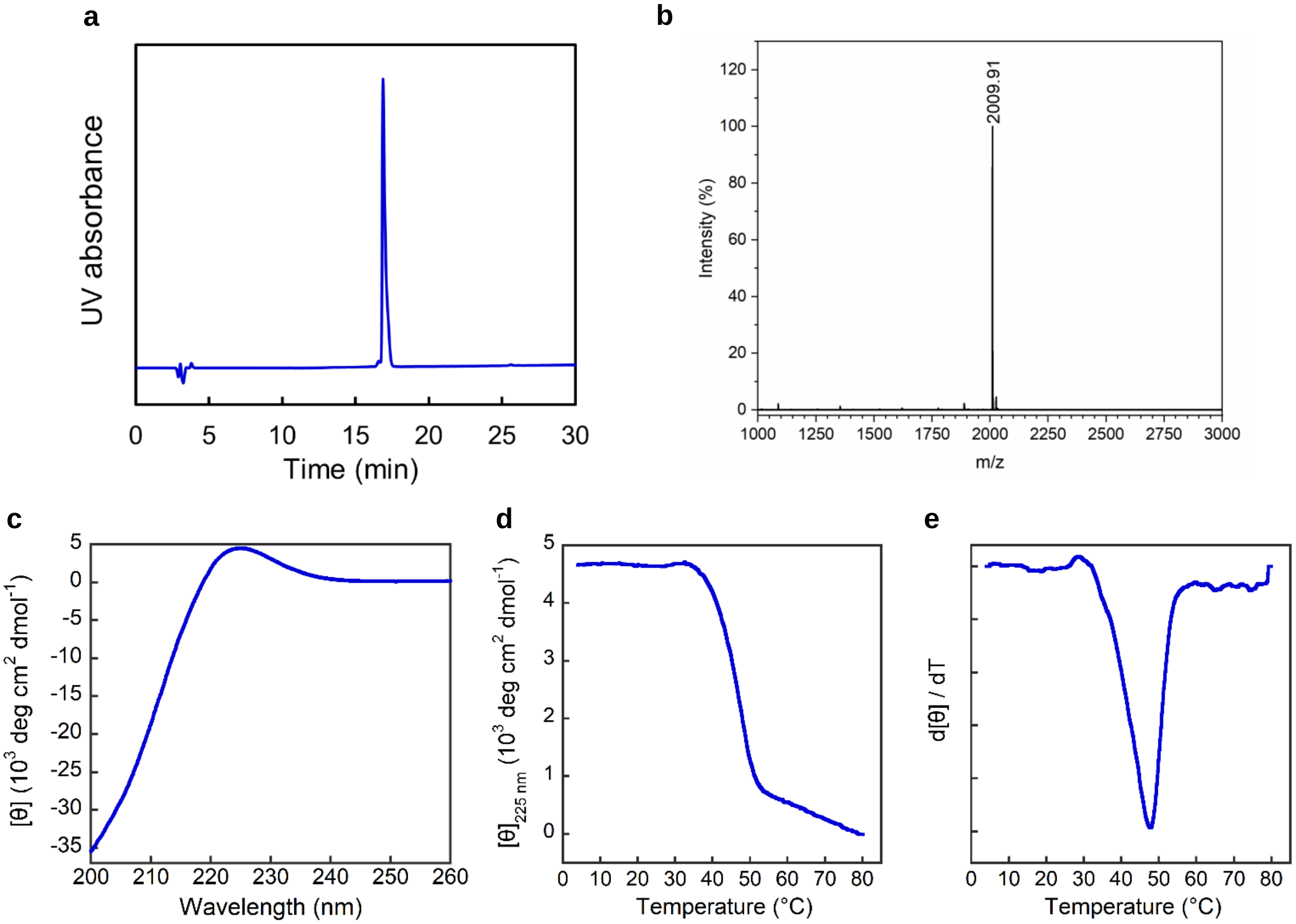

a, The HPLC chromatogram of purified peptide, t_R = 16.9 min. The purity is 98%.

b, MALDI-MS, calculated: 2008.9 [M+Na]$^+$, observed: 2009.9 [M+Na]$^+$.

c, The CD spectrum in PBS buffer at 4 °C.

d, The CD thermal melting curve in PBS buffer.

e, The first derivative of the melting curve, T_m = 47 °C.

CMP **3A** H-G(POG)$_7$-NH$_2$ = H-(GPO)$_7$G-NH$_2$

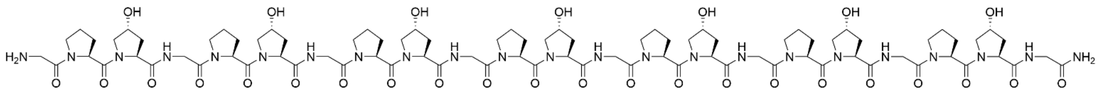

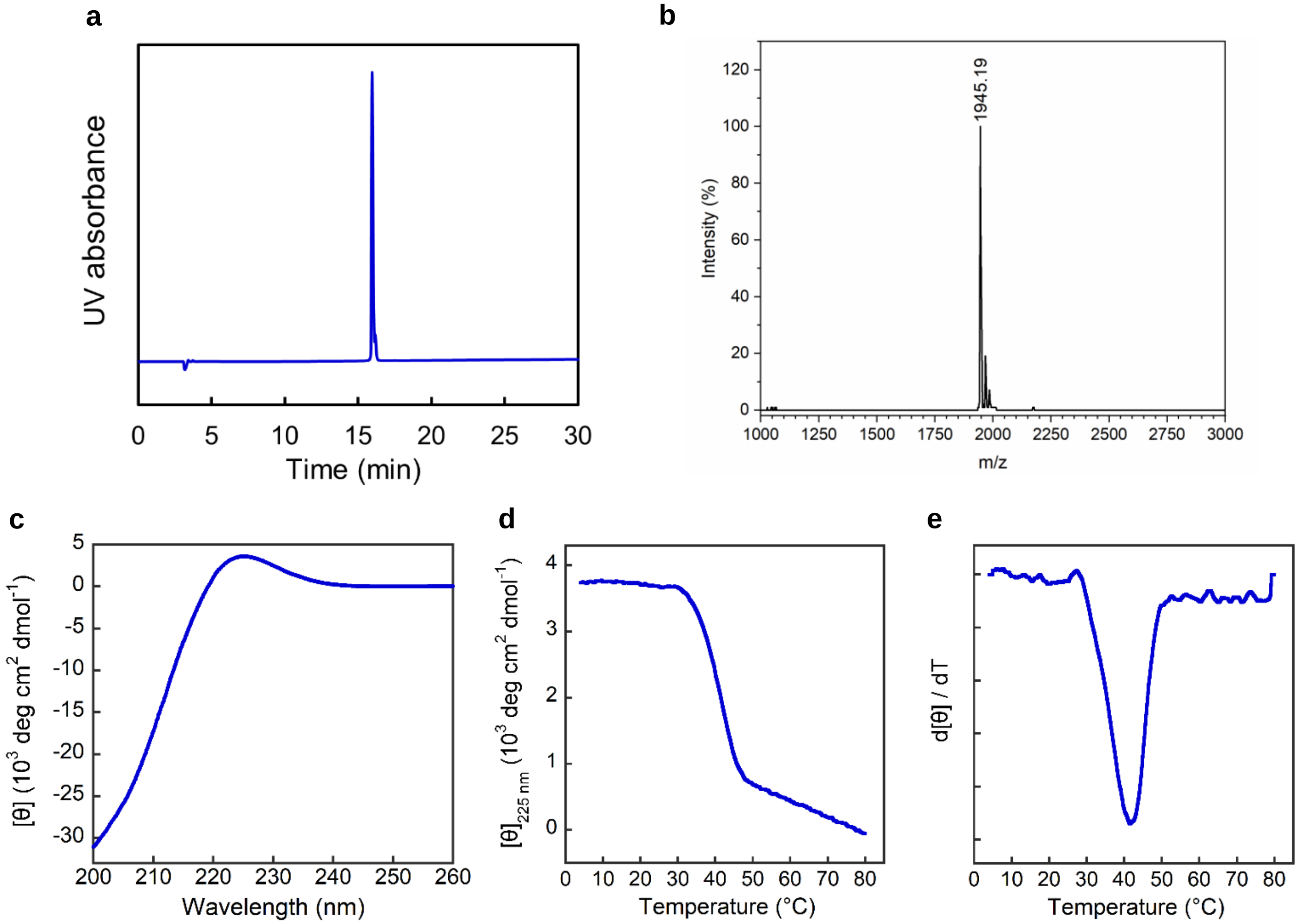

a, The HPLC chromatogram of purified peptide, t_R = 16.0 min. The purity is 96%.

b, MALDI-MS, calculated: 1944.9 $[M+H]^+$, observed: 1945.2 $[M+H]^+$, 1967.7 $[M+Na]^+$.

c, The CD spectrum in PBS buffer at 4 °C.

d, The CD thermal melting curve in PBS buffer.

e, The first derivative of the melting curve, T_m = 42 °C.

CMP **3S**$_N$ Ac-Sar(POG)$_7$-NH$_2$

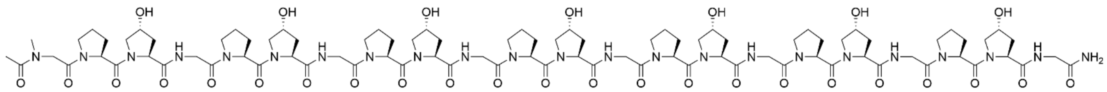

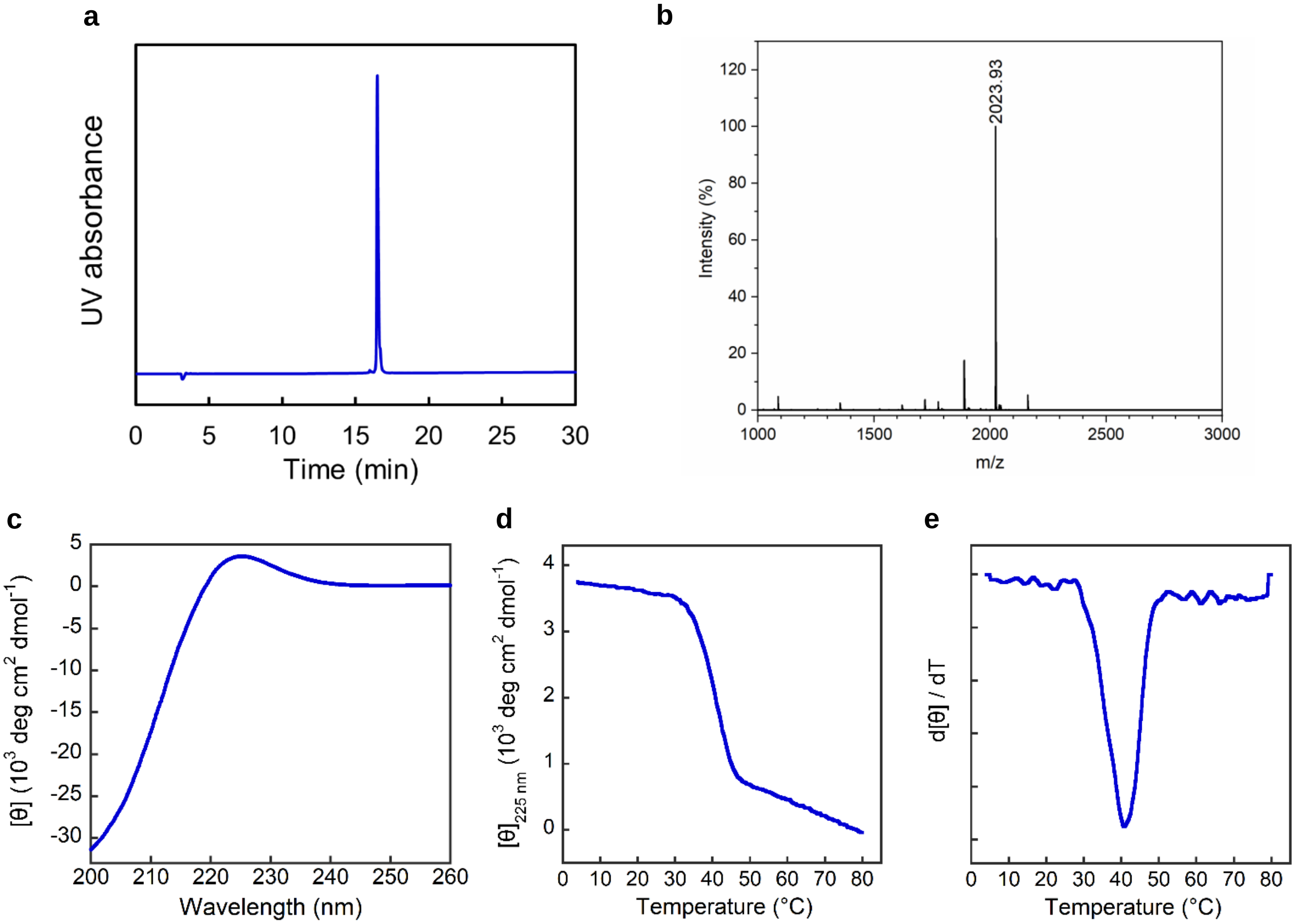

a, The HPLC chromatogram of purified peptide, t_R = 16.5 min. The purity is 99%.

b, MALDI-MS, calculated: 2022.9 $[M+Na]^+$, observed: 2023.9 $[M+Na]^+$.

c, The CD spectrum in PBS buffer at 4 °C.

d, The CD thermal melting curve in PBS buffer.

e, The first derivative of the melting curve, T_m = 41 °C.

CMP **3S**$_C$ Ac-(GPO)$_7$Sar-NH$_2$

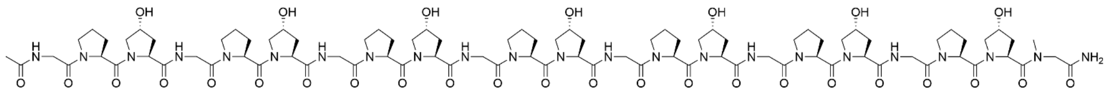

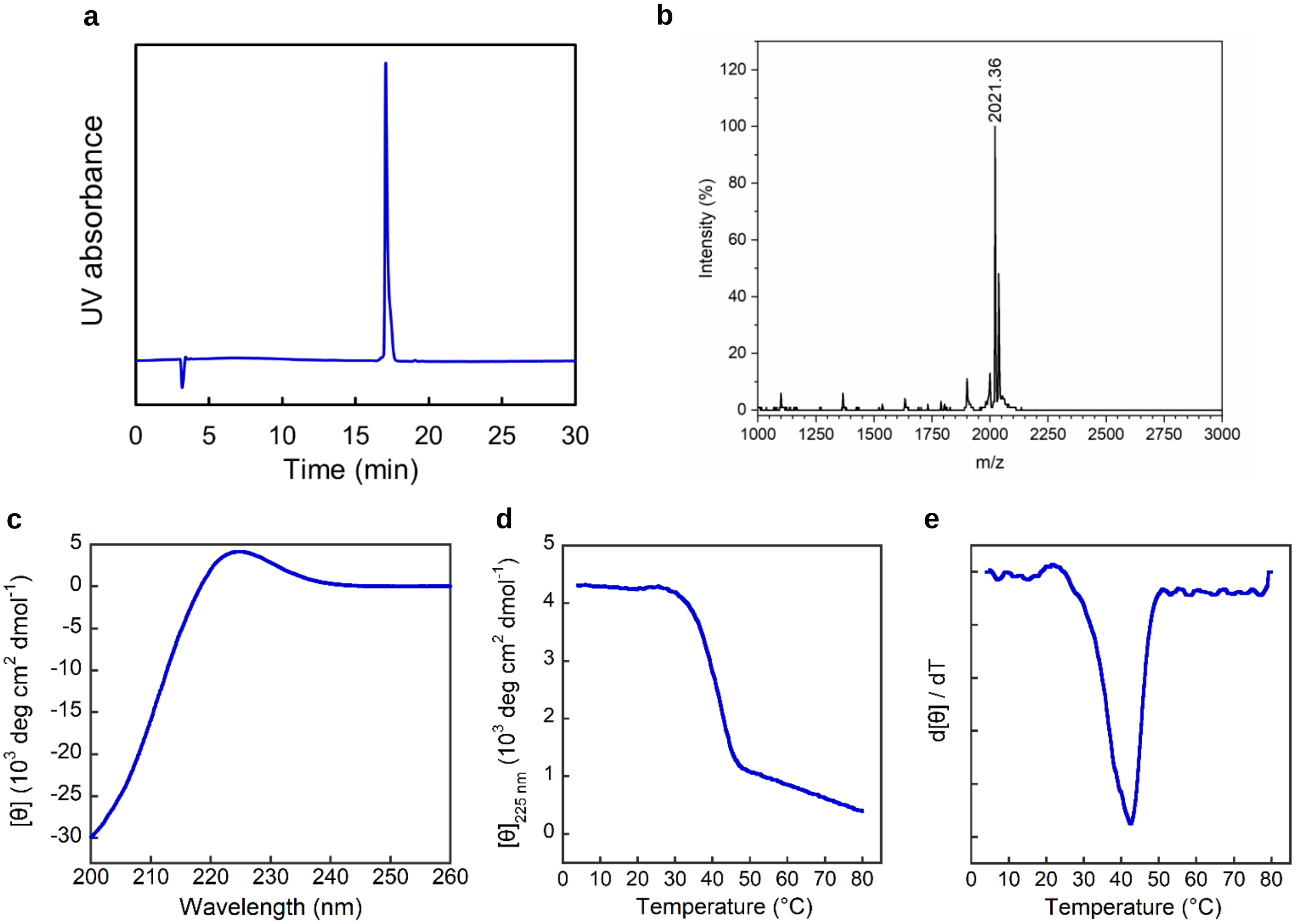

a, The HPLC chromatogram of purified peptide, t_R = 17.1 min. The purity is 97%.

b, MALDI-MS, calculated: 2022.9 $[M+Na]^+$, observed: 2021.4 $[M+Na]^+$, 2037.4 $[M+K]^+$.

c, The CD spectrum in PBS buffer at 4 °C.

d, The CD thermal melting curve in PBS buffer.

e, The first derivative of the melting curve, T_m = 42 °C.

CMP **4** Ac-(OGP)$_7$-NH_2

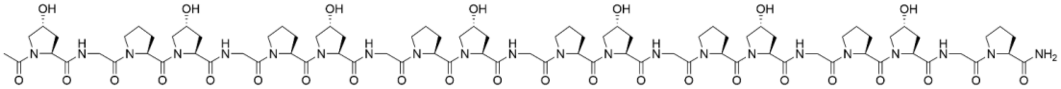

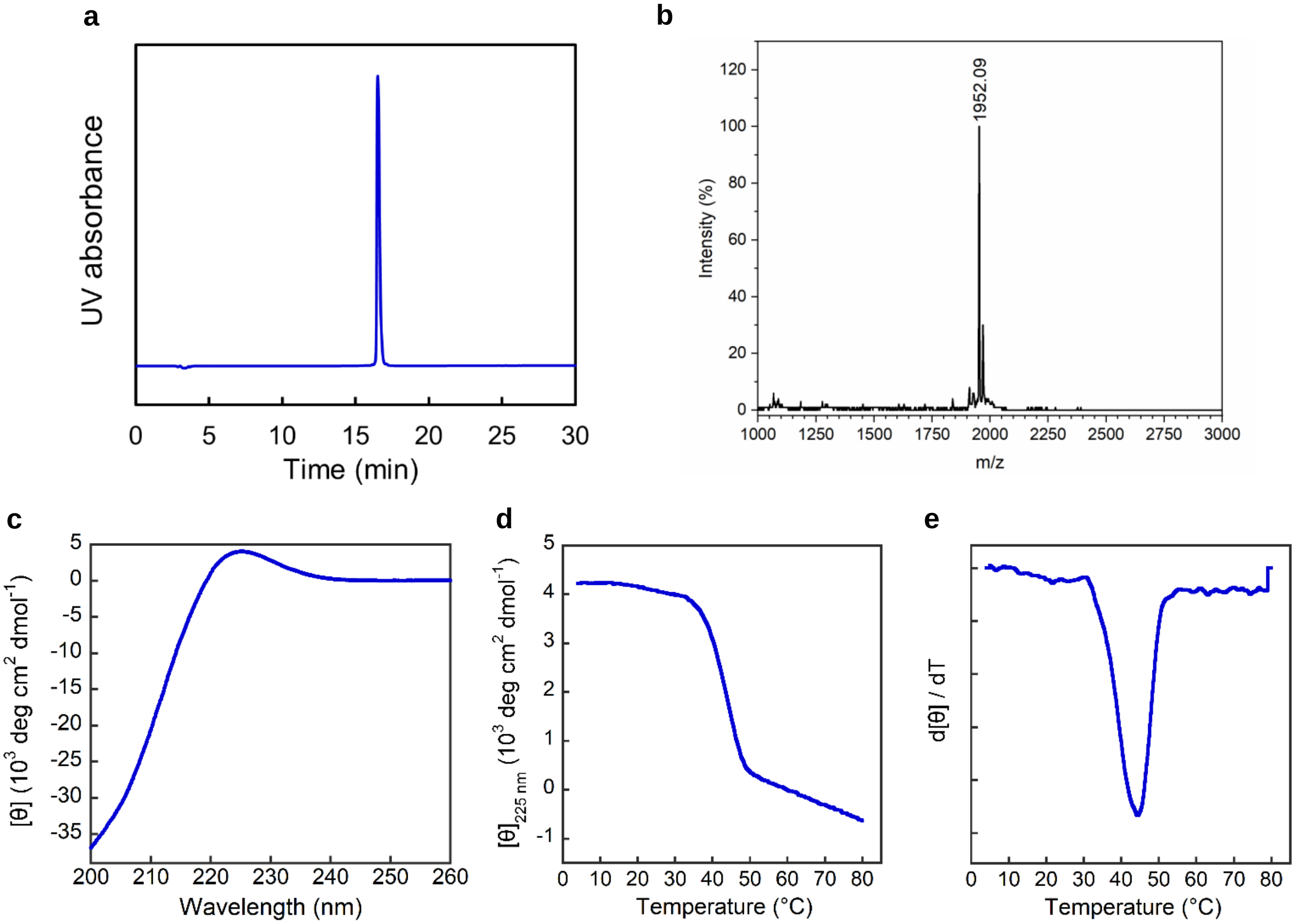

a, The HPLC chromatogram of purified peptide, t_R = 16.5 min. The purity is 99%.

b, MALDI-MS, calculated: 1951.9 [M+Na]$^+$, observed: 1952.1 [M+Na]$^+$, 1970.3 [M+Na]$^+$.

c, The CD spectrum in PBS buffer at 4 °C.

d, The CD thermal melting curve in PBS buffer.

e, The first derivative of the melting curve, T_m = 44 °C.

CMP **5** Ac-P(OGP)$_7$-NH$_2$ = Ac-(POG)$_7$P-NH$_2$

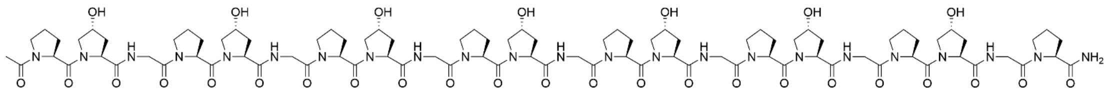

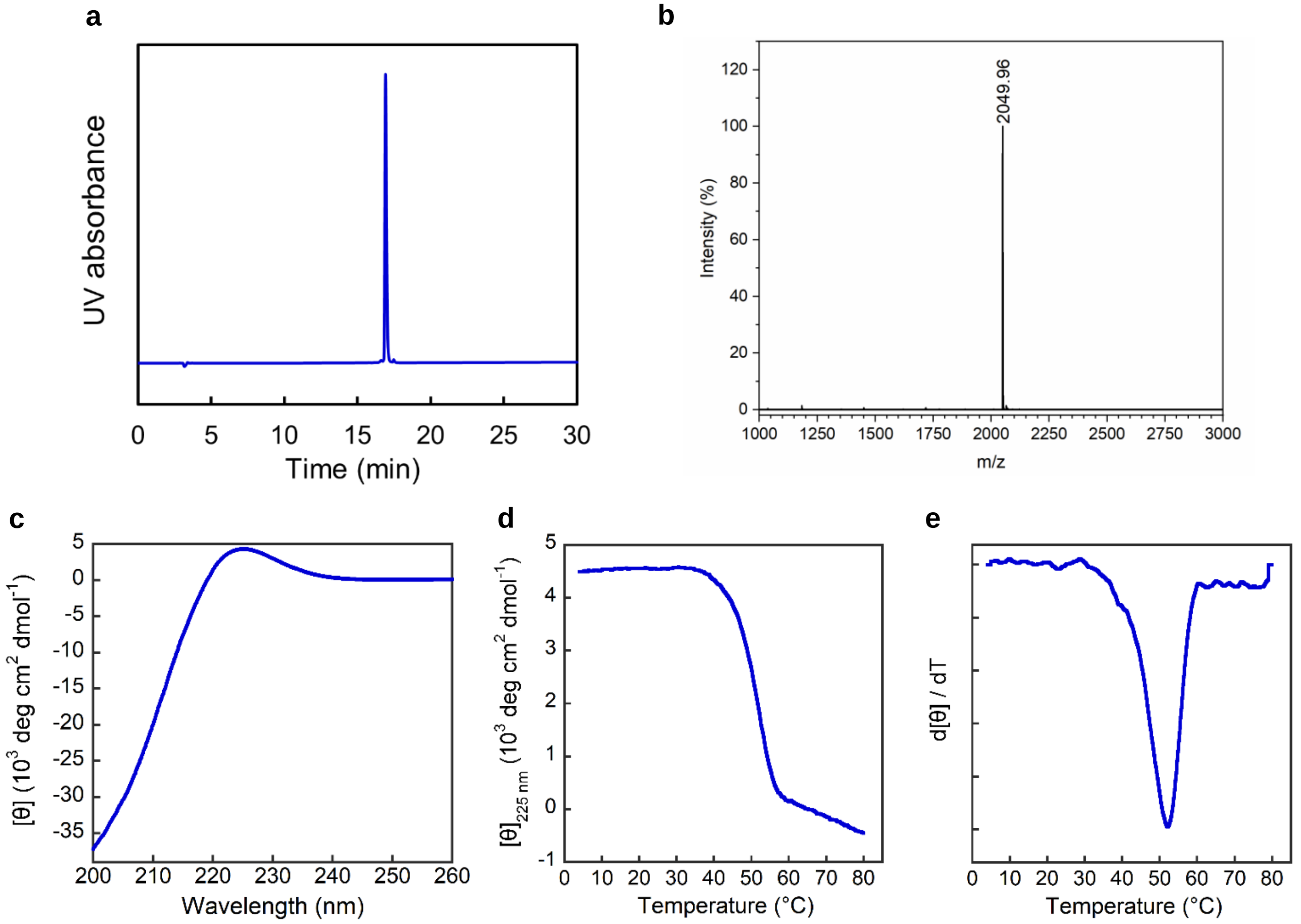

a, The HPLC chromatogram of purified peptide, t_R = 16.9 min. The purity is 99%.

b, MALDI-MS, calculated: 2048.9 [M+Na]$^+$, observed: 2050.0 [M+Na]$^+$.

c, The CD spectrum in PBS buffer at 4 °C.

d, The CD thermal melting curve in PBS buffer.

e, The first derivative of the melting curve, T_m = 52 °C.

CMP **6** Ac-O(GPO)$_7$-NH$_2$ = Ac-(OGP)$_7$O-NH$_2$

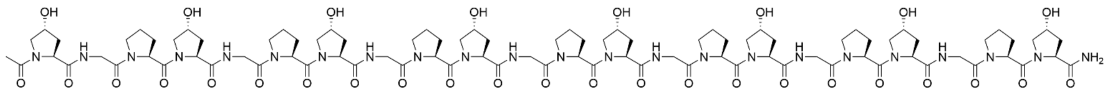

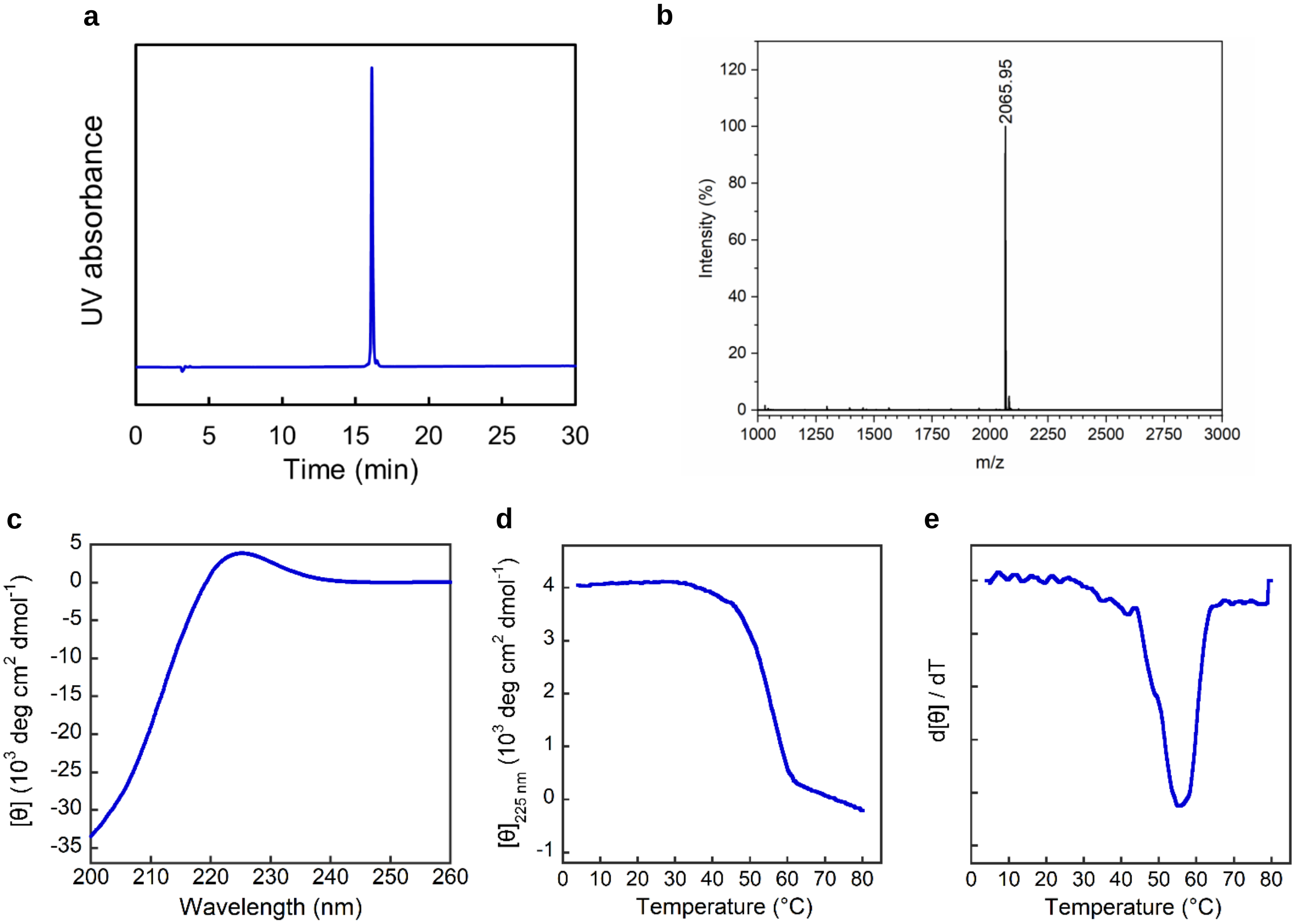

a, The HPLC chromatogram of purified peptide, t_R = 16.1 min. The purity is 98%.

b, MALDI-MS, calculated: 2064.9 [M+Na]$^+$, observed: 2066.0 [M+Na]$^+$.

c, The CD spectrum in PBS buffer at 4 °C.

d, The CD thermal melting curve in PBS buffer.

e, The first derivative of the melting curve, T_m = 56 °C.

Ac-(POG)$_5$-NH$_2$

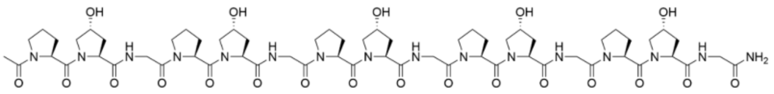

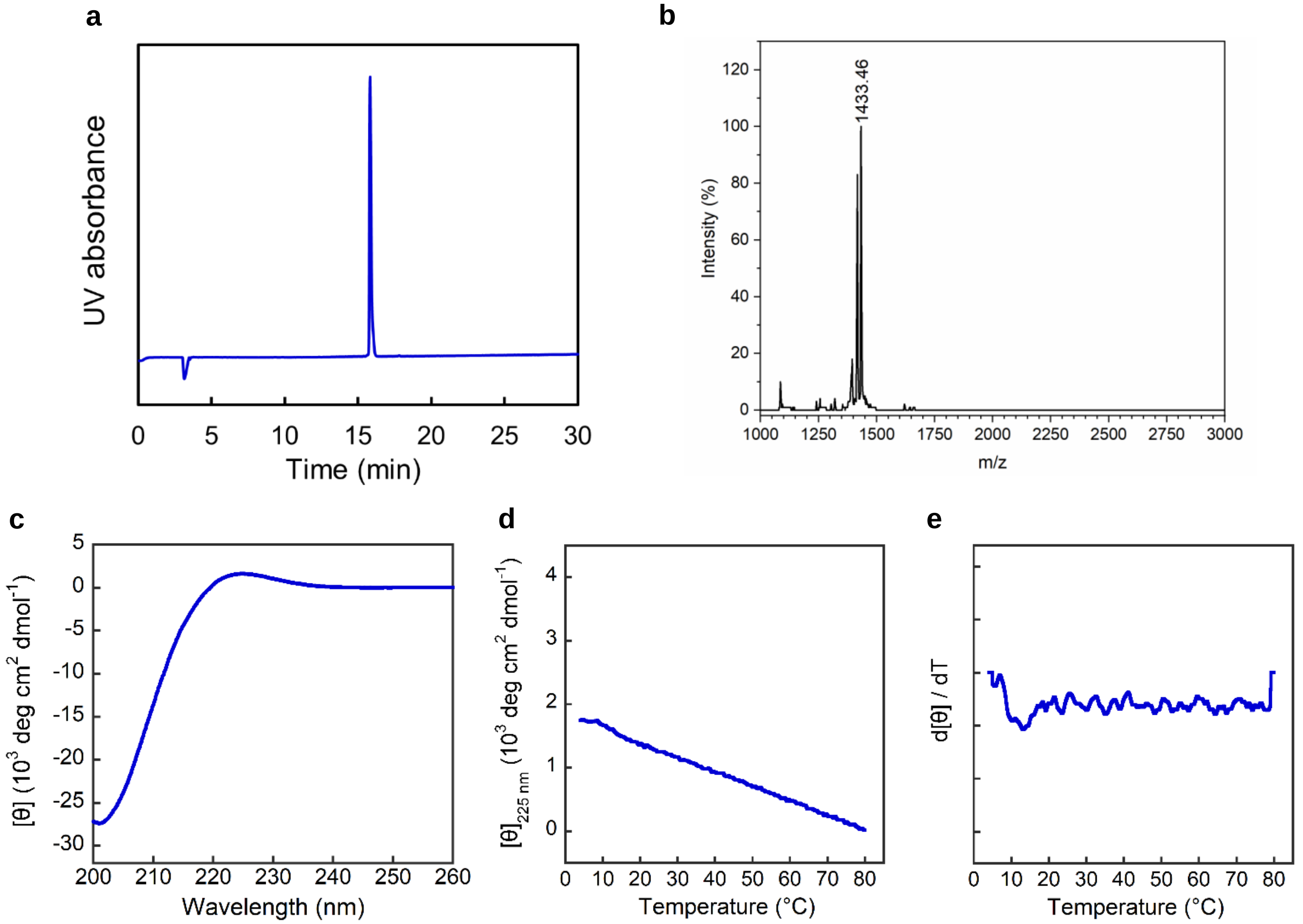

a, The HPLC chromatogram of purified peptide, t_R = 15.8 min. The purity is 99%.

b, MALDI-MS, calculated: 1417.7 $[M+Na]^+$, observed: 1417.3 $[M+Na]^+$, 1433.5 $[M+K]^+$.

c, The CD spectrum in PBS buffer at 4 °C.

d, The CD thermal melting curve in PBS buffer.

e, The first derivative of the melting curve, no T_m.

Ac-(POG)$_6$-NH$_2$

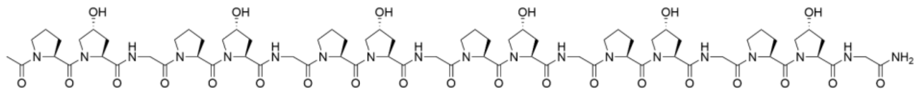

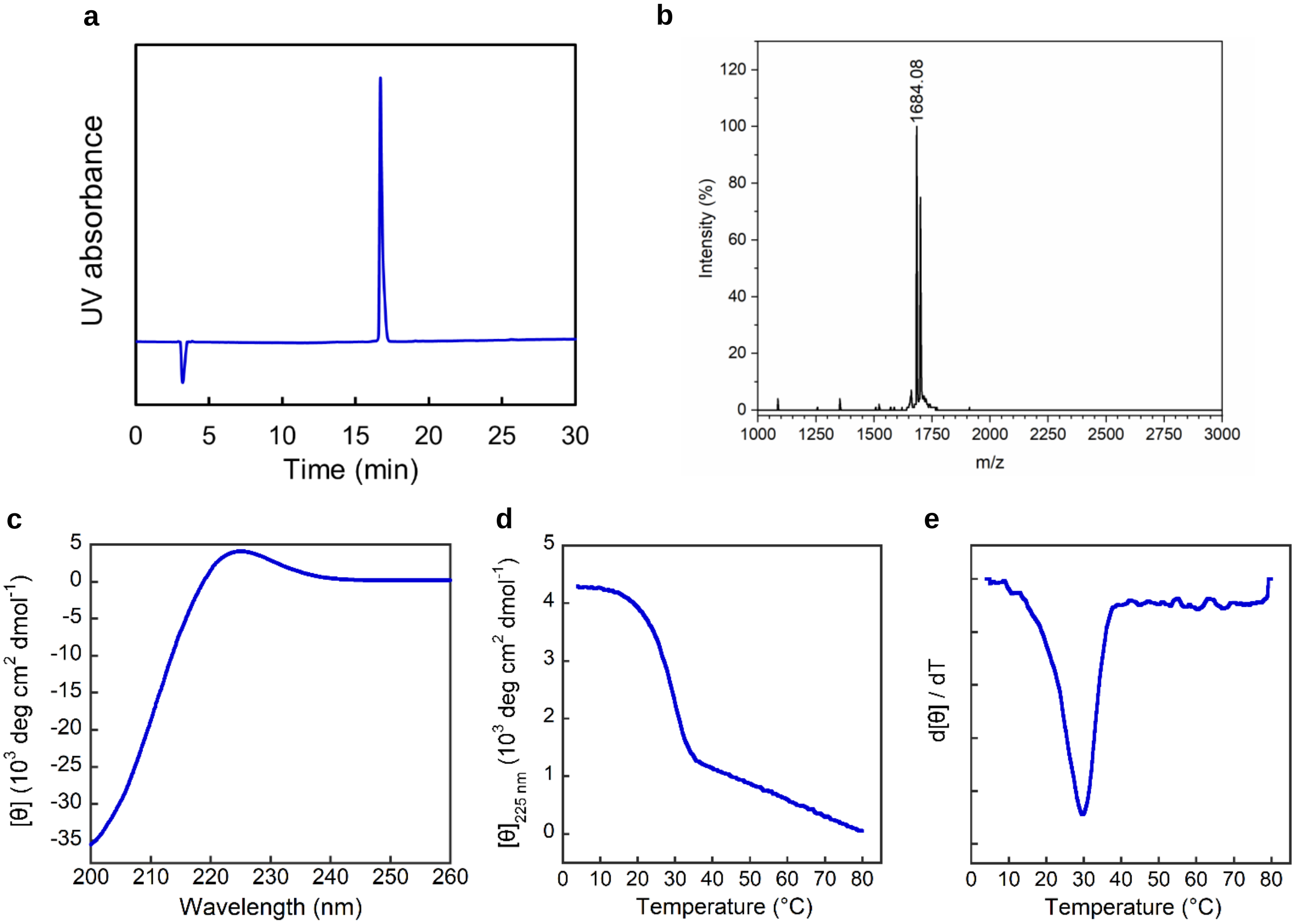

a, The HPLC chromatogram of purified peptide, t_R = 16.7 min. The purity is 99%.

b, MALDI-MS, calculated: 1684.8 $[M+Na]^+$, observed: 1684.1 $[M+Na]^+$, 1700.1 $[M+K]^+$.

c, The CD spectrum in PBS buffer at 4 °C.

d, The CD thermal melting curve in PBS buffer.

e, The first derivative of the melting curve, T_m = 30 °C.

H-$(POG)_7$-NH_2

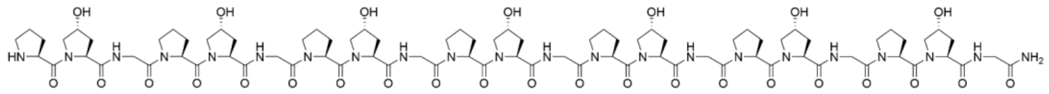

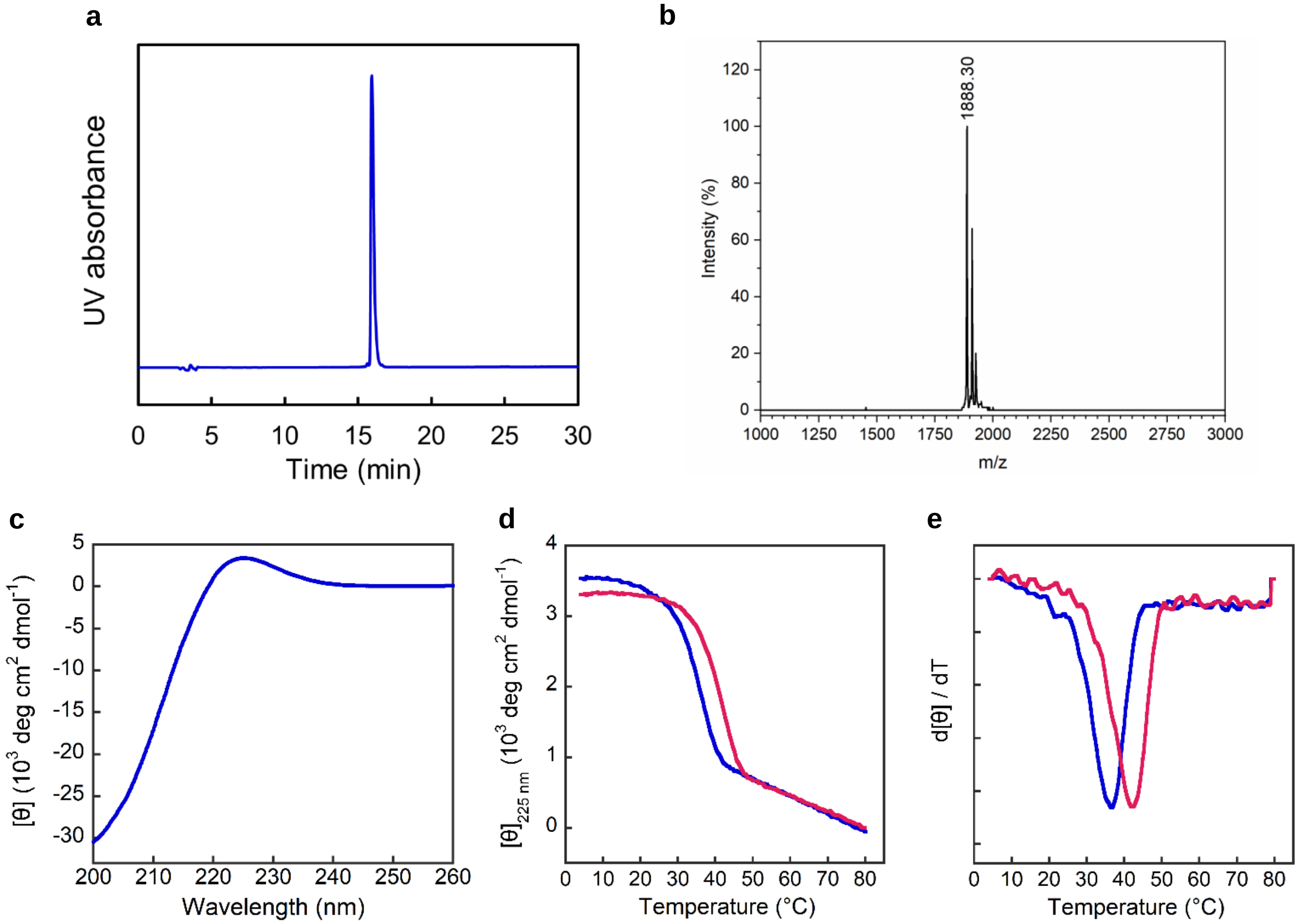

a, The HPLC chromatogram of purified peptide, t_R = 15.9 min. The purity is 99%.

b, MALDI-MS, calculated: 1887.9 $[M+H]^+$, observed: 1888.3 $[M+H]^+$, 1909.4 $[M+Na]^+$, 1926.4 $[M+K]^+$.

c, The CD spectrum in PBS buffer at 4 °C.

d, The CD thermal melting curve in PBS buffer (blue) and 3.5 mM NaOH solution (pH 11.5, red).

e, The first derivative of the melting curve, T_m = 37 °C (PBS, blue) or 42 °C (NaOH, red).

Ac-(POG)$_7$-OH

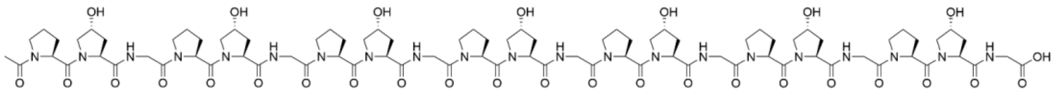

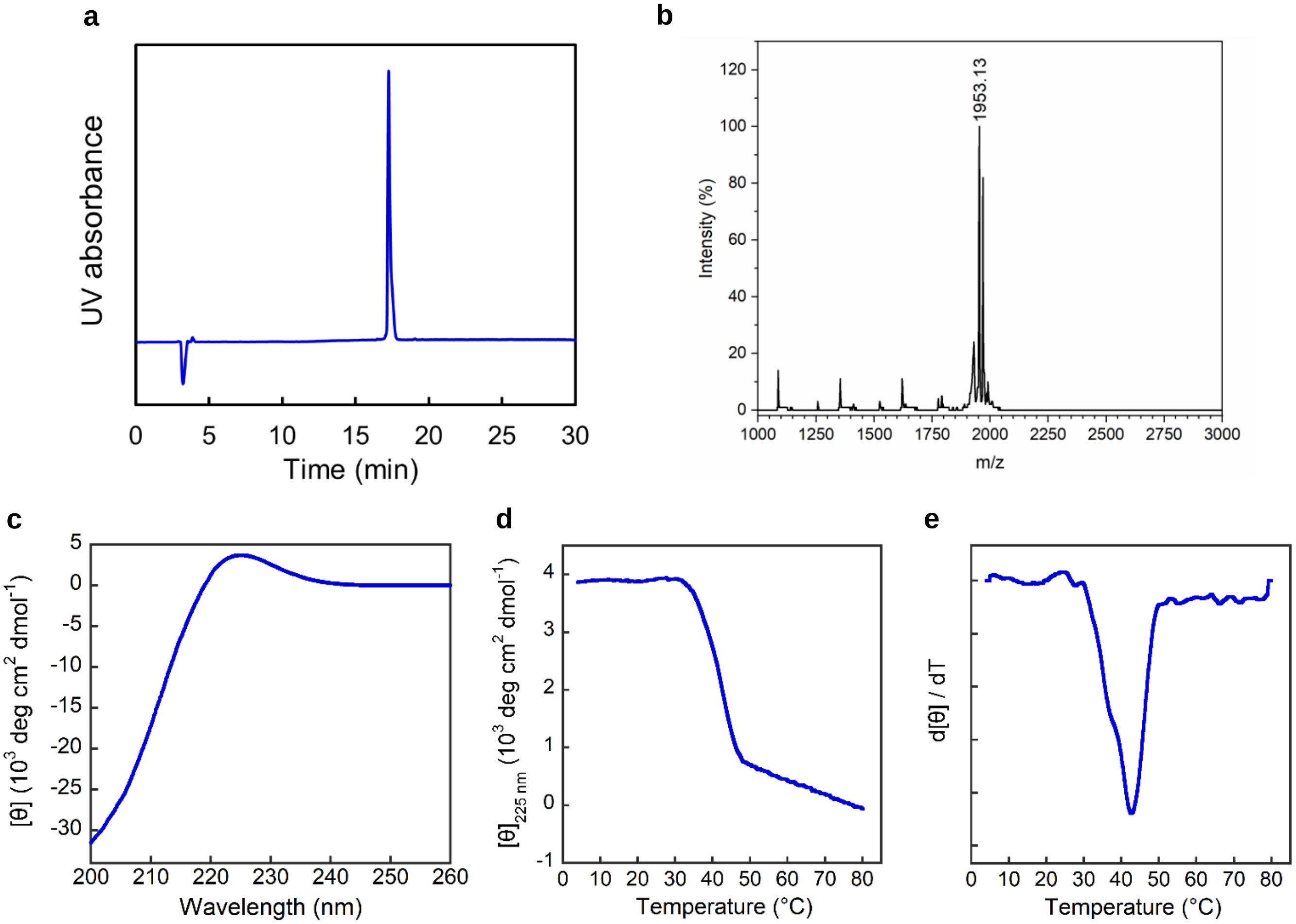

a, The HPLC chromatogram of purified peptide, t_R = 17.3 min. The purity is 99%.

b, MALDI-MS, calculated: 1952.9 $[M+Na]^+$, observed: 1953.1 $[M+Na]^+$, 1969.2 $[M+K]^+$.

c, The CD spectrum in PBS buffer at 4 °C.

d, The CD thermal melting curve in PBS buffer.

e, The first derivative of the melting curve, T_m = 42 °C.

Ac-(POG)$_8$-NH$_2$

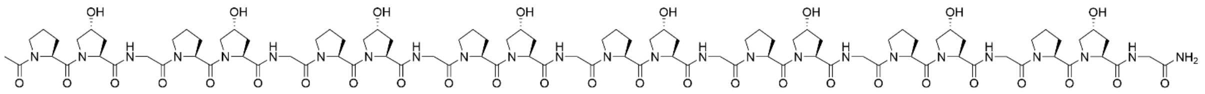

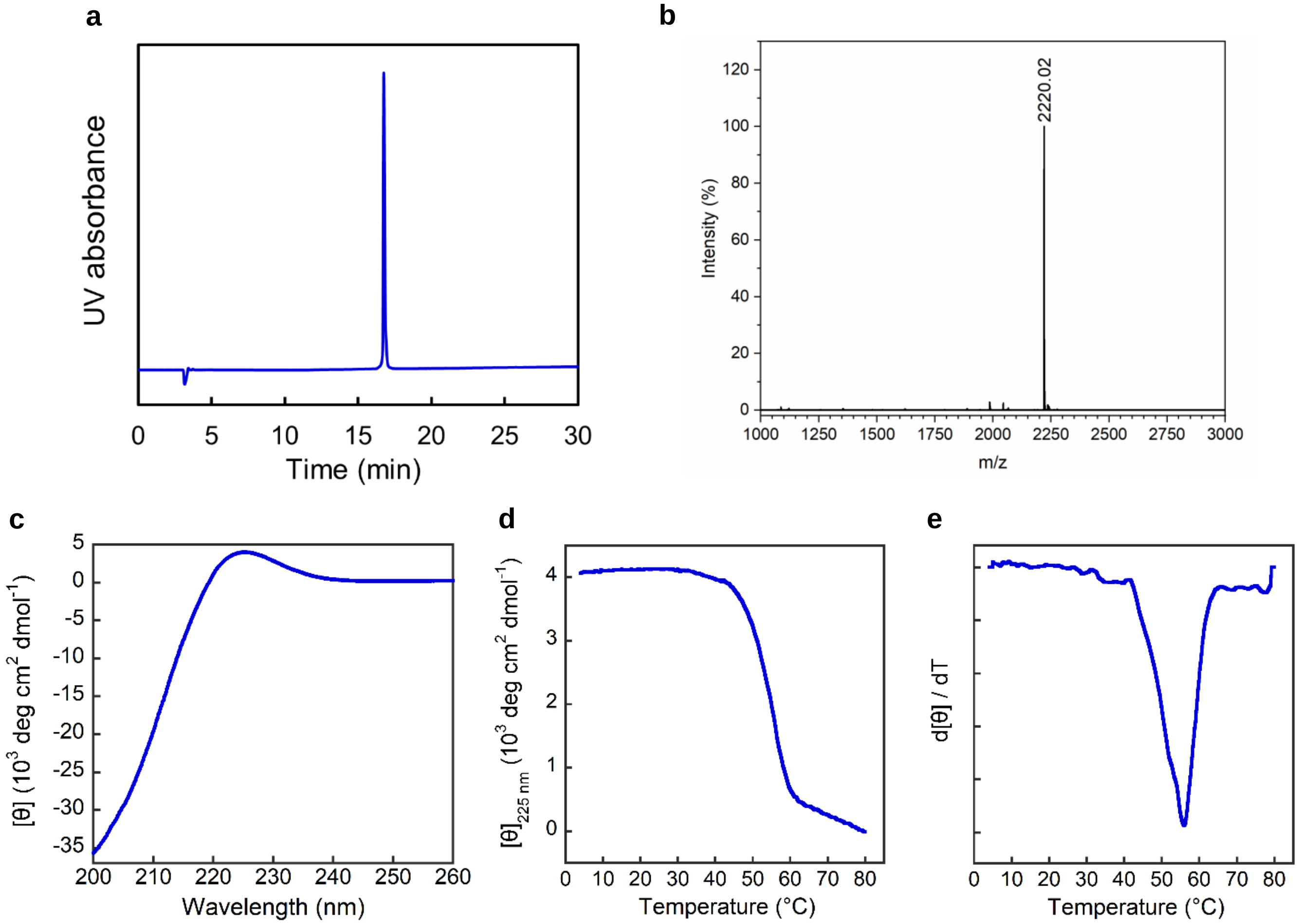

a, The HPLC chromatogram of purified peptide, t_R = 16.8 min. The purity is 98%.

b, MALDI-MS, calculated: 2219.0 $[M+Na]^+$, observed: 2220.0 $[M+Na]^+$.

c, The CD spectrum in PBS buffer at 4 °C.

d, The CD thermal melting curve in PBS buffer.

e, The first derivative of the melting curve, T_m = 56 °C.

Ac-(POG)$_9$-NH$_2$

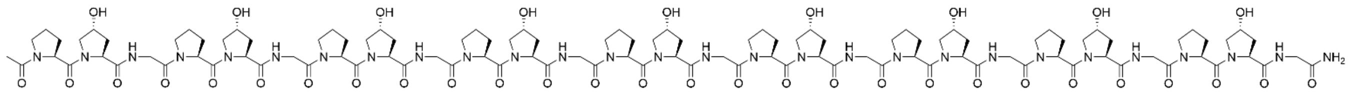

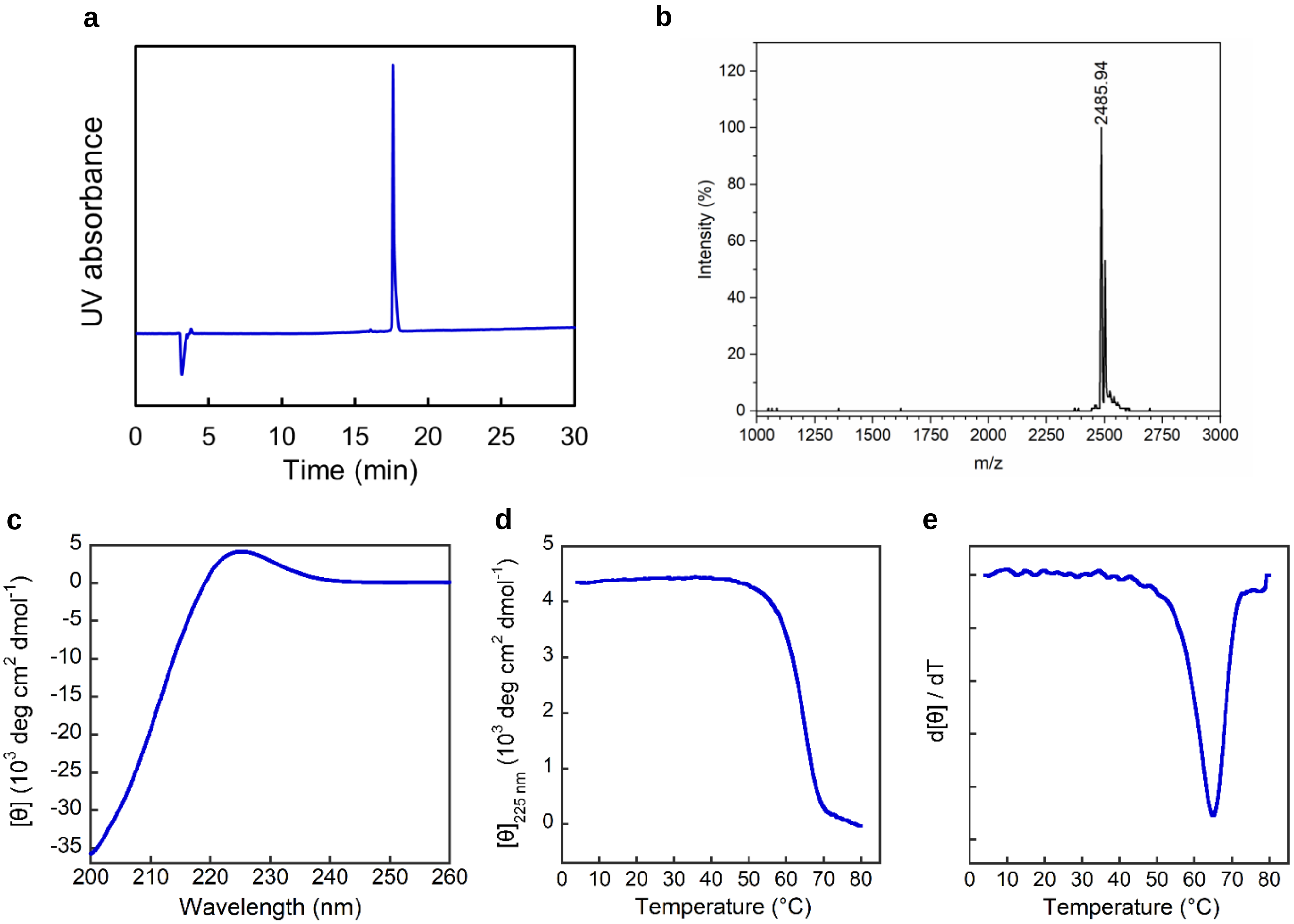

a, The HPLC chromatogram of purified peptide, t_R = 17.6 min. The purity is 99%.

b, MALDI-MS, calculated: 2486.1 [M+Na]$^+$, observed: 2485.9 [M+Na]$^+$, 2501.9 [M+K]$^+$.

c, The CD spectrum in PBS buffer at 4 °C.

d, The CD thermal melting curve in PBS buffer.

e, The first derivative of the melting curve, T_m = 65 °C.

Ac-(GPO)$_5$-NH$_2$

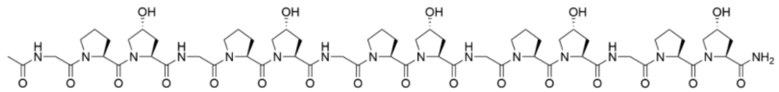

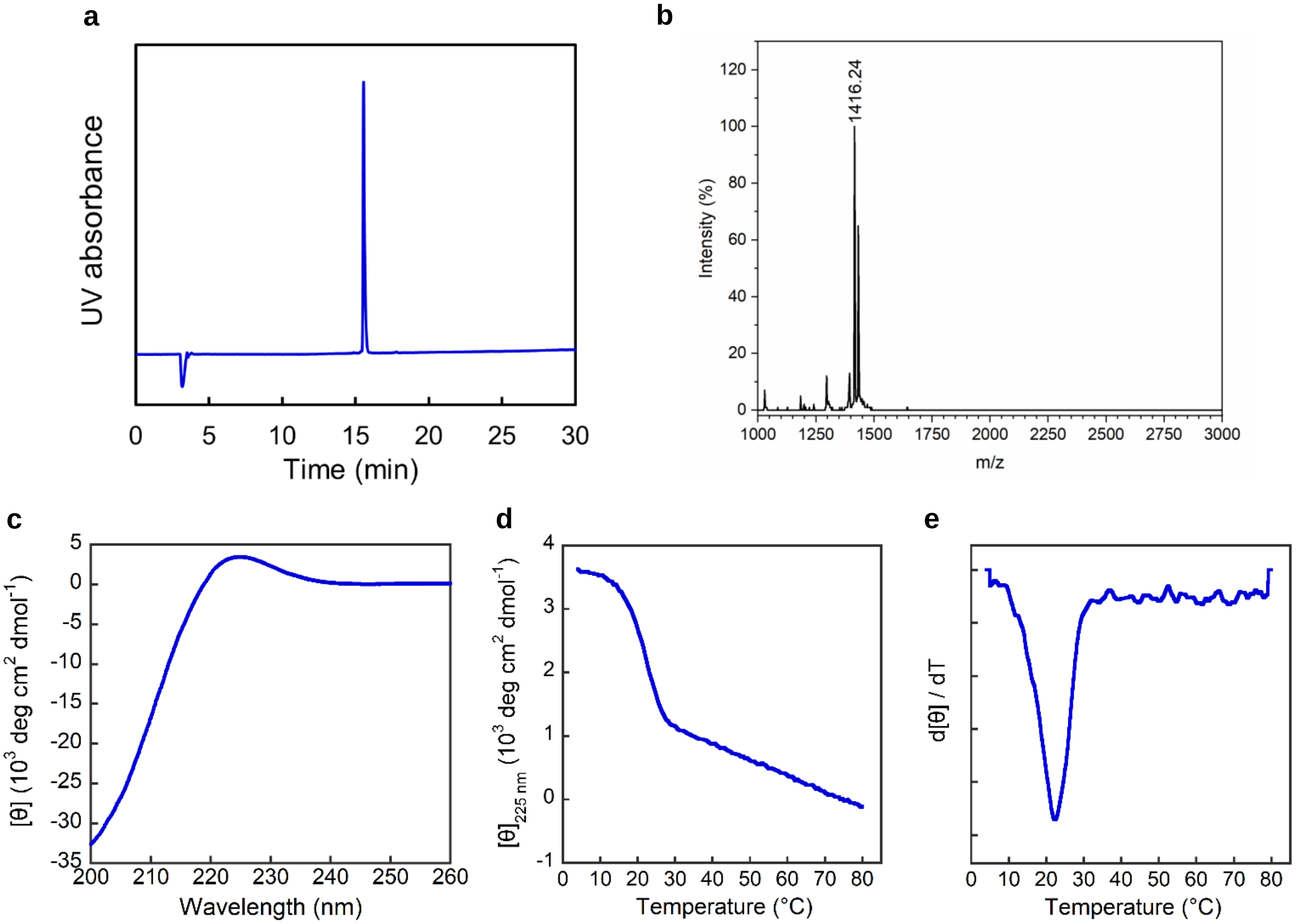

a, The HPLC chromatogram of purified peptide, t_R = 15.5 min. The purity is 99%.

b, MALDI-MS, calculated: 1417.7 [M+Na]$^+$, observed: 1416.2 [M+Na]$^+$, 1432.37 [M+K]$^+$.

c, The CD spectrum in PBS buffer at 4 °C.

d, The CD thermal melting curve in PBS buffer.

e, The first derivative of the melting curve, T_m = 23 °C.

Ac-(GPO)$_6$-NH$_2$

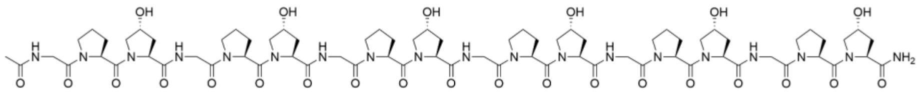

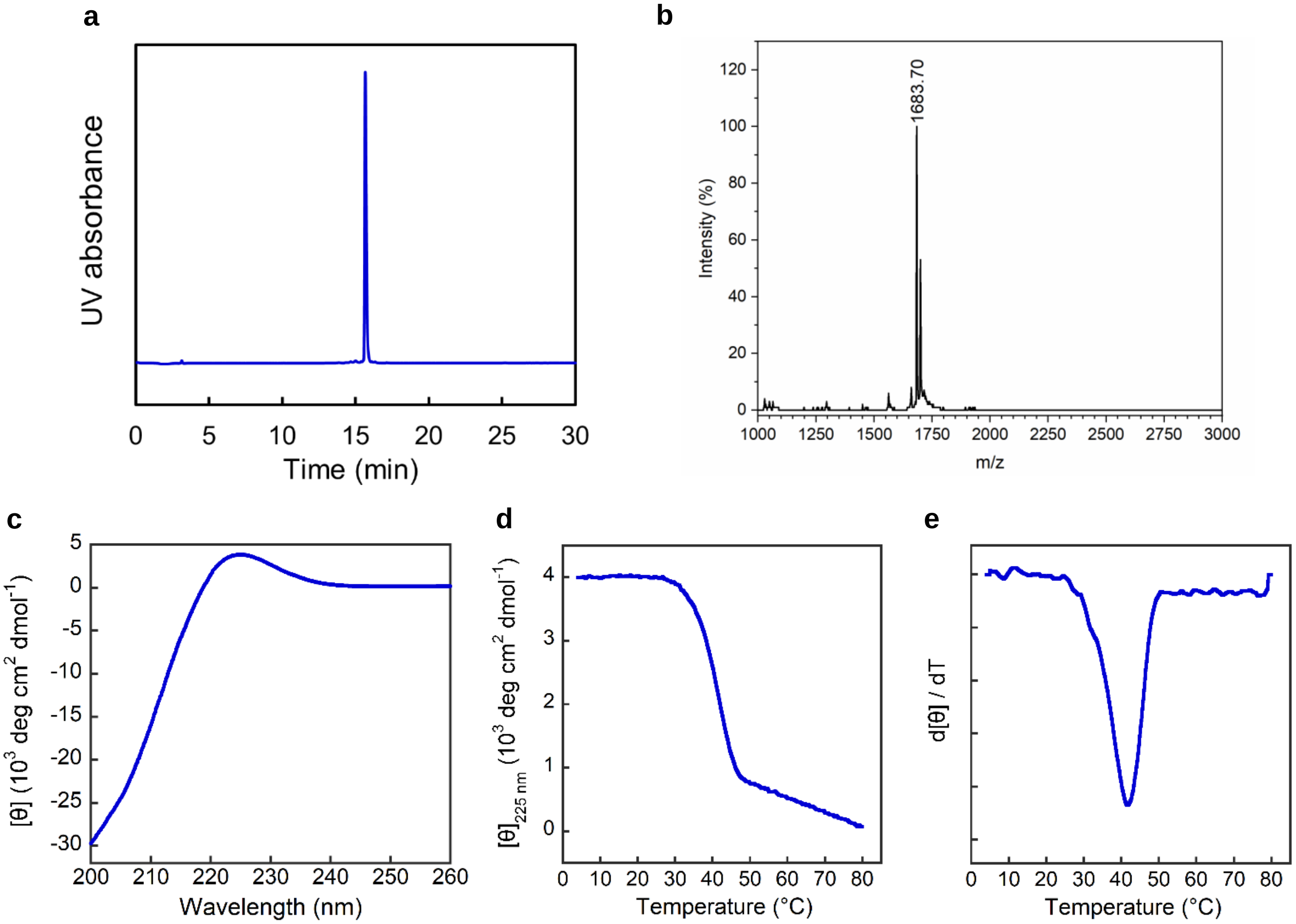

a, The HPLC chromatogram of purified peptide, t_R = 15.7 min. The purity is 99%.

b, MALDI-MS, calculated: 1684.8 $[M+Na]^+$, observed: 1683.7 $[M+Na]^+$, 1699.8 $[M+K]^+$.

c, The CD spectrum in PBS buffer at 4 °C.

d, The CD thermal melting curve in PBS buffer.

e, The first derivative of the melting curve, T_m = 42 °C.

H-(GPO)$_7$-NH$_2$

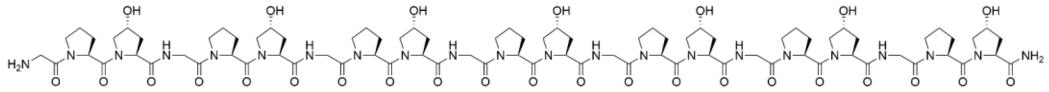

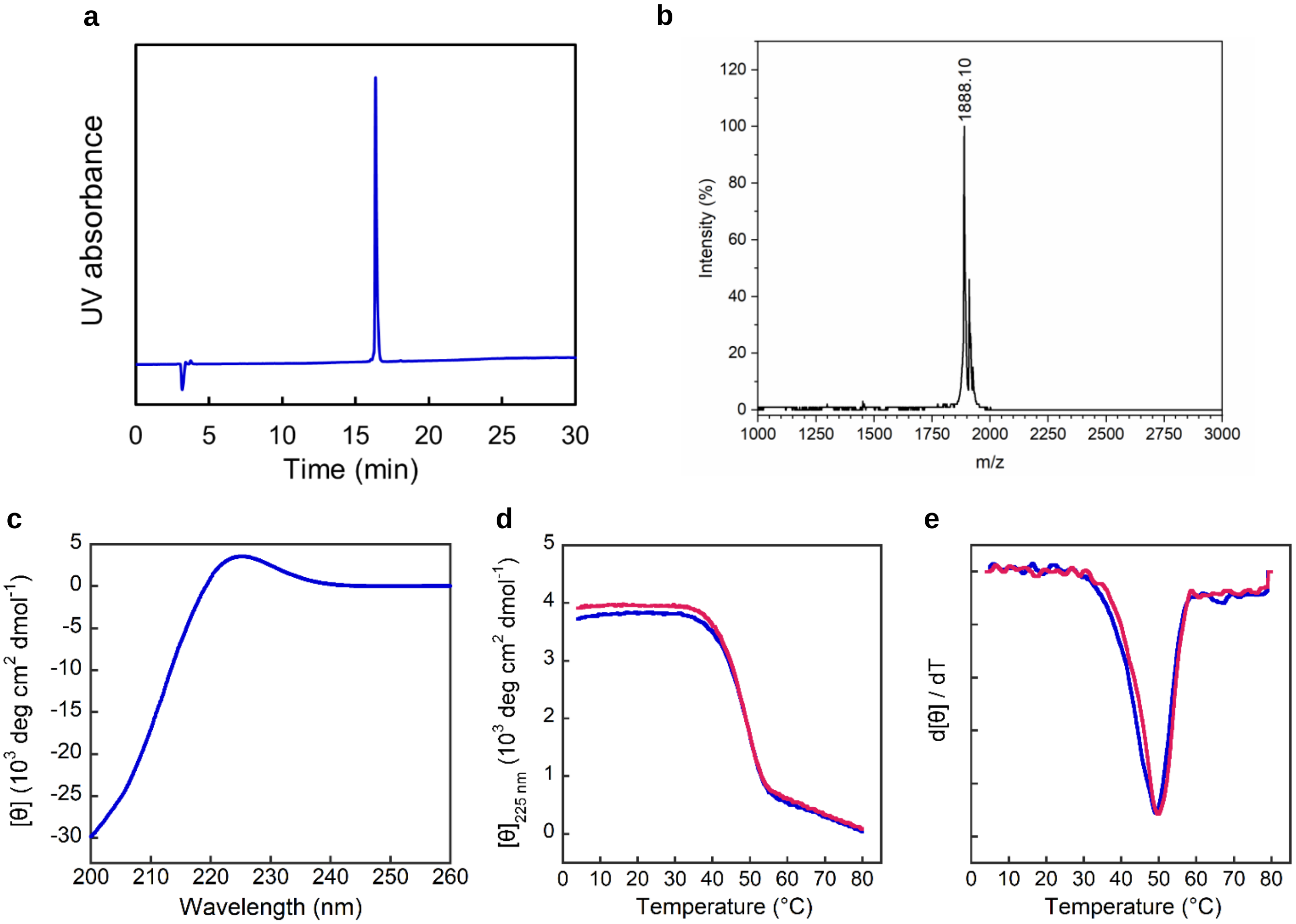

a, The HPLC chromatogram of purified peptide, t_R = 16.4 min. The purity is 99%.

b, MALDI-MS, calculated: 1887.9 [M+H]$^+$, observed: 1888.1 [M+H]$^+$, 1910.3 [M+Na]$^+$.

c, The CD spectrum in PBS buffer at 4 °C.

d, The CD thermal melting curve in PBS buffer (blue) and 3.5 mM NaOH solution (pH 11.5, red).

e, The first derivative of the melting curve, T_m = 49 °C (PBS, blue) or 50 °C (NaOH, red).

Ac-$(GPO)_8$-NH_2

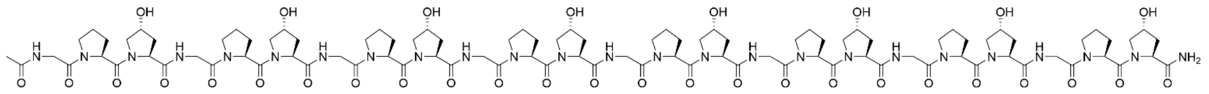

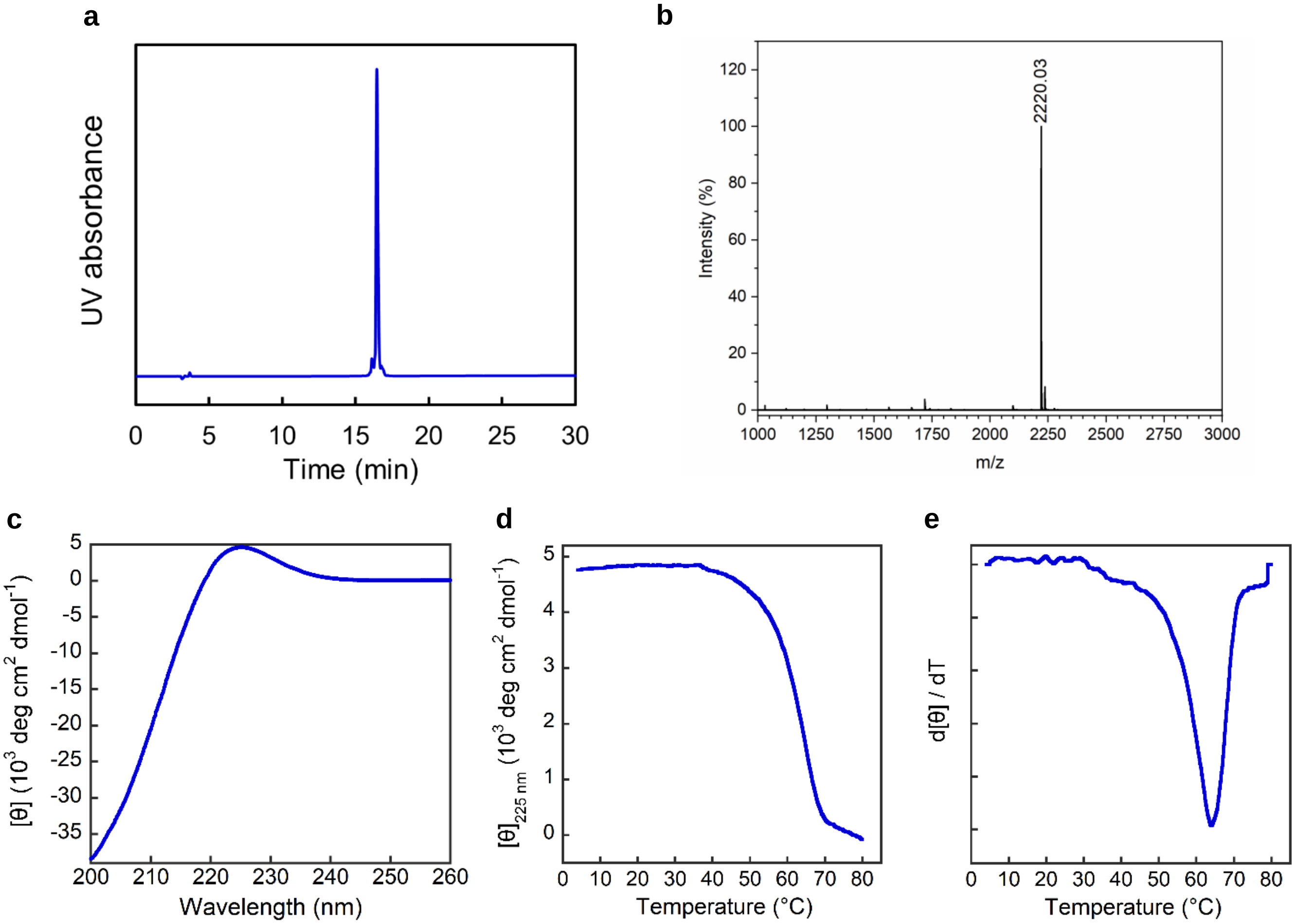

a, The HPLC chromatogram of purified peptide, t_R = 16.5 min. The purity is 96%.

b, MALDI-MS, calculated: 2219.0 $[M+Na]^+$, observed: 2220.0 $[M+Na]^+$.

c, The CD spectrum in PBS buffer at 4 °C.

d, The CD thermal melting curve in PBS buffer.

e, The first derivative of the melting curve, T_m = 64 °C.

Ac-(GPO)$_9$-NH$_2$

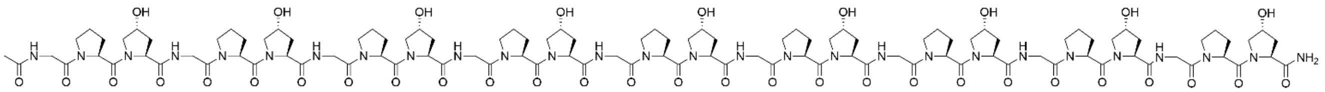

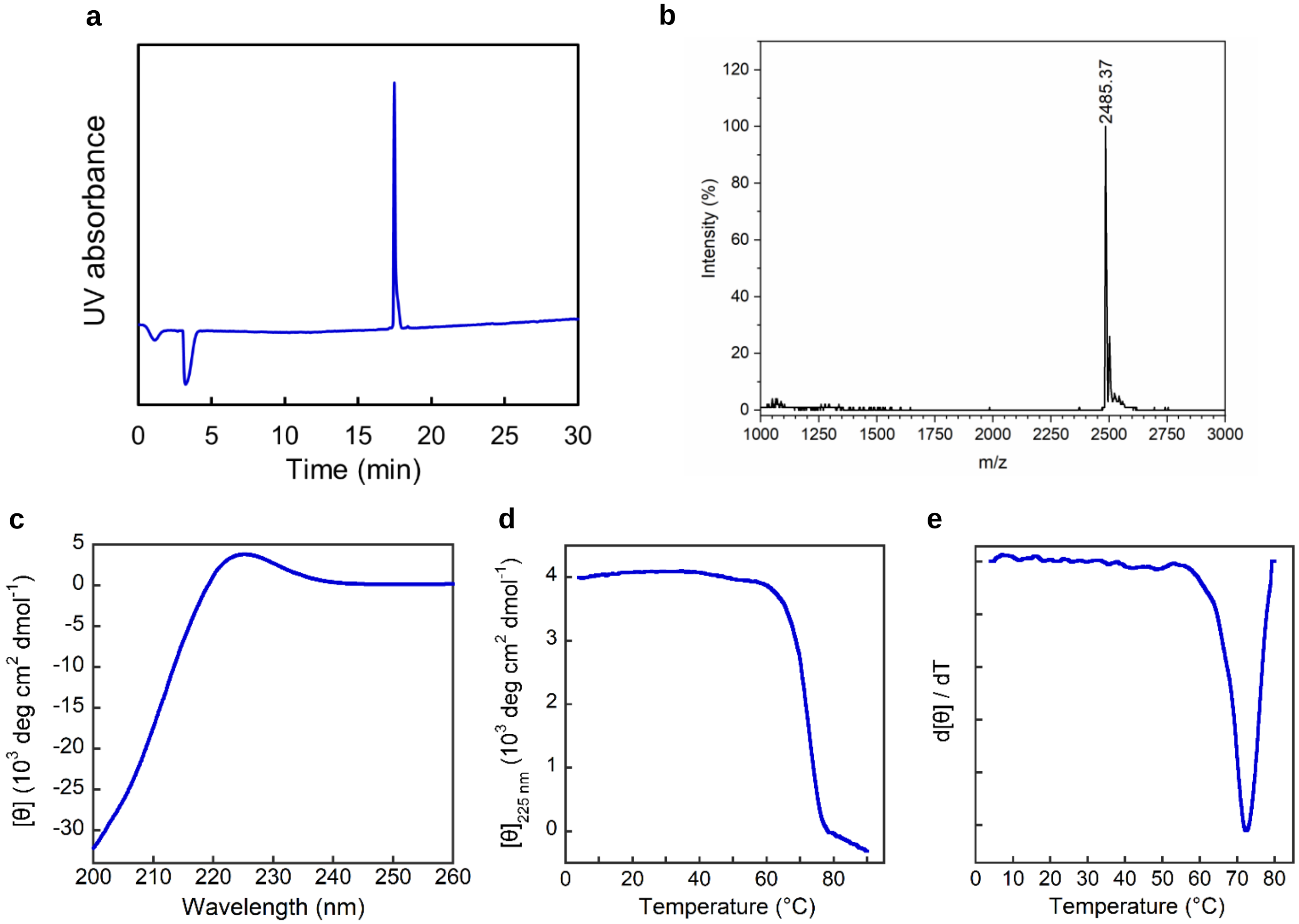

a, The HPLC chromatogram of purified peptide, t_R = 17.5 min. The purity is 99%.

b, MALDI-MS, calculated: 2486.1 $[M+Na]^+$, observed: 2485.4 $[M+Na]^+$, 2501.8 $[M+K]^+$.

c, The CD spectrum in PBS buffer at 4 °C.

d, The CD thermal melting curve in PBS buffer.

e, The first derivative of the melting curve, T_m = 72 °C.

H-(OGP)$_7$-NH$_2$

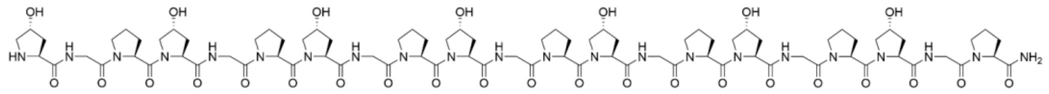

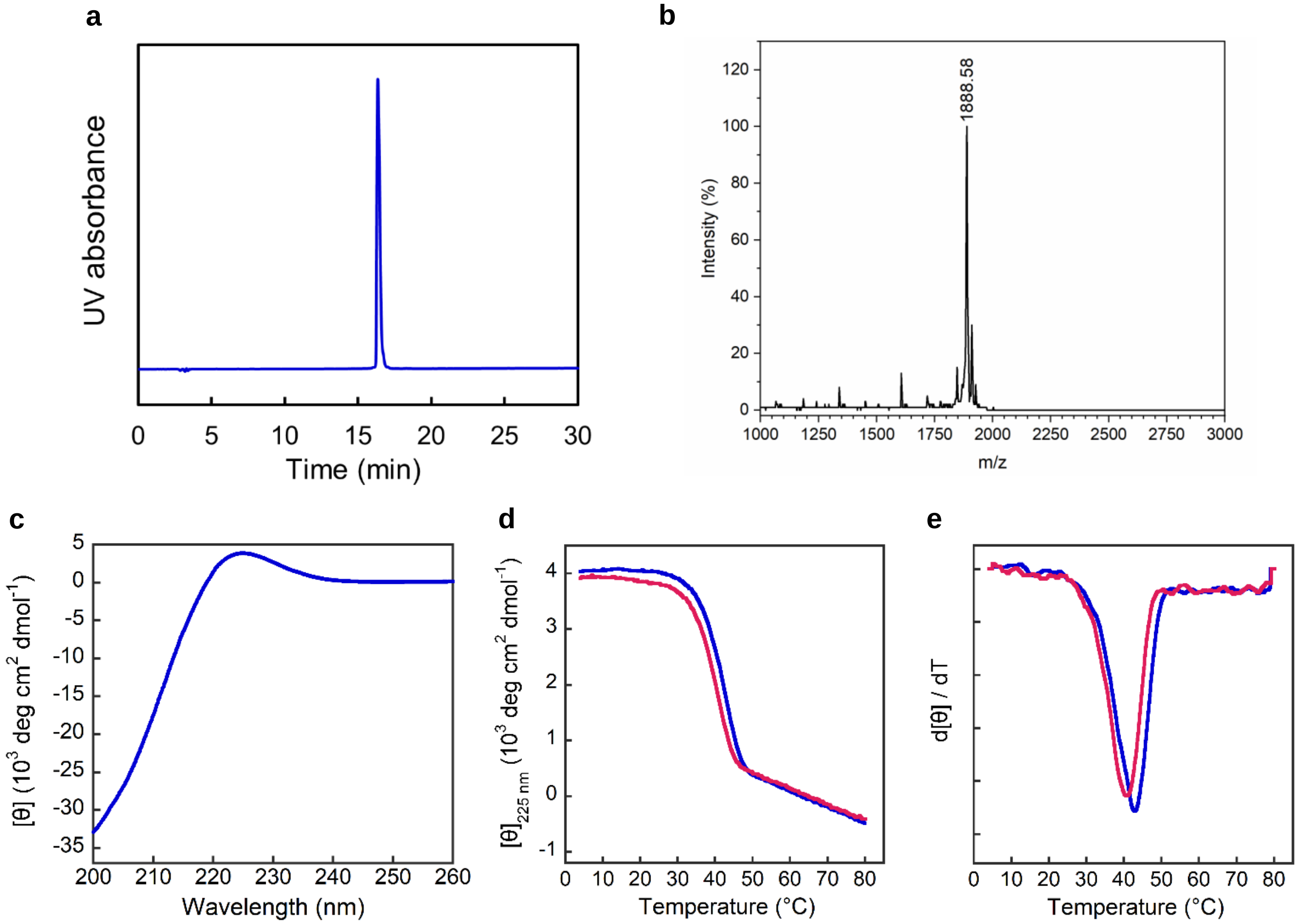

a, The HPLC chromatogram of purified peptide, t_R = 16.4 min. The purity is 99%.

b, MALDI-MS, calculated: 1887.9 $[M+H]^+$, observed: 1888.6 $[M+H]^+$, 1910.2 $[M+Na]^+$.

c, The CD spectrum in PBS buffer at 4 °C.

d, The CD thermal melting curve in PBS buffer (blue) and 3.5 mM NaOH solution (pH 11.5, red).

e, The first derivative of the melting curve, T_m = 43 °C (PBS, blue) or 40 °C (NaOH, red).

Ac-(OGP)$_7$-OH

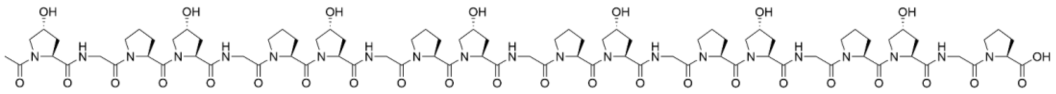

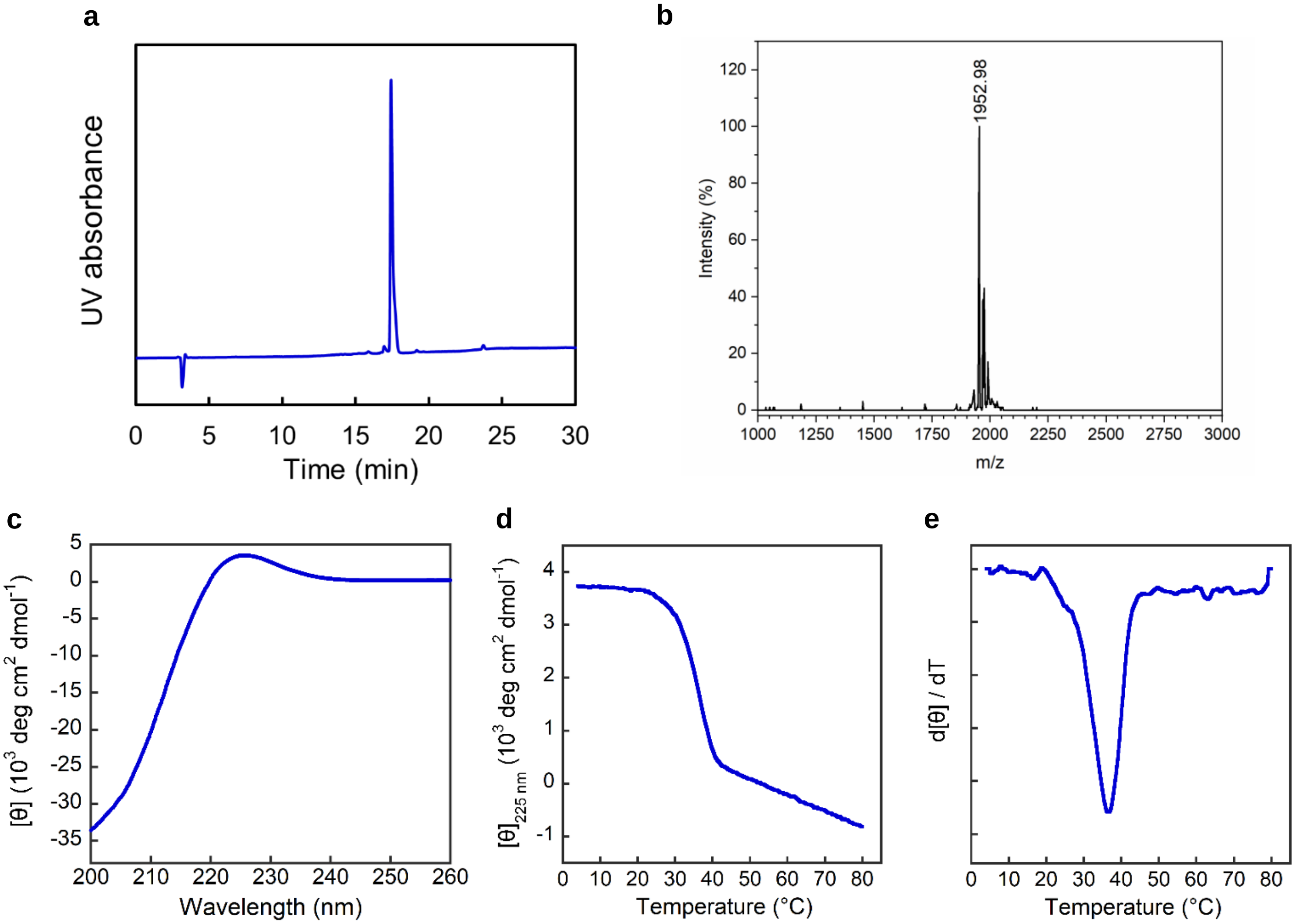

a, The HPLC chromatogram of purified peptide, t_R = 17.4 min. The purity is 97%.

b, MALDI-MS, calculated: 1952.9 $[M+H]^+$, observed: 1953.0 $[M+Na]^+$, 1975.03 $[M+K]^+$.

c, The CD spectrum in PBS buffer at 4 °C.

d, The CD thermal melting curve in PBS buffer.

e, The first derivative of the melting curve, T_m = 36 °C.

Ac-(OGP)$_8$-NH$_2$

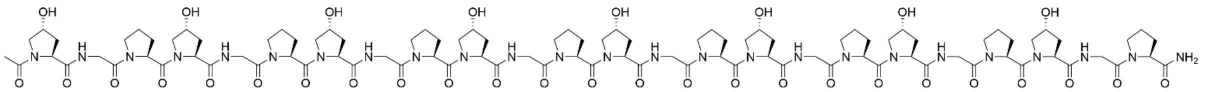

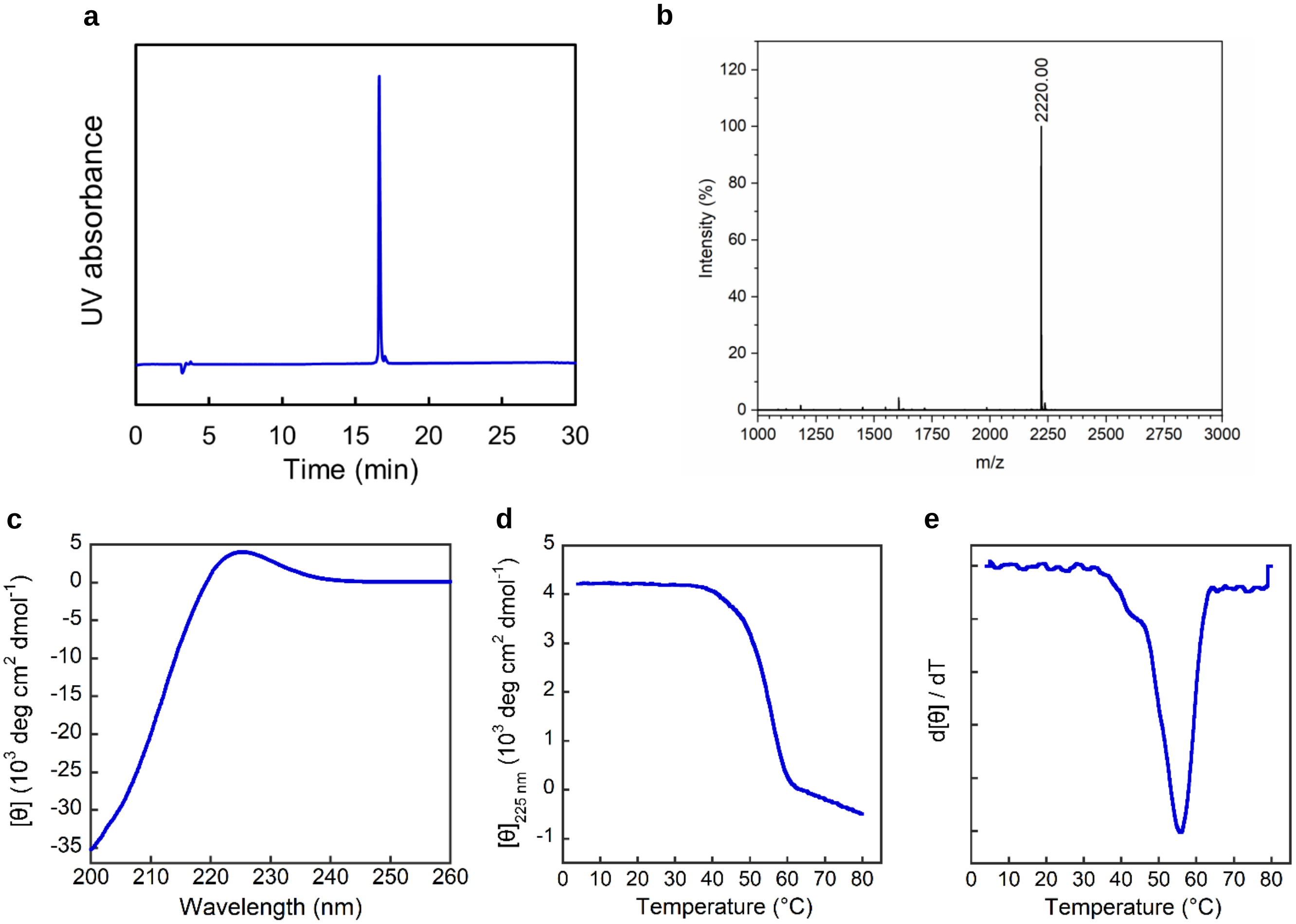

a, The HPLC chromatogram of purified peptide, t_R = 16.6 min. The purity is 97%.

b, MALDI-MS, calculated: 2219.0 [M+Na]$^+$, observed: 2220.0 [M+Na]$^+$.

c, The CD spectrum in PBS buffer at 4 °C.

d, The CD thermal melting curve in PBS buffer.

e, The first derivative of the melting curve, T_m = 56 °C.

Ac-GP(OGP)$_7$-NH$_2$ = Ac-(GPO)$_7$GP-NH$_2$

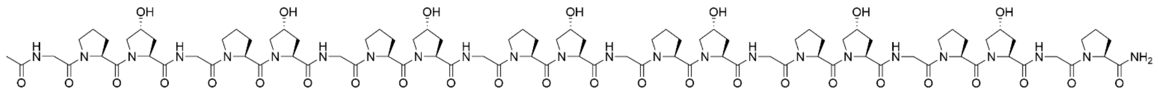

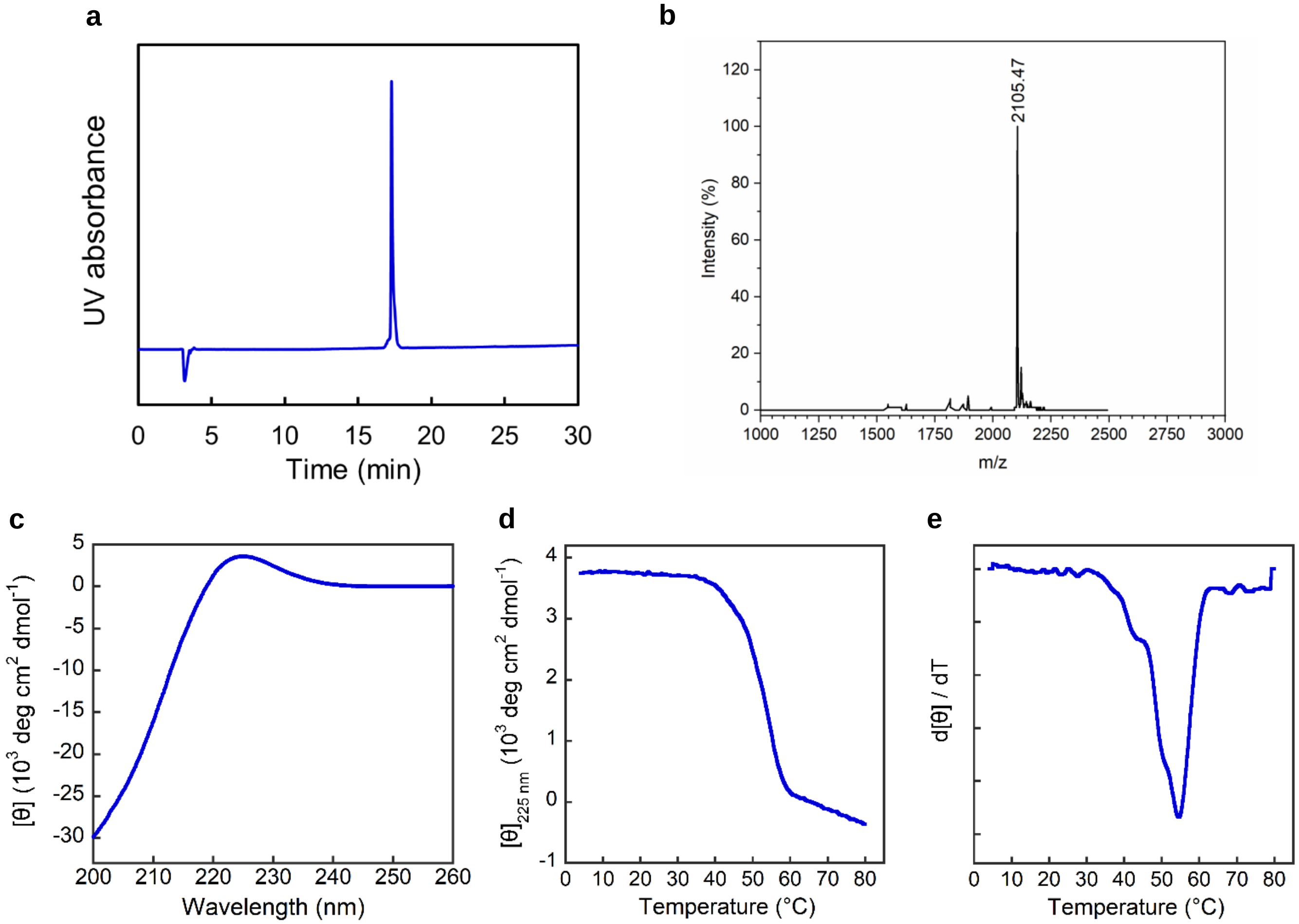

a, The HPLC chromatogram of purified peptide, t_R = 17.3 min. The purity is 96%.

b, MALDI-MS, calculated: 2106.0 $[M+Na]^+$, observed: 2015.5 $[M+Na]^+$, 2121.5 $[M+K]^+$.

c, The CD spectrum in PBS buffer at 4 °C.

d, The CD thermal melting curve in PBS buffer.

e, The first derivative of the melting curve, T_m = 54 °C.

Ac-PO(GPO)$_7$-NH$_2$ = Ac-(POG)$_7$PO-NH$_2$

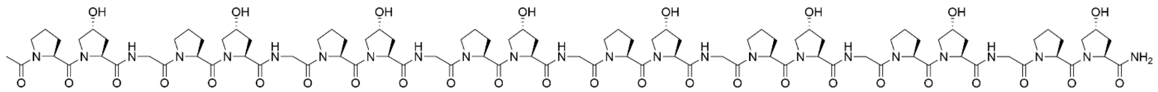

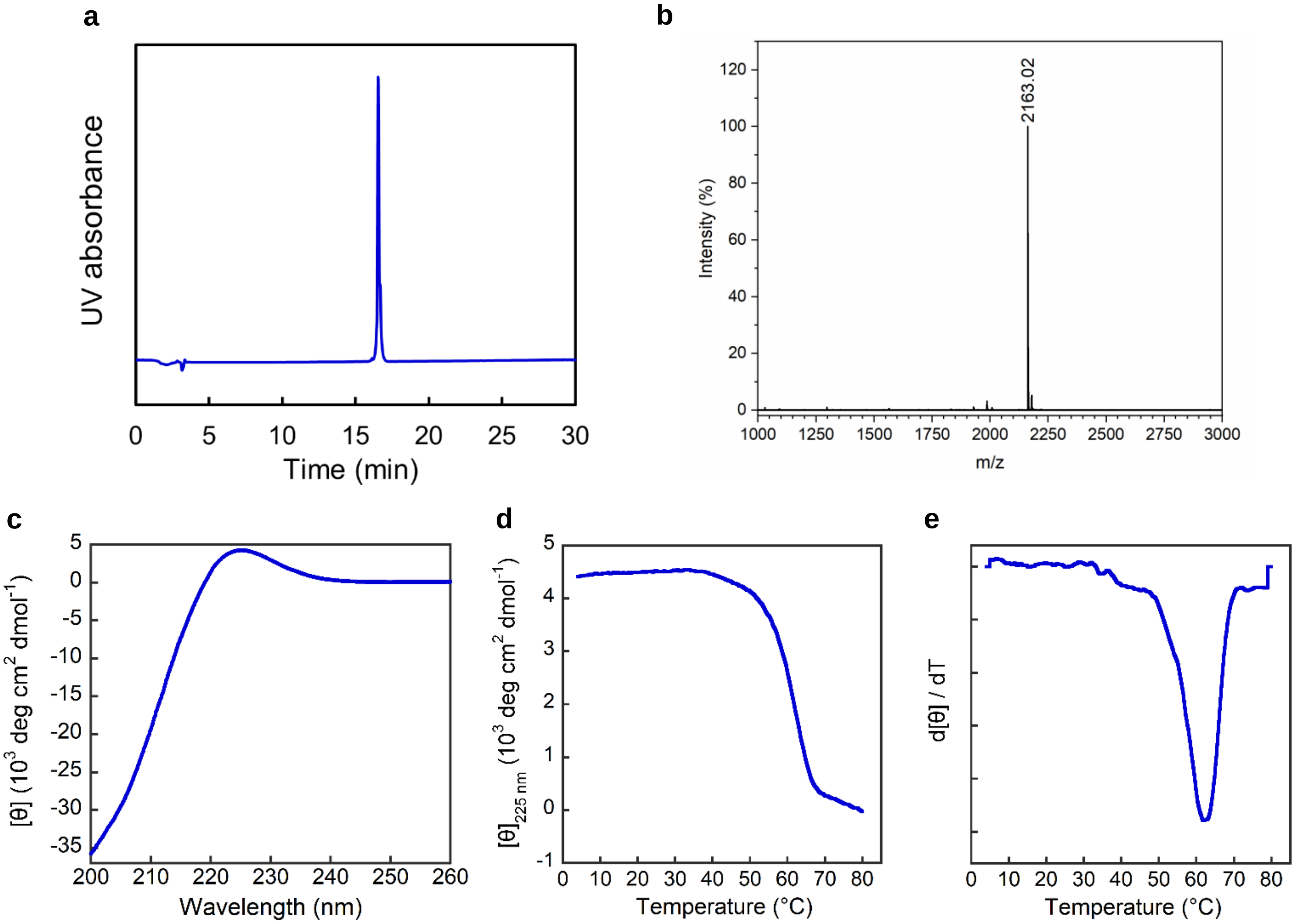

a, The HPLC chromatogram of purified peptide, t_R = 17.3 min. The purity is 96%.

b, MALDI-MS, calculated: 2162.0 $[M+Na]^+$, observed: 2163.0 $[M+Na]^+$.

c, The CD spectrum in PBS buffer at 4 °C.

d, The CD thermal melting curve in PBS buffer.

e, The first derivative of the melting curve, T_m = 62 °C.

Ac-OG(POG)$_7$-NH$_2$ = Ac-(OGP)$_7$OG-NH$_2$

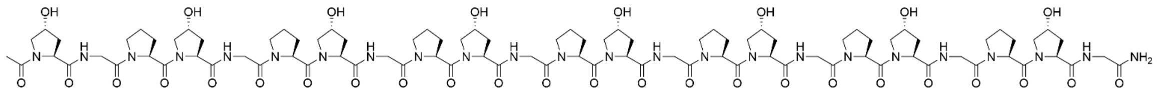

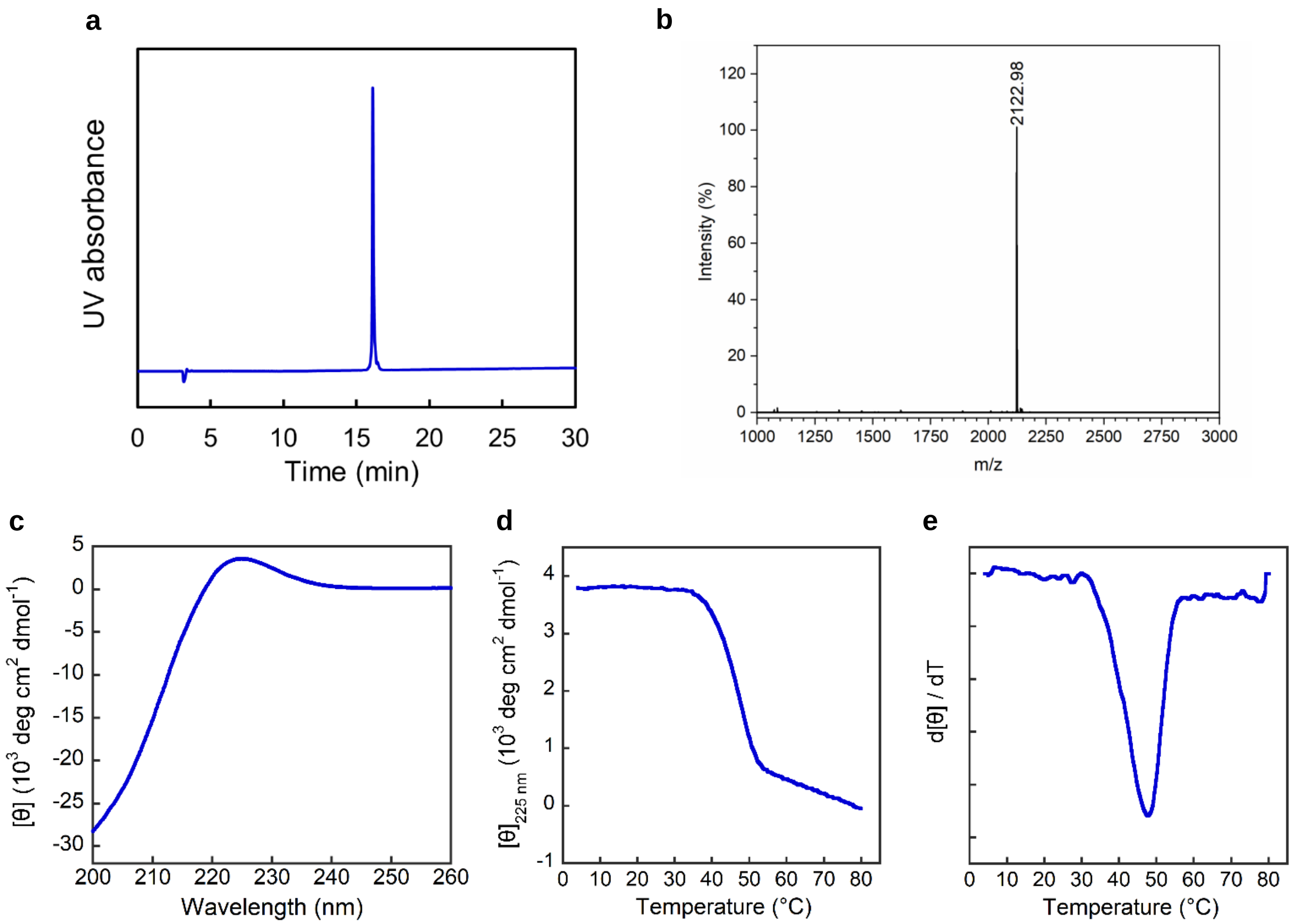

a, The HPLC chromatogram of purified peptide, t_R = 16.1 min. The purity is 96%.

b, MALDI-MS, calculated: 2122.0 $[M+Na]^+$, observed: 2123.0 $[M+Na]^+$.

c, The CD spectrum in PBS buffer at 4 °C.

d, The CD thermal melting curve in PBS buffer.

e, The first derivative of the melting curve, T_m = 48 °C.

References

(1) Kessler, J. L.; Kang, G.; Qin, Z.; Kang, H.; Whitby, F. G.; Cheatham, T. E., 3rd; Hill, C. P.; Li, Y.;Yu, S. M. Peptoid residues make diverse, hyperstable collagen triple-helices. *J. Am. Chem. Soc.* **2021,** *143* (29), 10910-10919.

(2) Nelson, M. T.; Humphrey, W.; Gursoy, A.; Dalke, A.; Kalé, L. V.; Skeel, R. D.;Schulten, K. NAMD: a parallel, object-oriented molecular dynamics program. *Int J High Perform Comput Appl.* **1996,** *10* (4), 251-268.

(3) MacKerell, A. D.; Bashford, D.; Bellott, M.; Dunbrack, R. L.; Evanseck, J. D.; Field, M. J.; Fischer, S.; Gao, J.; Guo, H.; Ha, S.; Joseph-McCarthy, D.; Kuchnir, L.; Kuczera, K.; Lau, F. T.; Mattos, C.; Michnick, S.; Ngo, T.; Nguyen, D. T.; Prodhom, B.; Reiher, W. E.; Roux, B.; Schlenkrich, M.; Smith, J. C.; Stote, R.; Straub, J.; Watanabe, M.; Wiórkiewicz-Kuczera, J.; Yin, D.;Karplus, M. All-atom empirical potential for molecular modeling and dynamics studies of proteins. *J Phys Chem B.* **1998,** *102* (18), 3586-3616.

(4) O'Leary, L. E. R.; Fallas, J. A.;Hartgerink, J. D. Positive and negative design leads to compositional control in AAB collagen heterotrimers. *J. Am. Chem. Soc.* **2011,** *133* (14), 5432-5443.

(5) Shoulders, M. D.; Satyshur, K. A.; Forest, K. T.;Raines, R. T. Stereoelectronic and steric effects in side chains preorganize a protein main chain. *Proc. Natl. Acad. Sci. U. S. A.* **2010,** *107* (2), 559-564.

(6) Bennink, L. L.; Li, Y.; Kim, B.; Shin, I. J.; San, B. H.; Zangari, M.; Yoon, D.;Yu, S. M. Visualizing collagen proteolysis by peptide hybridization: From 3D cell culture to in vivo imaging. *Biomaterials* **2018,** *183*, 67-76.

(7) Hwang, J.; Huang, Y.; Burwell, T. J.; Peterson, N. C.; Connor, J.; Weiss, S. J.; Yu, S. M.;Li, Y. In situ imaging of tissue remodeling with collagen hbridizing peptides. *ACS Nano* **2017,** *11* (10), 9825-9835.

(8) Liu, L.; Huang, K.; Li, W.; Qiu, R.; Fang, Y.; Huang, Y.; Zhao, S.; Lv, H.; Zhang, K.; Shan, H.;Li, Y. Molecular imaging of collagen destruction of the spine. *ACS Nano* **2021,** *15* (12), 19138-19149.
